# GUTchetp: integrated prediction of gut microbial biotransformation profiles using ensemble monolingual and multilingual neural machine translation and enzyme class consistency–reaction similarity

**DOI:** 10.1080/19490976.2026.2726639

**Published:** 2026-09-12

**Authors:** Masun Nabhan Homsi, Martin von Bergen

**Affiliations:** a Department of Molecular Toxicology, Helmholtz Centre for Environmental Research (UFZ), Leipzig, Germany; b Institute of Biochemistry, Faculty of Life Sciences, Leipzig University, Leipzig, Germany; c German Centre for Integrative Biodiversity Research (iDiv) Halle-Jena-Leipzig, Puschstraße, Leipzig, Germany

**Keywords:** Microbiome biotransformation product prediction, gut enzymatic reactions and microbial species prediction, gut microbial metabolic profile prediction, monolingual and multilingual neural machine translation, ensemble model, adaptive data augmentation, multiple molecular representations, mixed Fine-tuning, random forest, reaction similarity matrix

## Abstract

The analysis of the metabolic fate of chemical compounds in the human gut remains a fundamental challenge, as comprehensive experimental characterization across the vast chemical space is infeasible. Moreover, despite progress in computational biology, a comprehensive tool for predicting microbe-dependent metabolism of diverse chemicals is still lacking. To address these challenges, we introduce the GUTchetp framework, which comprises two components: one predicts gut biotransformation products and was tested on a benchmark of six compound categories, whereas the other identifies the associated microbial enzymes and species and was evaluated on a benchmark of 95 metabolism events. The first component employs an ensemble of monolingual and multilingual neural machine translation models, integrating transfer learning, mixed fine-tuning, multiple molecular representations, and adaptive data augmentation. The ensemble model outperformed previous approaches, achieving a Top-20 BLEU score and MaxFrag accuracy of 66.59% and 67.19%, respectively. The second component applies a re-ranking rule that combines the prediction consistency between two Enzyme Commission (EC) number classifiers with chemical reaction similarity, increasing accuracy by 34.38 percentage points over existing tools on the hidden dataset. Both components showed statistically significant improvements compared with existing tools, enabling the accurate prediction of 68.42% of known gut microbial biotransformation profiles, which is highly promising for anticipating gut microbial biotransformation outcomes. GUTchetp will thus pave the way for predicting the capacity of personalized gut microbiomes to metabolize intentional xenobiotics, such as pharmaceuticals and nutrients, and unintentional ones, such as environmental chemicals.

## Graphical abstract

**Figure:**
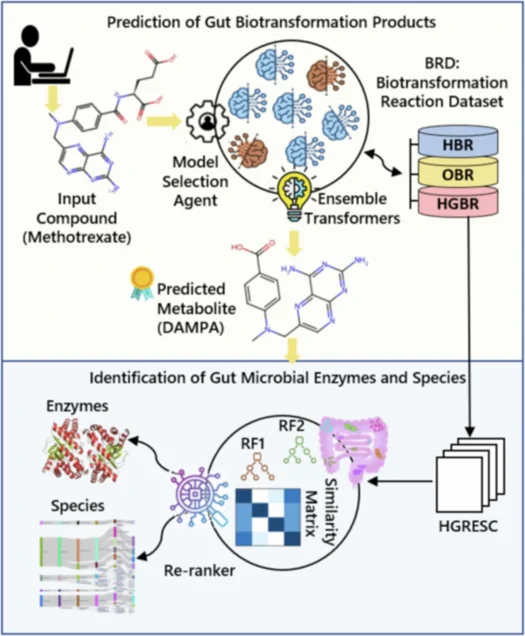

## 1. Introduction

The human gut microbiome is a living and dynamic ecosystem within the gastrointestinal tract made up of trillions of bacteria that fulfill many metabolic functions crucial for maintaining a healthy microbiome-host interaction. Among these functions are the digestion of proteins and otherwise hard-to-digest complex carbohydrates, and the production of vitamins, precursors of neurotransmitters, and metabolites such as short-chain fatty acids or bile acids.^1^ The long coevolution of host and microbiome has led to multiple metabolite-based microbiome-host interactions ranging from the relevance of microbial-derived precursors of neurotransmitters over the signaling of microbially modified bile acids for glucose and lipid metabolism in the liver to the involvement of short-chain fatty acids in regulating T-cell differentiation.^2^ The key to the wide versatility of the metabolism in the microbiome lies in the many different ecological niches, which exist in the gastrointestinal tract either physically (peristaltic), chemically (pH, O2 concentration), or biochemically (presence of host-derived factors like anti-microbial immunoglobulins, mucin, and availability of ingested diet).^3^ These multiple niches explain the presence of a great variability of species with an extreme and otherwise not known extent of microbial metabolic versatility in a habitat.^4^

This metabolic versatility provides the basis not only for the multitude of catalyzed natural metabolic activities but also for the metabolization of novel entities that enter the gastrointestinal tract in modern life. The most comprehensive exposome approach has yielded up to 41,474 different compounds in human blood.^5^ The organic compounds among them can be categorized into three main groups characterized by the degree of intentional use. First, there are those that are taken up fully intentionally, such as pharmaceuticals or sweeteners. The second group includes substances such as the widely used food additives or secondary plant metabolites, which could only be avoided by adopting a selective diet. The third group consists of those that are taken up definitely unintentionally, such as plant protection products. There are reports from representatives of all three groups that their interaction with the microbiome leads to changes of the structure and function of the microbiome, with adverse effects on the microbiome host-interaction.^6^

The first step is the biotransformation of non-natural chemicals by microbial enzymes. In some cases, this is exploited in the design of prodrugs, e.g. sulfasalazine, which is activated in the intestine.^7^ But in other reported cases, microbial biotransformation leads to an inactivation, as in the first reported case of digoxin.^7^ For food additives, a major group of representatives from the second category, it is known that they can affect the structure and functionality of the intestinal microbiome.^8^ At the same time, food additives such as emulsifiers, preservatives, and artificial colors can be transformed into bioactive products. The same applies to the group of unintentional chemicals like plant protection products.^9^ ^,^ ^10^ Given the extremely wide range of chemicals humans are exposed to today, actually there are more than 350.000 chemicals present in the environment in total,^11^ and in the US alone about 700 new chemicals are produced,^11^ it is obvious that experimental testing alone cannot solve the problem.^12^ On the host side, there is a good overview about its capacity to biotransform xenobiotics, especially for the group of pharmaceuticals,^13^ but knowledge of the intestinal microbiome's capacity to biotransform xenobiotics is limited.

In recent studies, it was shown that for a group of 271 orally admitted pharmaceuticals, about two-thirds were reduced when 76 different bacterial strains were used to assess the potential for biotransformation.^14^ So far, no studies are available in which a larger set of experimentally tested orally administered drugs, food additives, or the large group of plant protection products has been analyzed.

So far, experimental testing has been performed with a limited selection of microbial species and under conditions that differ from the *in vivo* situation. However, due to ethical and practical restrictions on in *in vivo* testing, there is a strong demand for applying *in silico* approaches to predict biotransformation by the intestinal microbiome. Thereby, these computational approaches can support early-stage screening, prioritization of chemicals for experimental validation, and the formulation of testable hypotheses on microbiome-mediated transformation pathways prior to *in vitro* or *in vivo* studies.

The basis for any prediction of microbial metabolic capacity is the knowledge of the natural activity within the intestinal microbiome, which is accessible by combining metagenome information and existing databases of enzymatic properties, as demonstrated by MelonnPan (Model-based Genomically Informed High-dimensional Predictor of Microbial Community Metabolic Profiles). This is an example of a computational tool that predicts community metabolomes from microbial community profiles, extracted from amplicons, metagenomes, or metagenomics functional profiles.^15^

The state of the art is deep learning (DL) algorithms, which are considered machines that mimic the human brain to solve complex real-world problems,^16^ such as the identification of biotransformation reaction products without exhaustive wet-lab experimentation. Machine translation (MT) is a subfield of computational linguistics that aims to create software capable of automatically translate text or speech from one language to another without the need for human intervention. With the boom of DL over the past decade, neural machine translation (NMT) has emerged and been widely used due to its impressive performance and remarkable achievements in translation quality.^17^ NMT encompasses both monolingual and multilingual (MNMT) models.^18^

Several NMT-based approaches have been developed for predicting chemical reactions, involving forward synthesis and retrosynthesis prediction, where the SMILES (Simplified Molecular Input Line Entry System) notation is usually used for molecule representation.^19^ ^,^ ^20^ For instance, the metabolite translator (MetaTrans) was introduced for predicting human metabolites from therapeutics.^21^ The authors utilized transfer learning on a deep learning transformer for sequence translation, trained their model on human chemical reaction data, and tested it using 84 drugs with 217 metabolites covering a wide collection of enzymes. In addition, an ensemble model was built to select the metabolite from the different six fine-tuned models. Similar two approaches were introduced to predict the outcome of Baeyer-Villiger and carbohydrate reactions,^19^ ^,^ ^22^ respectively. In contrast, MetaPredictor goes beyond employing deep learning transformers and ensemble learning^23^; it also incorporates prompt-based learning by embedding specific annotations within the SMILES string of parent molecules. The authors demonstrated that prompt engineering could steer a deep language model to generate more accurate metabolite predictions, outperforming four other tools, namely GLORYx,^24^ SyGMa,^25^ BioTransformer^26^ and MetaTrans.^21^ MetaPredictor was tested on 135 drugs with 283 identified human metabolites, achieving up to a 5% improvement in Top-5 prediction accuracy compared to MetaTrans.^23^

Determining the gut-derived metabolites is only the first step; identifying the enzymes and microbial species responsible for gut biotransformation is equally important. Tools such as SIMMER (Similarity algorithms that Identify MicrobioMe Enzymatic Reactions) were developed for this purpose. SIMMER leverages a reaction vector-embedding approach based on the MetaCyc database to compare metabolic reactions, coupled with protein similarity searches to identify homologous enzymes in gut bacterial genomes.^27^ This tool was validated *in vitro* with the gut bacterial metabolism of methotrexate and successfully predicted the enzyme involved in its transformation. In contrast, other tools have focused on predicting Enzyme Commission (EC) numbers from chemical reactions. One such framework is CLAIRE (Contrastive Learning-based AnnotatIon for Reaction's EC),^28^ which leverages contrastive learning and pre-trained language model-based reaction embeddings. However, these approaches continue facing limited coverage and low accuracy, primarily because of the complexity of gut chemistry and incomplete functional annotations.

Despite advances in computational biology and *in silico* modeling, no single tool can yet accurately predict gut biotransformation products along with the associated enzymes and microbial species in response to any chemical compounds. Most current approaches focus on only one aspect at a time, such as enzyme class, metabolite, or individual microbial species. A comprehensive framework that predicts biotransformation profiles is still lacking. Moreover, in the context of human or human-gut metabolite prediction applications, where continuous learning is crucial, very few datasets and benchmarks are available to evaluate and compare emerging techniques. MetaTrans and MetaPredictor have been only evaluated on a short list of drugs, leaving out other chemical categories that could influence human gut health. In addition, both tools are limited to a restricted structural transformation space. In particular, MetaTrans and MetaPredictor show reduced predictive performance for metabolites that undergo minimal loss of atomic composition (<25% reduction), whereas MetaTrans is also limited in predicting metabolites with substantial structural divergence from the parent molecule (fingerprint similarity < 0.25).

To address these limitations, in this study, we propose a new benchmark dataset, specifically designed for gut biotransformation product prediction (PP). Unlike earlier test datasets, our PP consists of six categories, including drugs, environmental chemicals, food additives, medicinal herbs, metabolites, and polyphenols. In addition, we developed GUTchetp (GUT microbial and chemical biotransformation profile) to predict gut biotransformation products as well as the associated enzymes and microbial species for diverse chemical compounds. GUTchetp employs a transformer architecture with two steps of transfer learning (TR) and mixed fine-tuning (MFT) strategies. To enhance the predictive performance of this transformer model, we applied both standard and adaptive data augmentation in the experiments, denoted as A and Adapt, respectively. By representing a chemical reaction with multiple SMILES strings (Single Representation, SRP), the model gains deeper insights into the reaction by processing a batch of randomly generated SMILES strings. Even though these augmented SMILES strings encode the same chemical information, the model can capture more subtle features by constructing the reaction from diverse SMILES sequences. Furthermore, we also employed multilingual neural machine translation (MNMT) to improve prediction quality, particularly for those biotransformation reactions where the products differ significantly from the reactants. Thereby, data were augmented using a combination of different chemical multi-representation (MRP) structures in multiple notations, namely SMILES, InChI (International Chemical Identifier)^29^ and SELFIES (SELF-referencing embedded string)^30^; to leverage shared chemical patterns across representations and enable transfer learning and improve significantly translation quality. We further built an ensemble model by fine-tuning multiple neural machine translation algorithms on different subsets of the biotransformation reactions dataset (BRD), generated by a hierarchical clustering algorithm (See Section 2.2.1.2). BRD is our training dataset, which was manually curated from scientific literature and integrated with the Unified Human Gastrointestinal Genome (UHGG) collection.^31^ Moreover, GUTchetp operates at four complexity levels with varying performance characteristics: basic, advanced, exhaustive, and adaptive. It is equipped with an agent to select the appropriate set of base models prior to prediction, aiming to reduce prediction time while maintaining accuracy and robustness in identifying gut biotransformation products. Subsequently, GUTchetp uses a chemical similarity matrix to retrieve a list of reactions similar to the predicted one, which is then re-ranked employing a novel metric that integrates chemical similarity and the prediction consistency of two random forest-based enzyme classifiers for first-level and second-level EC (Enzyme Commission) numbers. The goal is to first identify enzymes that catalyze a diverse range of chemical reactions in the gut and then determine the associated microbial species. Finally, GUTchetp can automatically distinguish among the metabolic space of the gut microbiome, the human-gut microbiome, human metabolism, or both metabolisms. The novelty of our work is as follows:

(1) We introduced the GUTchetp model to predict gut biotransformation products, along with associated enzymes and gut microbiota, outperforming previously published approaches in terms of metabolite translation quality and accuracy of the core molecular structure;
(2) Our new approach, GUTchetp, substantially expands the detectable metabolic transformation space by capturing biologically valid transformations that lie outside the structural constraints of existing approaches.
(3) We employed a new method of data augmentation, named adaptive data augmentation, where the augmentation strategy is dynamically adjusted based on the chemical characteristics of the reaction data to improve model generalization;
(4) We built a hybrid ensemble model based on monolingual and multilingual neural machine translation methods to improve biotransformation product prediction, specifically for those gut reactions where the products are less similar to the parent compounds;
(5) The base models of GUTchetp were trained employing two steps of transfer learning along with a mixed fine-tuning strategy and chemical multi-representation structures; including SMILES, InChI, and SELFIES;
(6) Based on 160 molecular features, a comprehensive performance comparison of GUTchetp and previous approaches for gut biotransformation prediction was conducted;
(7) We built PP and EP, two new benchmark datasets for evaluating GUTchetp and other approaches for gut biotransformation products and enzyme prediction, respectively;
(8) We introduced a novel re-ranking metric that jointly incorporates prediction consistency across first- and second-level EC number enzyme classifiers and chemical reaction similarity for gut microbial enzymes and species identification.

## 2. Methods

**Figure 1.**
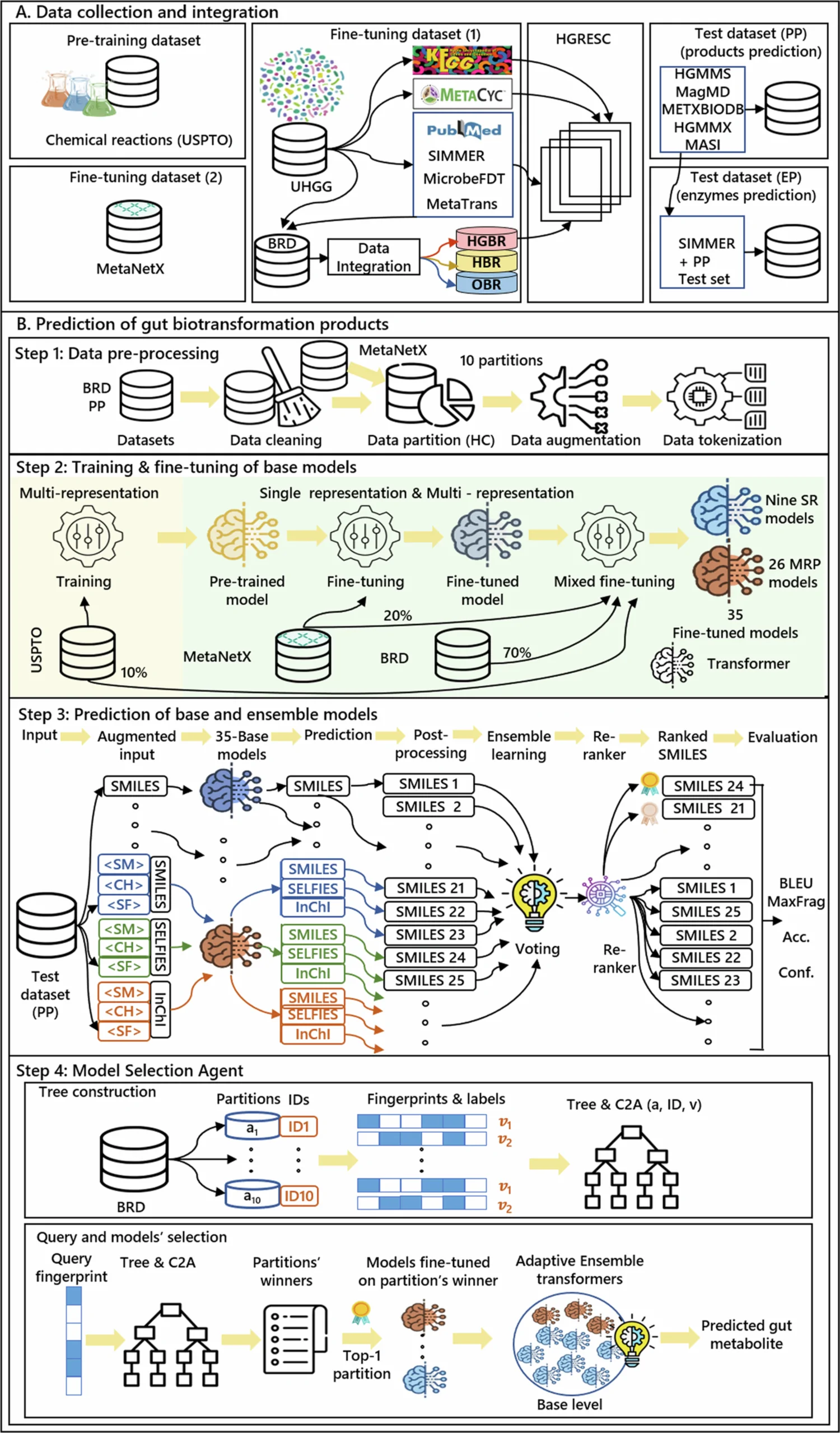
Methodology for building GUTchetp. (A) The construction of fine-tuning datasets: BRD and MetaNetX, two test datasets: PP for gut biotransformation product prediction and EP for enzyme prediction, and the Gut Microbial Reaction–Enzyme–Species Catalogue (HGRESC). (B) This flowchart illustrates the main steps to construct the first component of GUTchetp to predict gut biotransformation products, including data preprocessing, fine-tuning of base models, ensemble model, and the construction of a model selection agent. (C) This flowchart reflects the main steps to construct the second component of GUTchetp for the identification of enzymes and gut microbiota, including similarity matrix construction, re-ranker, and retrieval of gut reactions, enzymes, and gut microbiota.

**Figure 2.**
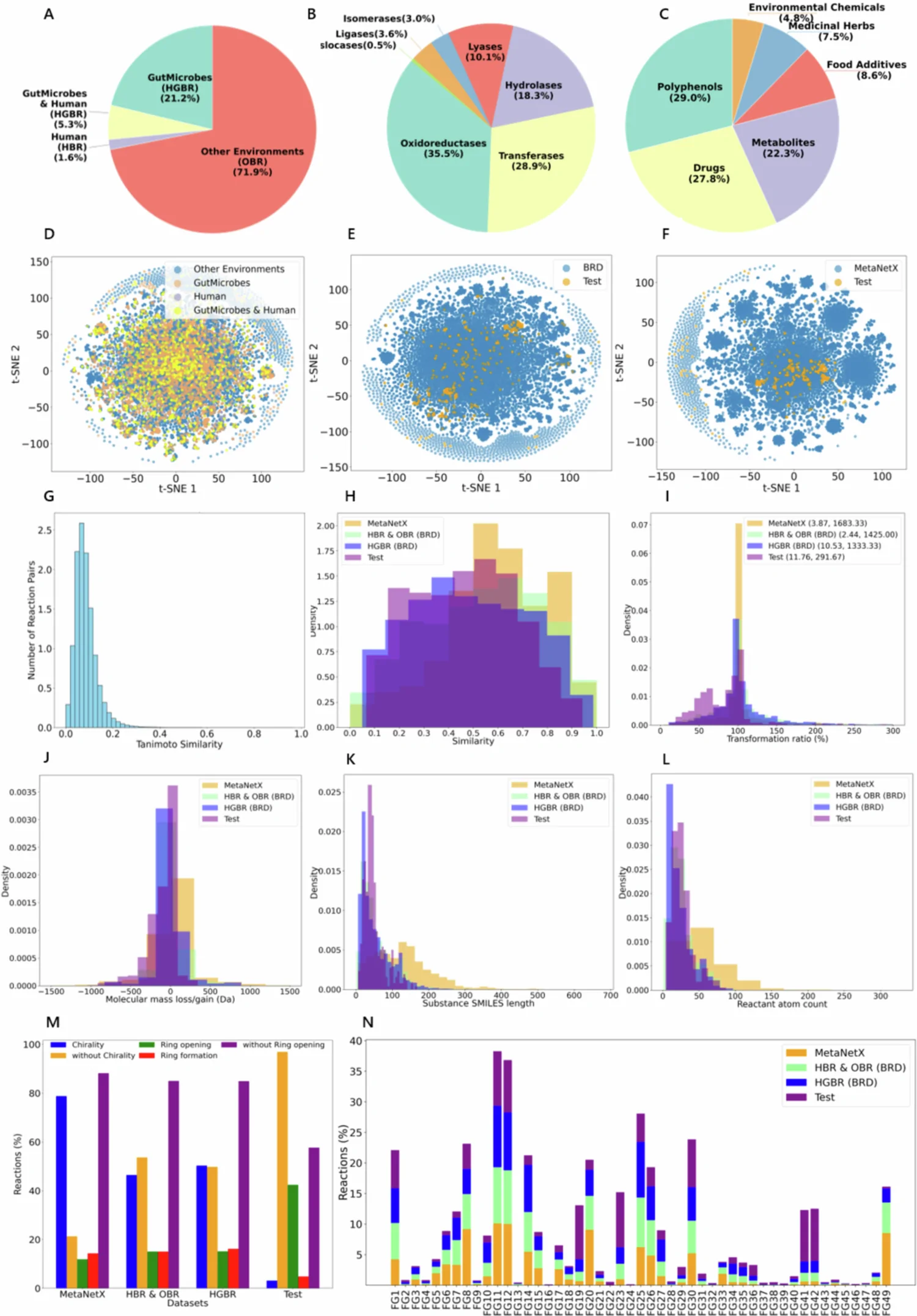
Chemical space of fine-tuning and test datasets. (A) Proportion of four types of biotransformation reactions in the BRD dataset. (B) Distribution of enzymatic reactions by first-level EC number in BRD. (C) Distribution of chemical compounds across categories in the test dataset (PP). (D) Chemical space of the biotransformation reactions from different environments. (E) Test dataset (PP) versus fine-tuning dataset (BRD). (F) Test dataset (PP) versus MetaNetX. (G) Pairwise Tanimoto similarity between the PP and BRD datasets. (H) Distribution of molecular similarity between reactants and products. (I) Distribution of transformation ratios. (J) Distribution of molecular mass changes. (K) Distribution of SMILES lengths for the reactants. (L) Distribution of the number of atoms in the reactants. (M) Comparative structural transformation types across datasets. (N) Distribution of 49 functional groups (FGs) across datasets. Supplementary Table S8 provides details of these 49 functional groups.

**Figure 3.**
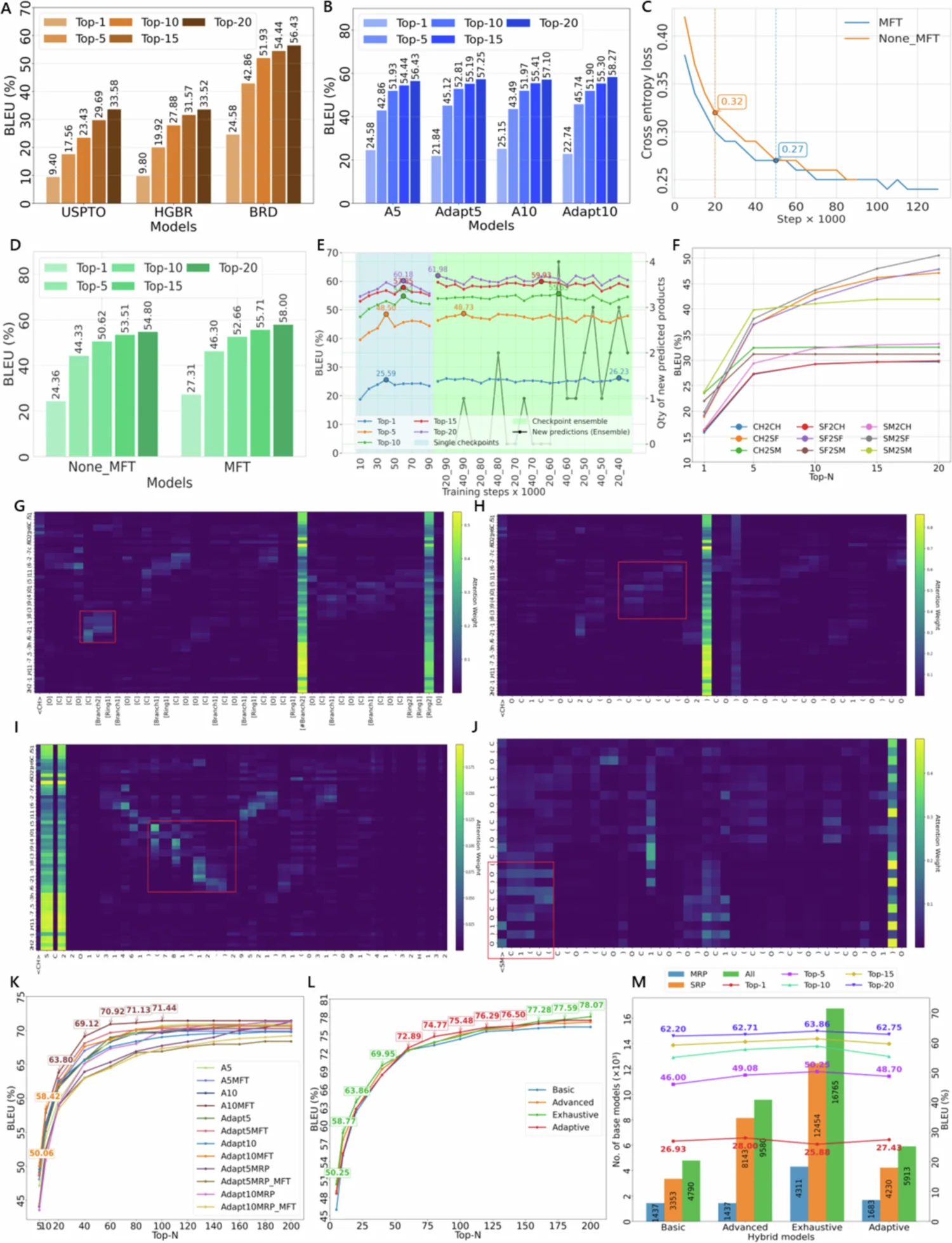
Predictive performance of the first component of GUTchetp: Base and ensemble model results. (A) Impact of data size. (B) Impact of adaptive augmentation. (C) Cross-entropy loss between MFT and None-MFT. (D) Impact of MFT. (E) Impact of checkpoint ensembles. (F) Impact of multi-representation molecular translation. (G−J) Attention heatmaps showing the correlation between Lactulose (horizontal) and predicted fructose (vertical) tokens in layer 4, averaged over eight heads: (G) SF2CH. (H) SM2CH. (I) CH2CH. (J) SM2SM. (K) Performance of ensemble models across 12 different configurations. (L) Hybrid ensemble performance across four prediction levels. (M) Trade-off between GUTchetp levels, number of base models, and computational time.

GUTchetp was developed as a three-module framework: (1) a data collection module (Figure 1A), (2) a gut biotransformation prediction module (Figure 1B), and (3) a module for identifying gut microbial enzymes and species involved in compound transformation (Figure 1C). The second and third modules represent the main two core components of GUTchetp.gest

### 2.1. Module I: data collection and integration

Four datasets were used to build and evaluate GUTchetp. Three of them were used for the prediction of gut biotransformation products, pre-training, fine-tuning, and test data (Figure 1A), while the fourth one was employed for enzyme prediction and gut microbes’ identification. In addition to that, we constructed the Gut Microbial Reaction–Enzyme–Species Catalogue (HGRESC), a curated resource that links microbial metabolic reactions to their associated microbial enzymes and species.

#### 2.1.1. Pre-training dataset

The largest open-source reaction dataset created by Lowe was used for pre-training of the transformer model. It was derived from the US Patent Office (USPTO) reactions, contains 1.1 M organic reactions, and was published by Lowe, DM.^32^ ^,^ ^33^ USPTO represents the out-of-domain data.

#### 2.1.2. Fine-tuning dataset

Two metabolite reaction datasets were employed to fine-tune our predictive model. The first is the MetaNetX dataset^34^ which comprises reconciled metabolites and biochemical reactions, while the second is the biotransformation reactions dataset (BRD). BRD was first collected manually from the scientific literature, including SIMMER, MetaTrans, and MicrobeFDT,^35^ and then mapped and integrated with the Unified Human Gastrointestinal Genome (UHGG) collection,^31^ which was functionally annotated using the EggNOG-mapper; yielding a list of gut reactions with their respective enzymes. Reactions in BRD were categorized into three main components (Figure 2A): Human Gut Biotransformation Reactions (HGBR), Human Biotransformation Reactions (HBR), and reactions from other environments (OBR) such as plant and soil microbiomes (all KEGG reactions). Reactions from MetaTrans lacking an external identifier were assigned an internal identifier of the form MetaN, where *N* denotes a unique sequential integer used to distinguish otherwise unannotated reactions within the dataset. BRD was processed in conjunction with MetaNetX and PP datasets to eliminate redundancy and ensure independent evaluation.

To identify duplicate reactions, SMILES were first canonicalized, and each reaction was encoded as the difference between the 2048-bit Morgan fingerprints of the products and reactants. Pairwise Tanimoto similarity was then computed across all dataset pairs. Multiple database-specific identifiers from KEGG, MetaNetX, MetaCyc, and MetaTrans corresponding to the same reaction were merged into a single integrated reaction entry concatenating the original identifiers using “_”, thereby preserving source traceability and avoiding redundancy. Subsequently, we removed only those reactions with similarity of ≥98%. This cutoff allowed us to eliminate exact and near-duplicate reactions to minimize information leakage among datasets, while retaining chemically distinct reactions and preserving the diversity necessary for robust metabolite prediction. Supplementary Figure S1 illustrates the data integration workflow used to create the fine-tuning datasets.

The integration of biotransformation reactions from other environments arises from the low accuracy yielded when training solely on HGBR reactions (more details in Section 3.2.1). BRD was used during the fine-tuning and validation phases for the construction of GUTchetp for metabolite prediction. Both BRD and MetaNetX are considered in-domain datasets because they contain metabolic reactions relevant to the prediction task. An overview of all datasets, including their versions, reaction counts, and access dates, is provided in Supplementary Table S1.

#### 2.1.3. Test datasets

To validate GUTchetp's performance, two independent test datasets, distinct from the pretraining and fine-tuning datasets, were employed. The first test dataset was used to evaluate the prediction of gut biotransformation products (PP) and comprises a list of human gut metabolite reactions derived from five databases: HGM-mediated metabolism susceptible (HGMMS) dataset,^36^ Metabolic action of gut Microbiota to Drugs (MagMD), ^37^ Metabolite Reaction Database from BioTransformer (METXBIODB),^38^ Host Gut Microbiota Metabolism Xenobiotics Database (HGMMX)^39^ and Microbiota-Active Substance Interactions database (MASI)^40^ (Figure 1A). This dataset spans six categories, including drugs, environmental chemicals, food additives, medicinal herbs, metabolites, and polyphenols (Figure 2C).

The second dataset was employed to assess enzyme prediction (EP) accuracy. We adopted the same test dataset that was previously used to evaluate three existing tools: SIMMER,^27^ DrugBug^41^, and MicrobeFDT. It consists of a list of drug metabolism events previously characterized in microbiome literature. We expanded SIMMER's dataset by adding those known enzymatic reactions from our PP dataset, with the objective of broadening enzyme coverage beyond drug-centric benchmarks.

#### 2.1.4. Gut microbial reaction–enzyme–species catalogue (HGRESC)

The UHGG genomes were functionally annotated using EggNOG-mapper,^31^ resulting in a comprehensive list of reaction–enzyme and associated species. These data were organized into the catalogue HGRESC that consists of four tables. The Taxonomy table contains the primary taxonomic classification of the human gut repertoire, including genus, family, order, class, phylum, and domain. The Reaction table provides the biochemical reactions along with their corresponding EC numbers and origin, including Gut Microbe, Gut Microbe-Human, or Human. The Enzyme table links the EC numbers with their observed frequencies across each genomic species. Finally, the Reaction-species table lists the reactions attributed to each microbial species (Figure 1A and C).

### 2.2. Module II: prediction of gut biotransformation products

#### 2.2.1. Data preprocessing

Preprocessing steps include data cleaning, data partition, data augmentation, and tokenization (Figure 1B).

##### 2.2.1.1. Data cleaning

During the data cleaning, reactions in BRD were filtered based on several criteria. First, reactant or product molecules were canonicalized and standardized using RDKit^42^ by selecting the parent molecule, removing formal charges, and then enumerating all the tautomers. Reactions with an invalid reactant or product SMILES, a reactant-product similarity below 5%, or a product share less than 10% relative to the reactant were then excluded. Afterwards, any reaction that involves molecules with more than 100 heavy atoms was removed. Finally, reactions were also discarded if either the reactant or the product had fewer than five atoms. In contrast, the MetaNetX dataset was not subjected to the same structural filtering criteria in order to preserve molecular complexity and intrinsic properties, which allowed the transformers to learn the full variability of real metabolic reactions. Reactions present in both datasets were removed to ensure that the MetaNetX reactions contain only reactions not found in the BRD dataset.

The compound-metabolite pairs in the PP test dataset were preprocessed using the above criteria, except that no restriction was placed on atom count or reactant-product similarity. PP cleaning ensures a uniform representation of molecules across methods. Supplementary Figure S2 illustrates the workflow used to preprocess and clean the BRD and PP datasets, while Supplementary Table S2 summarizes the pre-processing and post-processing (described in Section 2.2.3) settings.

##### 2.2.1.2. Data partition

The fine-tuning BRD dataset was split into 10 partitions. The partition process was carried out by a single-linkage hierarchical clustering (HC) algorithm.^43^ The general procedure of hierarchical clustering starts with *K* clusters, each containing one reaction; the most similar pair of reactions are merged into one cluster, and the previous two steps are then repeated until all similar reactions are part of a single cluster. The similarity of two reactions was measured by the Tanimoto coefficient^44^ to leverage the difference between the Morgan fingerprints (ECFP – Extended-connectivity fingerprints)^45^ of the products and the reactants. The partition of the BRD dataset into *K* clusters is denoted as follows:

Let the fine-tuning BRD dataset be: $D={\left\{\left(s_{i},t_{i}\right)\right\}}_{i=1}^{n}$ , where s_i_ represents the source sequence (reactant SMILES), t_i_ indicates the target sequence (product/metabolite SMILES), and *n* stands for the number of reactions. The clustering function *ψ* to partition the fine-tuning dataset into *K* disjoint clusters:

(1) $$D^{\left(k\right)}=\left\{\left(s_{i},t_{i}\right)\in D|\psi \;\left(s_{i},t_{i}\right)=k\right\},\;k=1,\ldots ,\;K$$

(2) $$D=\overset{K}{\underset{k=1}{\cup }}D^{\left(k\right)},\;D^{\left(j\right)}\cap D^{\left(k\right)}=\emptyset \;\mathrm{for}\;j\neq k$$

For each sub-dataset, reactants and products were stored in separate files to create input‒output pairs. Regarding USPTO, we used the same data split executed by previous research, while the MetaNetX dataset was split randomly, keeping 90% for fine-tuning and 10% for validation.

##### 2.2.1.3. Data augmentation and tokenization

The objective of this step is to increase the size of the fine-tuning datasets. We followed four strategies: (i) Data was augmented by duplicating those reactions that either contained multiple products to ensure that each reaction in the dataset had only one product (metabolite), or were considered bidirectional. Compounds such as “water”, “NADP”, “NADPH”, “proton”, “coenzyme-A”, etc. were excluded. (ii) For chemical single-representation notation, reactions were augmented *n* times by randomizing the canonical SMILES of both reactants and products. Invalid SMILES structures and reactions were ignored and not added to the datasets. (iii) For chemical multi-representation notations, only the SMILES format was randomized, as previously described. In addition to that, a special token (tag) was added at the beginning of each reactant sequence to indicate the desired metabolite chemical representation for the predictive models. These tokens were: <SM>, <CH>, and <SF> to represent SMILES, InChI, and SELFIES, respectively. The combination of different chemical representations between reactants (source) and biotransformation products (target) made each reaction augmented nine times, resulting in a total augmentation of *n × *9 times. Supplementary Table S3 reflects these combinations. (iv) An additional scenario of augmentation was also implemented, called adaptive augmentation (Adapt), where reactions were augmented *n × *2 times when low structural similarity was observed between reactants and their corresponding products; otherwise, they were augmented *n* times. SMILES strings in all sub-datasets were tokenized by atoms using the regular expression method proposed by Schwaller et al.^46^, whereas InChI and SELFIES were tokenized into their constituent symbols. During data augmentation, general atoms with a star (*) symbol were maintained for SRP and removed for MRP.

#### 2.2.2. Model construction

##### 2.2.2.1. Base model

Our approach is based on transfer learning combined with a transformer model that translates substance SMILES, InChI, or SELFIES structures into different metabolite SMILES, InChI, or SELFIES structures. We adopted the vanilla transformer proposed in.^47^ Briefly, it consists of an encoder and a decoder block, each of which is a stack of layers. A multi-head self-attention module and a position-wise feed-forward network (FFN) represent the heart of the encoder. The main task of the encoder is to first encode the input substance vector while conserving the chemical context information of the whole string sequence, and then send it to the decoder, whose principal role is to understand the chemical context and resolve the output meaningfully. The essential job of the attention layer is to memorize the large sequences of chemical representation strings and avoid network forgetting.

The pre-trained model that uses organic chemical reactions from USPTO was fine-tuned until convergence in two steps: i) fine-tuned on an in-domain (MetaNetX) dataset, and ii) mixed fine-tuned on a mix of BRD, MetaNetX, and USPTO datasets. This was repeated 10 times for each cluster (partition) generated during the pre-processing phase. The mixed fine-tuning was carried out by oversampling the in-domain datasets. We adopted the USPTO-pre-trained model from^48^ for SMILES single representation prediction, while we trained a new model using the USPTO dataset for chemical multi-representation notations.

Single representation: given a source SMILES as text $x=\left\{x_{1},...,\;x_{n}\right\}$ of length *n* tokens and a target SMILES $y=\left\{y_{1},\;...,\;y_{m}\right\}$ of length *m* tokens from dataset D, the base model aims to learn two conditional distributions, transforming *x* to *y*, by minimizing the following negative log-likelihood (cross-entropy loss):

(3) $$L_{\mathrm{nl}}=-\frac{1}{m}\overset{m}{\underset{i=1}{\sum \;}}\log\;\left(p\left(y_{i}|y_{0:i-1},x;\;\theta \right)\right)$$

where *θ* represents the model parameters and *y*_*i*_ the *i-th* token (atom) of the target metabolite.

Multi-representation: given a multi-representation dataset D spanning multiple translation directions (multi-way), consisting of a source SMILES, InChI, or SELFIES $x=\left\{x_{1},...,\;x_{n}\right\}$ of *n* tokens and a target SMILES, InChI, or SELFIES $y=\left\{y_{1},\;...,\;y_{m}\right\}$ of *m* tokens. To represent K multi-representation notations in D, a set of representation tags was introduced $R=\left\{r_{1},\;...,\;r_{K}\right\}$ , where each tag is an artificial token corresponding to a chemical representation notation in D. A tag (l_y_) is added at the beginning of *x* as a translation instruction for the transformer to indicate the chemical representation notation of *y*. Consequently, the input chemical reactant becomes $\overset{´}{x}=\left\{{l_{y},\;x}_{1},...,\;x_{n}\right\}$ and the negative log-likelihood is:

(4) $$L_{\mathrm{nl}}=-\frac{1}{m}\sum_{i=1}^{m}\;\log\left(p\;\left(y_{i}|y_{0:i-1},\overset{´}{x};\;\theta \right)\right)$$

A separate transformer model $f^{\left(k\right)}$ was trained on each cluster $D^{\left(k\right)}$ :

(5) $$f^{\left(k\right)}:S\;\to T,\;f^{\left(k\right)}\left(s\right)\approx t\;\mathrm{for}\;\left(s,t\right)\in D^{\left(k\right)}$$

During the inference phase of substance translation into biotransformation products, the base model takes the input SMILES, InChI, or SELFIES as tokens, passes them through the encoder to produce a sequence of contextual representations. The decoder then generates metabolite tokens of SMILES, InChI, or SELFIES one at a time in an autoregressive manner, leveraging the chemical context from both the substance and generated metabolite tokens, until the metabolite sequence reaches its maximum length. The generation of y_i_ can be expressed as:

(6) $$y_{i}=\mathrm{decoder}\;\left(\mathrm{encoder}\left(x|\overset{´}{x}\right),\;y<i\right)$$

During this phase, the beam search strategy is also employed to explore multiple possible translated metabolites or hypotheses for a given substance and select the best one. Beam search improves the overall accuracy of the metabolite predictions produced by our tool.^49^

##### 2.2.2.2. Ensemble model

Two methods of ensemble learning were applied:

(i) During the fine-tuning process, a specified number of model states (checkpoints) are produced after a certain number of steps. Once the cross-entropy loss reaches a minimum, indicating a risk of overfitting if fine-tuning continues, the process is paused, and the model is evaluated on the validation dataset. A checkpoint ensemble is applied, averaging the predictions from the “checkpoints” of the best model states within a single training process with the objective of improving transformer performance. Formally, given a parameterized transformer $f_{\theta_{t}}$ model, where $\theta_{t}$ denotes the weights at training step t, a checkpoint C_i_ corresponds to $\theta_{t_{i}}$ with $t_{i}\in t_{1},t_{2},\ldots ,t_{n}$ . Thus, a collection of candidate models after *n* such iterations was obtained { $C_{1},C_{2},\ldots ,C_{n}\}$ , with the best candidate C_x_ at step *x*. Subsequently, to identify the most effective configuration, a selection procedure based on an ensemble model of a subset of candidates $S\subseteq C_{1},\ldots ,C_{n}$ was applied. This subset always includes the best candidate Cx. Candidate subsets were evaluated on the test dataset (PP) using the BLEU metric (See Section 2.4).
  Fine-tuning under different model configurations generated a pool of checkpoints. To identify an optimal subset of model checkpoints that maximizes the coverage of correctly predicted compounds in the PP dataset, we implemented a brute-force algorithm. First, checkpoint models were ranked according to the number of correct predictions they provide. Each checkpoint is then considered only if it contributes at least one compound not already covered in the set of selected compounds, and the overall accuracy is updated as the fraction of unique compounds covered in the test dataset. Finally, the final optimal subset of checkpoints is defined as the one that achieves the highest accuracy during this iterative search process. This search algorithm is formalized in pseudocode as shown in the Supplementary Table S4.
(ii) A majority voting ensemble was applied to combine the decisions of K base models with the objective of selecting the best metabolite string structures that best fit the input substances. The majority voting across *n*-best metabolites or hypotheses was employed and given by:
(7) $$\hat{h}=\arg\max_{h\in H}\mathrm{count}\left(h\right)$$
  where *n* is the number of hypotheses per base model, $H=\left\{h_{i,j}|i=1,\ldots ,\mathrm{K;}\;j=1,\ldots ,n\right\}$ and *count(h)* denotes the number of times hypothesis *h* appears in *H*.

##### 2.2.2.3. Model selection agent

This step aims to select the best *N* partitions, called the winners, which help identify those models that were fine-tuned or mixed fine-tuned using these winning partitions. The selected models thus form the core of the ensemble learning approach. The agent helps to reduce computational time and maintain GUTchetp’s performance for predicting gut biotransformation products. Two steps were carried out to build it. Firstly, a forest of trees based on fixed-length Morgan fingerprints^45^ was built, where each reactant/substance was projected into a vector space and associated with one or more partitions. These fingerprints were organized into a forest of trees (Figure 1B). Secondly, for a new query compound, the same fingerprint was computed and propagated through the forest to retrieve associated partitions using similarity-weighted scoring. Finally, models that were fine-tuned on the selected partitions are added to the Base level (top-ten base models). The two steps of the model selection agent are formalized in pseudocode as shown in the Supplementary Table S5.

#### 2.2.3. Prediction postprocessing

The predicted SMILES candidates were canonicalized and subsequently filtered according to three criteria. First, SMILES that could not be successfully parsed by RDkit and therefore violated the syntax rules of the SMILES language were removed. Second, candidates containing molecules with more than 100 heavy atoms were discarded. Lastly, candidates were removed if they contained atoms not present in the parent molecule and not among the common organic elements (C, O, H, S, P, N). Predicted SELFIES and InChI candidates were first converted into SMILES and the same previous three rules were then applied.

#### 2.2.4. Candidate reranking

The predicted SMILES candidates were scored and ranked using the method employed by.^20^ This method takes into consideration both the importance of the candidates' ranks and the number of times a candidate is predicted across different augmented molecules. This is expressed by Equation (8):

(8) $$\mathrm{score}=\sum_{n=1}^{\mathrm{Augmentation}\;}\sum_{i=k}^{\mathrm{Top}-K}\frac{1}{1+\alpha \;*\;\left(k-1\right)}$$

where *α* is the weighting parameter. A higher value of *α* means the ranking given by the log-likelihood is more important and the frequency of candidates that appear consistently across augmentations is less important, and vice versa. When the SMILES candidate is invalid, the second summation is equal to zero.

The same postprocessing, filtering criteria, and re-ranking were also applied to all predicted candidates across benchmarked methods (Supplementary Table S2).

### 2.3. Module III: identification of enzymes and gut microbes involved in compound transformation

The main objective of this module is to search the HGBR dataset for the most similar reactions using the predicted one (the queried compound → predicted gut biotransformation product). Then, it links these reactions to the corresponding microbial repertoire that was extracted from the UHGG dataset and literature. This process involves three steps (Figure 1C): construction of a gut reactions' similarity matrix, re-ranking, and retrieval of similar reactions and enzymes utilizing HGRESC.

#### 2.3.1. Similarity matrix construction

We adopted the methodology used by the SIMMER tool, with one key modification. The process consisted of two main sub-steps. In the first sub-step, reaction fingerprints were determined by calculating the difference between the Atom-Pair (AP) fingerprints of the products and reactants.^27^ In the second sub-step, a pairwise similarity matrix was computed using Tanimoto coefficients to measure the structural similarity between each reaction in the HGBR dataset. The key modification we introduced was the expansion of reactions with multiple products into separate, and individual reactions.

#### 2.3.2. Re-ranker construction

The reaction re-ranker was developed using two random forest (RF) classifiers trained to predict the first- and second-level EC numbers, respectively. The third level was omitted experimentally (See Section 3.3). The adoption of a single-label classifier is due to the small proportion of reactions associated with multiple EC numbers at both first and second levels (more details in Section 3.1). The re-ranker development process consisted of two sub-steps. Using the CLAIRE training and testing datasets^28^, we built RF classifiers in the first sub-step to assess every possible combination of five molecular fingerprints, where each reaction fingerprint was calculated by subtracting the 1024-bit product fingerprint vector from the corresponding 1024-bit reactant fingerprint vector. As a result, we were able to identify the best fingerprint combination. These five fingerprints are: Atom-Pair (AP), Morgan (M), RDKit, Pattern (*P*), and Topological Torsion (TT).^50^ The concatenated fingerprint vectors were reduced by performing an element-wise summation across the consecutive 1024-dimensional feature blocks, yielding a single 1024-dimensional fingerprint vector. This aggregation decreased the feature dimensionality, thereby lowering the computational cost and memory requirements during both model training and prediction while maintaining a rich molecular representation.

In the second sub-step, the optimal molecular fingerprint combinations were used to train two random forest models (RF1 and RF2) for hierarchical EC number classification using five-fold cross-validation. Prediction confidence was quantified using the prediction margin, defined as the difference between the two highest class probabilities.^51^ A larger margin indicates a greater confidence in the predicted class relative to the closest competing class. Confidence tiers were determined empirically from out-of-fold (OOF) predictions by analyzing the relationship between Top-1/Top-2 prediction score margin and prediction accuracy. Margin confidence value close to 1 indicates a confident classification, while values near 0 correspond to high predictive uncertainty. Confidence thresholds were selected to categorize predictions as High-confidence, Suggestive, or Low-confidence. Rather than relying on predefined accuracies to determine these thresholds, they were automatically determined by evaluating accuracy pairs. For each candidate, the lowest margin threshold empirically achieving the target accuracy was identified to maximize reaction coverage. The final models and the confidence thresholds were subsequently used to predict unseen reactions in the EP dataset.

#### 2.3.3. Retrieval of similar reactions, enzymes, and species

Given a set of predicted reactions resulting from a queried compound and the predicted products by the ensemble model (first component of GUTchetp), we first generated reaction fingerprints and then compared them to the precomputed similarity matrix by calculating Tanimoto coefficients between each queried fingerprint and all reactions in the HGBR dataset. Afterwards, the new resulting similarity vectors were used to rank the referenced reactions based on their Euclidean distance from the query fingerprint.^27^ Smaller distances indicate greater overall similarity. Candidates were re-ranked by computing a score that integrates the prediction consistency of enzyme classifiers (first-level and second-level) with chemical similarity. The scoring equation is given by Equation (9):

(9) $$S_{r}=C_{\mathrm{EC}}\left(r\right)\;.\;S_{\mathrm{chem}}\left(r\right)$$

where $C_{\mathrm{EC}}\;(r)$ denotes the enzymatic consistency score, reflecting how well the candidate reaction’s EC number matches the enzyme predictions, while $S_{\mathrm{chem}}(r)$ refers to the chemical similarity score. The enzymatic consistency score is calculated as a weighted sum of matches at each EC level.

(10) $$C_{\mathrm{EC}}\left(r\right)=W_{1}\cdot \left({\mathrm{ec}}_{r,\;1}={\hat{\mathrm{ec}}}_{1}\right)+W_{2}\cdot \left({\mathrm{ec}}_{r,\;1}={\hat{\mathrm{ec}}}_{1}\wedge {\mathrm{ec}}_{r,\;2}={\hat{\mathrm{ec}}}_{2}\right)$$

where ${\mathrm{ec}}_{r,k}$ refers to the *k*-th level of the candidate reaction’s EC number and ${\hat{\mathrm{ec}}}_{k}$ is the predicted EC label at level *k*. The weights W1 and W2 correspond to correct predictions at first- and second-level EC numbers, respectively, and were determined experimentally (See Section 3.3). The sum of both weights is equal to one.

Finally, HGRESC is queried using either the predicted EC number to retrieve the gut enzymatic repertoire associated with the compound under study.

### 2.4. Evaluation metrics

Five evaluation metrics were employed to assess the performance of our proposed GUTchetp approach, namely Top-N accuracy, Top-N BLEU (BiLingual Evaluation Understudy),^52^ Top-N MaxFrag (Maximal Fragment accuracy), confidence score for the translation task, and weighted average F1-score. Predicted metabolites were considered correct when they exhibited either an exact topological fingerprint match or an exact structural fingerprint match with the ground truth, and these matches were used to compute BLEU, accuracy, and MaxFrag accuracy.

Top-N accuracy (Acc) represents the percentage of chemical compounds in the test dataset where the ground truth is included among the Top-N predicted candidates.

BLEU is a metric that compares a predicted metabolite string to one or more reference biotransformation product strings by quantifying the overlap *n*-atoms (tokens). Metabolite prediction is treated as a sequence-to-sequence translation task, analogous to natural language translation, in which SMILES, InChI, or SELFIES representations are compared. In this context, BLEU measures the similarity between the GUTchetp translation and reference biotransformation products reported in the literature for the same source compound. BLEU scores range from zero (poor translation) to one (perfect translation). Accordingly, Top-N BLEU measures the similarity between the reference metabolite and the Top-N predicted metabolites, capturing how well the model ranks plausible chemical outputs.

Top-N MaxFrag accuracy is an extension of the conventional Top-N accuracy metric, originally introduced in retrosynthesis prediction.^53^ MaxFrag compares the largest molecular fragment of each predicted metabolite with that of the reference metabolite. This metric is particularly relevant because many biotransformation reactions preserve the principal molecular scaffold while altering only localized regions.

BLEU and MaxFrag accuracy provide complementary evaluations of prediction quality and performance. BLEU measures global sequence-level similarity, whereas MaxFrag evaluates the correctness of the core molecular structural. Together, they provide a more informative assessment of gut biotransformation product prediction performance.

Confidence score refers to the exponential of the average log-likelihood assigned by the neural translation model to each atom (token) in the predicted biotransformation product. A higher average atom probability indicates greater model confidence in its prediction. This metric only reflects the transformer's internal certainty in predicting the atom sequence of the biotransformation product, but it does not directly measure the quality of the compound-to-metabolite translation, which is measured by the BLEU metric.

The weighted average F1-score (WAF1) was used to evaluate the performance of enzyme class classifiers. WAF1 calculates the F1-score independently for each class and then averages them based on the number of true predictions per class. The metric mitigates the class imbalance problem by ensuring that each class equally influences the overall performance metric.^28^

### 2.5. Statistical significance testing

To assess whether the observed performance differences between any two models (A and B) are statistically significant, we employed two complementary non-parametric tests selected according to the nature of each performance metric. For continuous values of BLEU scores, Wilcoxon signed-rank test (one-tailed) was applied, reporting the test statistic (W), the one-tailed *p*-value in the direction of the new model (B) (*p*(B > A), indicating B outperforms A, and the Rank-Biserial Correlation (RBC) as the corresponding effect size. For binary values of accuracy and MaxFrag accuracy, McNemar's test was applied, reporting the one-tailed *p*-value in the direction of the new model (B) (*p*(B > A), and the Odd Ratio (OR) as the effect size. In both tests, statistical significance was determined using a predefined *p*-value threshold (*p* < 0.05 >, and effect sizes were interpreted using standard thresholds for their respective measures. For both metrics, the lower and upper bounds of the 95% confidence interval (95% CI) for the mean are also reported.

## 3. Results

### 3.1. Data exploration and chemical space analysis of the fine-tuning and test datasets

The two primary challenges in building GUTchetp were: (1) the limited size and heterogeneity of gut biotransformation reaction datasets available in the literature; and (2) the complexity and poor understanding of the extremely high-dimensional chemical reaction space in the gut, which is crucial for accurate metabolic modeling and microbiome-based reaction prediction. Therefore, we first targeted the UHGG collection,^31^a complete bacterial repertoire of the human gut microbiota, encompassing 204,938 non-redundant genomes. This collection consists of species (SC) and unified (UC) catalogues, covering 4644 gut prokaryote species (4616 Bacteria and 28 Archaea) with their respective core functional repertoire that spans 2904 enzymes, 3686 reactions, more than 20,000 genes, 704 modules, 422 pathways, and 9239 orthologies. Subsequently, we mapped the obtained UHGG data to several biotransformation reaction datasets published up to the end of January 2025, including SIMMER, MetaTrans, and MicrobeFDT.

The integrated and cleaned data was used for fine-tuning and validation of GUTchetp and is hereafter referred to as the Biotransformation Reaction Dataset (BRD). BRD contains a total of 25,393 metabolite reactions. The distribution of these reactions across the source datasets before and after the comprehensive cleaning process (Section 2.1.2) is shown in Supplementary Figure S3. The final dataset is primarily composed of reactions originating from MetaTrans (9382 reactions), SIMMER (5229), MicrobeFDT (3303), and UHGG (2824). BRD reactions were linked to one or more reference datasets, resulting in 7918 KEGG, 592 MetaNetX, and 101 MetaCyc annotations (provided by the SIMMER dataset).

BRD was categorized into three main categories (Figure 2A): Human Gut Biotransformation Reactions (HGBR), comprising 26.50% of the dataset; Human Biotransformation Reactions (HBR), representing 1.60%; and reactions from other environments (OBR), accounting for the remaining 71.90%. In total, BRD consists of 25,393 unique reactions catalyzed by 4,435 distinct enzymes, consisting of 57.83% enzymatic and 42.17% non-enzymatic reactions. Among enzymatic reactions, oxidoreductases, transferases, and hydrolases constitute the most represented enzyme classes, accounting for 35.50%, 28.90%, and 18.30%, respectively (Figure 2B). Notably, only a small fraction of reactions are associated with multiple EC numbers (Supplementary Figure S4 (A)), with proportions increasing from 1.32% (336 reactions) at the first EC level to 1.40% (256) at the second level, 1.52% (387) at the third level, and 2.54% (646) at the fourth level. Within this subset, HGBR-associated reactions (GutMicrobes and GutMicrobes & Human) account for 0.19%, 0.27%, 0.30%, and 0.57% across EC levels 1–4, respectively (Supplementary Figure S4 (B)).

The BRD was split into 10 partitions using hierarchical clustering (HC) with the single linkage method and a minimum inter-cluster distance of 0.3 to ensure that reactions within a partition were more similar to each other than to those in other partitions (folds). For each iteration, nine of these partitions were combined to form the fine-tuning dataset, while the remaining partition (fold) was used as the validation dataset. This process was repeated 10 times, each time a different partition was used for validation, which resulted in 10 distinct fine-tuning-validation splits to train 10 base models, each capturing a unique perspective on the gut reaction data. The number of reactions in each validation and fine-tuning dataset was approximately 2540 and 22,854 reactions, respectively. Accordingly, metabolite reactions from BRD and MetaNetX (25,125), respectively, were used in two separate rounds to fine-tune and mixed-fine-tune GUTchetp. The MetaNetX dataset was partitioned randomly into 10% for validation and 90% for fine-tuning.

The GUTchetp component responsible for predicting gut biotransformation products was evaluated on the PP dataset, which consists of 348 chemical compounds associated with 479 experimentally verified metabolites across six distinct categories. More specifically, the largest proportion corresponds to polyphenols (29.00%), followed by drugs (27.80%), biotransformation products (metabolites) (22.30%), food additives (8.60%), medicinal herbs (7.50%), and environmental chemicals (4.80%). Figure 2C illustrates the distribution of the PP test dataset across compound categories.

To assess the chemical diversity of the fine-tuning BRD, MetaNetX, and PP datasets, we performed t-Distributed Stochastic Neighbor Embedding (t-SNE)^54^ on 2,048-bit Morgan fingerprints generated separately from each dataset. Figure 2D depicts the distribution of biotransformation reactions from BRD colored according to their environmental origin. It can be noted that the t-SNE plot reveals substantial overlap among all environments, indicating a shared chemical space across diverse biological contexts. Notably, gut (orange) and gut-human (yellow) biotransformation reactions were clustered densely near the center, which means that they share a chemically central region that might reflect a biologically conserved space. Conversely, reactions derived from other environments were more broadly dispersed toward the periphery, reflecting greater structural or mechanistic divergence. On the other hand, Figure 2E and F show substantial overlap between the PP dataset (orange) and the fine-tuning datasets (BRD and MetaNetX; blue), indicating minimal distribution shift between them, and reflecting shared coverage of the underlying chemical space rather than duplication of specific reactions. This supports the validity of the fine-tuning-test split and the generalizability of the transformers.

Additionally, we conducted empirical checks beyond visualization to ensure that no dataset contamination occurred between fine-tuning and testing datasets. First, we performed a Principal Components Analysis (PCA) to examine the spatial distribution of highly similar reactions (≥95% similarity) (Supplementary Figure S4 (C)). The results showed that only two reactions from the PP dataset were highly similar to one reaction from fine-tuning datasets and one additional reaction from the PP dataset, respectively. However, despite this apparent overlap, the reactions differed substantially in the underlying chemistry. Supplementary Table S6 provides a detailed comparison of these cases. For instance, the biotransformations of ginsenoside Rb1 and Rc into compound K exhibit strong similarity in reaction space, as both map to the same product. However, they differ substantially in substrate structure and enzymatic requirements. Rb1 contains a relatively simpler glycosidic architecture, whereas Rc includes an additional rhamnose-glycosidic architecture. Consequently, the two transformations are mediated by partially different microbial taxa.^55^ Second, we conducted a pairwise Tanimoto similarity analysis, which revealed similarity values below 4% across datasets (Figure 2G and Supplementary Figure S4 (D)), indicating minimal structural overlap. This confirms once more that the apparent similarity reflects broad category-level proximity rather than duplication or leakage between datasets. despite some visual overlap in chemical space, the test dataset introduces significant chemical diversity compared to the fine-tuning datasets.

Moreover, Figure 2H−N present a comparison of molecular properties across the four aforementioned datasets. These properties include reactant-product similarity, transformation ratios, molecular mass changes, reactant SMILES lengths, number of atoms, structural transformation types (chirality, ring opening, ring formation), and functional groups. By scrutinizing these properties, we aim to identify potential discrepancies, biases, or anomalies that could influence the prediction of gut biotransformation products (Figure 7A−F). The density plots reveal notable differences; For instance, the test dataset (PP) shows multiple peaks at higher similarity values (Figure 2H), reflecting a closer resemblance between reactant and product, while the transformation ratio (Figure 2I) indicates that the reactions within MetaNetX exhibit a wider variation in transformation ratios compared to the other datasets. In further analysis of Figure 2J−L, we found that the HBR & OBR and HGBR datasets predominantly exhibit smaller mass changes, shorter SMILES lengths, and lower reactant atom counts, indicating slight metabolic transformations. In contrast, the PP dataset uniquely exhibits larger mass variations, distinct SMILES length peaks, and molecules with varying reactant atom counts that reflect diverse molecular properties and chemical transformation complexity. Turning to Figure 2M, we note the strong representation of chirality and ring-forming reactions in the PP dataset, whereas ring-opening reactions occur more frequently in HGBR. Additionally, Figure 2N highlights that the PP dataset contains a notably broader and more heterogeneous mix of functional groups when compared to the other datasets. The PP and HGBR datasets exhibit broadly similar patterns, indicating a comparable structural and chemical diversity. However, some descriptors exhibit differences, such as rotatable bonds, stereocenters, and aromatic and aliphatic ring counts, which indicate variations in molecular properties between the two datasets, as shown in Supplementary Figure S5. This rich diversity of the PP dataset confirms that our experiments encompass a wide spectrum of chemical behaviors and thereby increase its effectiveness as a benchmark for predictive models’ robustness across varied chemical contexts.

On the other hand, the GUTchetp component responsible for predicting gut microbial enzymes and species was evaluated using the EP dataset, which consists of 95 compound metabolism events (gut microbial biotransformation profiles) catalyzed by enzymes that span five EC classes. As shown in Supplementary Figure S6 (A), oxidoreductases dominate the dataset (40 reactions, 42.10%), followed by transferases (30 reactions, 31.60%), hydrolases (18 reactions, 18.90%), lyases (5 reactions, 5.30%), and isomerases (2 reactions, 1.60%). The pie chart in Supplementary Figure S6 (B) summarizes the distribution of enzymatic reactions at the second-level EC classification. EC:2.4 and EC:1.14 account for the largest fractions, representing 25.30% and 23.20% of reactions, respectively. Substantial contributions are also observed for EC:3.2 and EC:1.7, whereas most other EC subclasses each represent less than 5% of the total reactions. Supplementary Table S7, presents a summary of datasets used for training, fine-tuning, validation, and testing of the GUTchetp framework.

Finally, gut microbial species represented in the HGRESC account for 71.13% of all unique enzyme activities identified in the BRD. This coverage is broadly distributed across seven first-level EC classes, ranging from 62.07% for isomerases to 80.36 for hydrolases, demonstrating the extensive representation of gut microbial enzymatic functions within the HGRESC. Consequently, this broad coverage enhances the biological interpretation of prediction results robustly for the majority of compounds, as most predicted biotransformations can be associated with characterized enzymatic functions. However, predictions involving enzymatic activities absent from HGRESC may exhibit reduced mechanistic interpretability. Supplementary Figure S7 (A) presents the overall coverage and missing rates, whereas Supplementary Figures S7 (B-H) show the corresponding coverage-missing values across the seven enzyme main classes.

### 3.2. Evaluation of gut biotransformation product prediction

We conducted an ablation study to examine how each base model fine-tuning component affects performance. Specifically, we investigated the effect of data size, adaptive augmentation, checkpoint averaging, fine-tuning versus mixed fine-tuning, data partitions, and single- versus multi-representation notations. These components were combined in different ways to create 12 distinct model configurations. In total, 120 models (12 configurations × 10 partitions) were fine-tuned, evaluated, and tested to identify the optimal ensemble model configuration for accurately predicting gut biotransformation products. This ensemble forms the core of the first GUTchetp component. All models were fine-tuned using a standard base transformer with six encoder and decoder layers and eight attention heads. Further details about the transformer architecture design, fine-tuning, and translation parameters can be found in Supplementary Table S9. The beam size and *n*-best were set to 20 for single models and 200 for ensemble models. The choice of 200 for ensemble models was motivated by the high diversity of species in the human gut microbiome used in our experiments, which allows the model to account for low-abundance species that may contribute to rare but biologically relevant biotransformation products. MaxFrag accuracy results are plotted in Supplementary Figure S8 (A−E).

#### 3.2.1. Baseline model and impact of data size

The pre-trained USPTO model was initially fine-tuned using only the human gut biotransformation reaction (HGBR) dataset, but this yielded trivial performance improvement (only 0.06% at Top-20). Therefore, the HGBR dataset was extended by integrating additional biotransformation reactions from the human (HBR) and other environments (OBR) datasets. Figure 3A shows that model performance improves as the size and chemical diversity of the biotransformation dataset increase, indicating that the model benefits from broader chemical context during training. Remarkably, when GUTchetp was fine-tuned on the BRD dataset, it outperformed models fine-tuned exclusively on the HGBR dataset by 14.78%, 22.94%, 24.05%, 22.87%, and 22.91% in the Top-1, Top-5, Top-10, Top-15, and Top-20 BLEU scores, respectively. A similar trend was observed for MaxFrag accuracy, which achieved improvements of 14.83%, 23.59%, 24.22%, 22.76%, and 22.34% for the corresponding Top-N (Supplementary Figure S8 (A)). We consider the fine-tuned model on the HGBR dataset as the baseline model, which was subsequently compared later with our proposed approach to evaluate improvements in gut biotransformation product prediction performance (See Sections 3.2.6).

#### 3.2.2. Impact of adaptive data augmentation

Figure 3B illustrates the effectiveness of adaptive data augmentation compared to a traditional uniform augmentation scenario, where all reactions are augmented equally. This was carried out by augmenting *n* × 2 times those reactions in which the similarity between the reactants and the product is less or equal to 25%; otherwise, reactions were augmented only *n* times. It can be noted that Adapt5 and Adapt10 models generally perform better than their counterparts A5 and A10. At Top-20, Adapt10 achieved the highest performance, with BLEU score improvements of approximately 1.84%, 1.02%, and 1.17%, and MaxFrag accuracy gains of 1.46%, 0.21%, and 0.41% (Supplementary Figure S8 (B)), relative to A5, Adapt5, and A10, respectively. In contrast, no performance gains at Top-1 for either BLEU or MaxFrag accuracy, where A10 performed slightly better than A5.

To highlight the applicability of these enhancements in challenging prediction scenarios, we consider Sudan III as a representative case where GUTchetp successfully predicted the correct gut metabolite that was not captured by alternative approaches (MetaPredictor and MetaTrans). Despite sharing only 9% structural similarity with its gut metabolite 1,4-phenylenediamine, GUTchetp accurately predicted this metabolite 72 times with a structural confidence of 83%, ranking it among the top four candidates.

#### 3.2.3. Fine-tuning vs. mixed fine-tuning: Impact of two steps in transfer learning

The pre-trained model USPTO was first fine-tuned on the MetaNetX dataset and then mixed-fine-tuned by oversampling the mixed data until convergence. The oversampling proportions were 70% from BRD, 20% from MetaNetX, and 10% from USPTO. As illustrated in Figure 3C, the cross-entropy loss of the best MFT checkpoint is lower (0.27) compared to the standard fine-tuning approach (0.32). This indicates that models fine-tuned using the MFT strategy make more confident predictions with fewer errors for gut microbial biotransformation products. Figure 3D further supports this observation by showing consistent gains in prediction quality across the Top-N predicted metabolites. Compared with the None-MFT model, the MFT model improved BLEU scores by 2.95%, 1.97%, 2.04%, 2.20%, and 3.20% and MaxFrag accuracy by 3.34%, 1.88%, 2.08%, 2.09%, and 2.92% (Supplementary Figure S8 (C)) at Top-1, Top-5, Top-10, Top-15, and Top-20, respectively.

Furthermore, the heatmap in Supplementary Figure S9 compares the absolute differences in BLEU scores between 60 models (6 configurations × 10 partitions; See Section 3.2.7 and Supplementary Tables S10 and S11) fine-tuned exclusively on BRD (one-step) and 60 mixed-fine-tuned (two-step) models. Generally, two-step fine-tunings leads to improved or comparable BLEU scores compared to one-step fine-tuning. For instance, for Top-15 and 20 predictions using Partition 4 along with the configurations A5 and A5MFT, BLEU scores improved by 6.62% and 5.97%, respectively. On the other hand, other configurations suffered noticeable declines (e.g., Partitions 3, Adapt5MRP, Top-1: −4.09% BLEU), specifically, models fine-tuned with chemical multi-representation. In addition, we note that the chemical diversity within each data partition plays a critical role in the fine-tuning efficacy. For instance, Partitions 4 and 6 consistently showed large positive gains (dark purple) from two-step fine-tuning, while Partitions 7 and 8 often showed negative or minimal gains (burnt orange). Supplementary Figure S10 details the BLEU scores of all 120 base models. Among the twelve configurations, Adapt10MFT achieved the best performance, reaching BLEU scores of 27.18% (Partition 10) and 62.05% (Partition 1) at Top-1 and Top-20 predictions, respectively. Consistent with these findings, Supplementary Figure S11 shows that the same model also achieved the highest MaxFrag accuracy, with values of 26.93% (Partition 7) and 61.59% (Partition 10) for the corresponding Top-1 and Top-20 predictions, respectively. Notably, once again, configurations incorporating mixed fine-tuning (MFT) consistently outperformed their None-MFT counterparts across all Top-N values.

#### 3.2.4. Impact of checkpoint ensembles

Subsequently, we investigated the impact of checkpoint averaging to gain insight into how it improves the prediction of gut biotransformation products. Through our experiments employing model M1 (Supplementary Table S11), we observed a clear advantage of checkpoint averaging over the use of single and non-averaged checkpoints. As shown in Figure 3E, the Top-20 BLEU score increased from 60.18% without averaging to 61.98% with ensemble modeling that averaged all the checkpoints generated during the fine-tuning iterations from steps 10 to 90. This result suggests that the ensemble of checkpoints helped to capture a more comprehensive representation of the underlying chemical biotransformation patterns by integrating knowledge learned at various steps of the fine-tuning process, thereby reducing overfitting and improving performance on the test dataset. Moreover, the ensemble of checkpoints from steps 30–60 correctly predicted four new gut biotransformation products that were not identified by the single checkpoints during the same interval.

#### 3.2.5. Impact of multi-representation prediction

To assess the effectiveness of using MNMT to predict gut biotransformation products, we conducted evaluations on several translation tasks: SM2SM, SM2CH, SM2SF, CH2SM, CH2CH, CH2SF, SF2SM, SF2CH, and SF2SF. Figure 3F and Supplementary Figure S8 (D) illustrate that GUTchetp showed moderate performance for the tasks SF2SM and SF2CH, while the tasks SF2SF, SM2SF, and CH2SF consistently achieved higher scores at Top-10, Top-15, and Top-20, suggesting that translations involving the chemical SELFIES representation benefit from its structural robustness. The best translation task at Top-1 and Top-5 was achieved by SM2SM. However, when the number of candidate outputs increased to Top-10, the BLEU score and MaxFrag accuracy decreased, in contrast to the other tasks that continued to show improvement.

Lactulose exemplifies the advantages of using MNMT in predicting gut biotransformation products. Intestinal bacteria such as *Lactobacillus*, *Bacteroides,* and *E. coli* convert lactulose into fructose and galactose.^56^ All 120 base models correctly predicted galactose, but only models that used the MRP strategy successfully predicted fructose using SM2SM, SM2CH, SF2CH, and CH2CH tasks.

The attention patterns in Figure 3G-J show how the model maps atom tokens of lactulose in SMILES, InChI, or SELFIES to their corresponding fructose tokens. Layer 4 of the attention mechanism captures the chemical context across multiple molecular representations. For instance, the red box in Figure 3G highlights a contiguous set of SELFIES tokens, “[O][C][Branch1][Ring1]” that are highly correlated with the InChI substring “(8)1-12-6/”. This indicates that the model can translate ring-closure semantics into explicit atom-to-atom connectivity. Other tokens, such as “[#Branch2]” and “[Ring1]” are not mapped to a single InChI fragment, but instead exhibit distributed attention across multiple InChI tokens, forming bright vertical bands in the heatmap. Such poly-semantic attention reflects how the model leverages the global molecular context rather than isolated token-to-token mapping during translation. Chemically, these mappings match known gut microbial biotransformation: glycosidic cleavage is followed by ring opening, converting cyclic sugar motifs from lactulose into linear fructose while maintaining the anomeric carbon‒oxygen framework.

The SMILES fragment “C(C(C(” is mapped with the InChI segment “-6(11)5(10)4(9)3(8” (Figure 3H), reflecting the ability of the model to translate the hierarchical branching syntax into explicit atom numbering and substituent positions. We also observed that the region enclosed by the red box in Figure 3I represents the translation of the lactulose fragment “(16)7(17)8(18)11(21-4)22-9” into the fructose fragment “5(10)4(9)3(8)1-12-6/”. This process illustrates how glycosidic bonds are cleaved and the molecule reorganized into a cyclic fructose structure. Thus, the model effectively removes the inter-sugar linkage while preserving the internal hydroxylated carbon framework, allowing it to capture the main structural transformation from disaccharide to monosaccharide during gut microbial hydrolysis.

Finally, Figure 3J highlights intense attention across multiple rows. This is likely associated with the closing parenthesis or ring-closure digit in the SMILES strings, which is essential for predicting the fructose ring structure. The red box in the figure shows how the lactulose fragment “C1(C(” is translated into the fructose segment “(O)C(CO1)O”, reflecting the reorganization of the fructofuranose ring scaffold following cleavage of inter-saccharide linkages. Supplementary Figures S12 (A−D) further illustrate the contribution of individual lactulose tokens, which made the transformer focus on the most relevant tokens of the input when generating the translation into fructose.

#### 3.2.6. Ensemble learning

To improve the quality of gut biotransformation product predictions, we first combined the predictions of base models for each data partition and configuration using a majority voting approach, obtaining 12 ensemble models (Figure 3K and Supplementary Figure S8 (E)). It is essential to highlight that these models were derived by averaging checkpoints generated during fine-tuning iterations, where each one includes the checkpoint with the highest BLEU score.

The Adapt10MFT configuration achieved the best overall performance, attaining the highest BLEU scores of 50.6% and 58.42% at Top-5 and Top-10, respectively, while also achieving the highest MaxFrag accuracy of 49.69% and 57.83% (Supplementary Figure S8 (E)). These findings further support that the optimal configuration for molecular translation tasks was achieved by combining adaptive augmentation with mixed fine-tuning. Moreover, the ensemble A10MFT model performed even better, reaching up to 63.80% at Top-20, outperforming the best base model (Supplementary Figure S10) by 1.75%, and up to 71.44% at Top-100. Interestingly, across all augmentation strategies (A5MRP_MFT, Adapt5MRP, Adapt10MRP, and Adapt10MRP_MFT), ensemble models fine-tuned using molecular multi-representation consistently showed lower BLEU scores and MaxFrag accuracy than their SMILES-only counterparts.

To determine whether these performance differences were statistically meaningful, pairwise comparisons were performed across main configurations. First, relative to the HGBR baseline, A5 achieved significantly higher BLEU scores across all prediction levels, as shown in Supplementary 13(A), with a Top-1 95% CI = 21.17%–28.91%, W = 1049, *p* = 4.24 × 10^−12^, and |RBC|=0.67. A5 also achieved significantly higher MaxFrag accuracy, as shown in Supplementary Figure S13(B), with a Top-1 95% CI = 20.56%–28.29%, Statistic = 18, *p* = 2.97 × 10^−12^, and OR = 0.20. The predominantly medium-to-large effect sizes indicate that incorporating reactions from other environments enhanced the model's ability to predict gut biotransformation products.

Second, and in contrast, adaptive augmentation did not provide additional benefits. No statistically significant improvements were observed for Adapt5 over A5 in either BLEU scores (Supplementary Figure S14 (A)) or MaxFrag accuracy (Supplementary Figure S14 (B)), nor for Adapt10 over A10 in BLEU scores (Supplementary Figure S14 (C)) or MaxFrag accuracy (Supplementary Figure S14 (D)). Effect sizes remained small across the Top-N ranges.

Third, the impact of mixed fine-tuning using SMILES was dependent on the underlying fine-tuning configuration, and the pattern of statistical significance differed markedly between the two evaluation metrics. For A5MFT versus A5, significant improvements in BLEU were observed at Top-1 (95% CI = 24.23%–32.29%, W = 560.50, *p* = 2.45 × 10^−2^, |RBC|=0.27) and across Top-N ranks below 39, with small-to-medium effect sizes as shown in Supplementary Figure S15 (A). While the corresponding MaxFrag accuracy revealed a significant improvement only at Top-1 with 95% CI of 23.74%–31.79%, W = 19, *p* = 4.02 × 10^−2^, and |RBC|=0.54 (Supplementary Figure S15 (B)). Additionally, Supplementary Figures S15 (C) and (G) show that mixed fine-tuning provided additional benefits when extensive data augmentation was applied. A10MFT demonstrated the strongest overall significance (Supplementary Figure S15 (C)), reaching *p* < 0.001 (minimum *p* = 8.02 × 10^−4^, maximum |RBC| = 0.53) for BLEU scores and *p* < 0.01 (minimum *p* = 7.63 × 10^−3^, maximum |RBC| = 0.39) for MaxFrag accuracy across multiple levels with medium-to-large effect sizes (Supplementary Figure S15 (D)). The MaxFrag accuracy exhibited an absence of significant regions or substantially attenuated significance across those same level intervals. Relative to the adaptive augmentation-mixed finetuning configuration, both Adapt5MFT and Adapt10MFT showed statistically significant improvements in BLEU over their respective non-MFT counterparts, with small-to-medium effect sizes at intermediate Top-N levels (minimum *p* = 1.10 × 10^−2^ and maximum |RBC| = 0.36) for Adapt5, and predominantly medium effects across several ranks for Adapt10 (minimum *p* = 6.97 × 10^−3^ and maximum |RBC| = 0.43). In contrast, statistically significant improvements in MaxFrag accuracy were limited to only Top-39 for Adapt5MFT (Supplementary Figure S15 (G), 95% CI = 64.52%–72.85%, W = 11, *p* = 4.10 × 10^−2^, |RBC| = 0.46) and were not observed for Adapt10MFT as shown in Supplementary Figure 15 (H).

Finally, the incorporation of multiple molecular representations produced limited gains, which varied with the augmentation fold. In Supplementary Figures S16 (A) and (B), neither the BLEU scores nor the MaxFrag accuracy, respectively, showed a statistically significant improvement of Adapt5MPR_MFT over Adapt5MPR and across the Top-N range, with RBC fluctuating around zero. In contrast, Supplementary Figures S16(C) and (D) revealed a localized, statistically significant improvement only in BLEU scores for Adapt10MRP_MFT over Adapt10MPR at Top-8 (95% CI = 45.86%–54.72%, W = 568, *p* = 2.34 × 10^−2,^ |RBC| = 0.29), accompanied by small-to-medium effect size. Beyond this localized region, the two models performed comparably, suggesting that the benefits of multi-representation mixed fine-tuning were restricted to a narrow subset of Top-N predictions.

#### 3.2.7. Hybrid ensemble model

Based on the above results, it was challenging to determine the optimal ensemble model configurations and checkpoints for reliable gut biotransformation product prediction. To address this challenge, we subsequently developed a brute-force search algorithm that recursively searched across successful predictions of over 4400 checkpoint models to prioritize them based on Top-20 accuracy. Thirty-five models emerged as the winners, achieving an accumulated accuracy of 83.30% in the Top-200 predicted metabolites pool. Supplementary Figures S17 and S18 provide insights into the distribution of the “model winners” across different configurations and data partitions, respectively. Configuration C4 emerged as the optimal setup, appearing in six partitions: 2, 3, 4, 6, 7, and 9 (Supplementary Table S11), while configuration C3 was absent, indicating that it was an inappropriate configuration. Supplementary Figure S19 shows that adaptive augmentation data (Adapt10 and Adapt5), combined with two steps of FT and MFT, produced the best results when using SMILES. The high co-occurrence (eight occurrences) between “Adapt5+Adapt10” and “Two-Steps” further demonstrates how well they work together to achieve optimal performance. Similarly, the configuration “Adapt10+Two-Steps” emerged as the best-performing setup when MRP was used (Supplementary Figure S20). Moreover, Partition 2 was chosen seven times to refine basic models, as shown in Supplementary Figure S18, followed by partitions 3 and 4 (five times each) and partitions 9 and 10 (four times each). These results suggest that some data partitions better captured the chemical patterns of gut reactions, which helped improve the generalization of the base models.

Although the best base model M3 achieved the highest Top-20 BLEU score of 62.05%, the brute–force search algorithm ranked it third, allowing M1 to emerge as the best overall performer. Model M1 has configuration C8 and was mixed fine-tuned on Partition 8, achieving a BLEU score of 61.98% and an accuracy of 64.72%. In contrast, the lowest prediction quality within this elite subset was yielded by model M7, with configuration C9 (Adapt5MRP), which still maintained a respectable 51.93% BLEU score. The overall accuracy at Top-200 was 83.30%. Supplementary Figure S21 details the molecular translation quality across all thirty-five models.

Moreover, we observed that using the full set of winning base models led to significant computational overhead when predicting large batches of compounds; therefore, we implemented four levels of prediction to determine which level allowed GUTchetp to perform best while reducing prediction computational time. These levels are: Basic, which includes the first 10 base models (M1 to M10); Advanced, which leverages twenty models (M1 to M20); Exhaustive, which employs the entire set of base models; and Adaptive, which uses an agent to select the models that best fit the query compound and adds them to the Basic level. For the prediction of 479 gut biotransformation compounds, Supplementary Figure S22, Figure 3L and M, and Supplementary Figure S23, illustrate that the Adaptive level demonstrated a favorable balance between prediction quality and computational efficiency compared with the Advanced and Exhaustive prediction levels.

Supplementary Figure S22 shows that the agent selected only a single base model for the majority of compounds (262 out of 479) and four or five base models for two substantial subsets (110 and 65 compounds, respectively). As a result, significant differences were observed between the Adaptive and Exhaustive levels in the total number of base models used. The Adaptive level used a total of 5913 base models (1683 MRP and 4230 SRP), whereas the Exhaustive level utilized 16,765 base models (4311 MRP and 12,454 SRP). Although this was a discrepancy in the number of base models employed, the Adaptive level achieved BLEU scores and MaxFrag accuracy that were broadly comparable across top-N predictions (Figure 3L−M) and Supplementary Figure S24). At Top-1, the adaptive level outperformed the Exhaustive level by 1.55% in BLEU score and 1.46% in MaxFrag accuracy. It maintained the strongest performance across the mid-range, with BLEU scores increasing from 72.89% to 76.50% over Top-60 to Top-140, and MaxFrag accuracy rising from 74.74% to 77.87% over Top-60 to Top-100. At higher Top-N values, the Exhaustive level slightly overtook the Adaptive level, with BLEU score gains of 0.36%, 0.13%, and 0.60% at Top-160, Top-180, and Top-200, respectively, alongside corresponding MaxFrag accuracy improvements of 0.21%, 0.42%, 0.63%, and 1.04% from Top-120 to Top-200.

Furthermore, the Adaptive level required 323 minutes of computation time, compared with 351 minutes for the Advanced level and 854 minutes for the Exhaustive level (Supplementary Figure S23), corresponding to reductions in computational time of 8.00% and 62.20%, respectively.

Supplementary Figures S25 and S26 present the statistical significance of pairwise comparisons between each configuration model and GUTchetp for BLEU scores and MaxFrag accuracy, respectively. Each sub-figure panel (A−L) corresponds to one of the 12 configurations.

Across all comparisons, GUTchetp generally outperformed all models in both BLEU and MaxFrag accuracy, with increasing |RBC| and decreasing ORs as Top-N increased, indicating progressively stronger and more consistent differences. Compared with model A5, statistically significant differences in BLEU and MaxFrag accuracy first emerged from Top-5 (95% CI = 45.79%–54.70%, W = 867, *p* = 2.25 × 10^−2^, |RBC| = 0.26) and Top-6 (95% CI = 48.12%–57.10%, Statistic = 20, *p* = 9.85 × 10^−3^, OR = 0.49), respectively, and remained significant through Top-100, except for a non-significant interval between Top-16 and Top-18 for MaxFrag accuracy (Supplementary Figure S25 (A) and Supplementary Figure S26 (A). Compared with A5MFT, A10, Adapt5, Adapt5MFT, Adapt10, and Adapt10MFT, GUTchetp exhibited discrete regions of statistical significance for both BLEU and MaxFrag accuracy, alternating between an early significant band at low Top-N (5-15), a subsequent non-significant interval, and a broader significant region emerging at moderate-to-high Top-N (approximately 30-100), with progressively larger BLEU effect sizes and lower MaxFrag ORs across this range. A10MFT deviated from this pattern, as GUTchetp significantly outperformed A10MFT in both BLEU and MaxFrag only at Top-66 (BLEU: 95% CI = 68.96%–76.69%, W = 605.50, *p* = 3.63 × 10^−2^, |RBC| = 0.27, MaxFrag: 95% CI = 70.61%–78.45%, Statistic = 12, *p* = 4.70 × 10^−3^, OR = 0.48). By contrast, compared with the three MRP-derived models (Adapt5MRP_MFT, Adapt10MRP, Adapt10MRP_MFT), GUTchetp demonstrated consistent statistical significance across the entire Top-N range, with consistently large BLEU effect sizes at Top-1 (minimum |RBC| = 0.50, *p* = 5.43 × 10^−3^, maximum |RBC| = 0.74, *p* = 1.04 × 10^−5^) and low MaxFrag ORs (minimum OR = 0.13, *p* = 6.16 × 10^−6^, maximum OR = 0.32, *p* = 1.46 × 10^−2^). These results confirm the most substantial and consistent performance advantage of GUTchetp. Notably, when compared with Adapt5MRP, GUTchetp also achieved significant improvements over most Top-N range except at Top-1, with significance emerging from Top-2 onward.

Although A10MFT exhibited more statistically significant regions than GUTchetp, we consider GUTchetp superior because our objective is to reliably predict metabolites representing challenging biotransformations, including those with low structural similarity to the parent compound (<25%), extensive structural transformations (retaining < 25% of the parent's atomic composition), negative exact mass changes ([−914, −389]), and/or very small size (<6 atoms). The PP dataset comprises 96, 52, 14, and 6 such cases, respectively, among its 170 compounds/metabolites. Supplementary Figure S27 shows that GUTchetp surpassed A10MFT in predicting 11, and 16 more challenging cases at Top-20 and Top-200, respectively, with the clearest gains in the low-similarity criterion (33 vs. 29 in Top-20, 47 vs. 36 in Top-200) and the small-product-size criterion (7 vs. 5 in both). At Top-20, A10MFT predicted only one additional challenging case beyond those shared with GUTchetp. However, this case was also captured by GUTchetp when the evaluation was extended to Top-200.

#### 3.2.8. GUTchetp outperforms state-of-the-art methods

**Figure 4.**
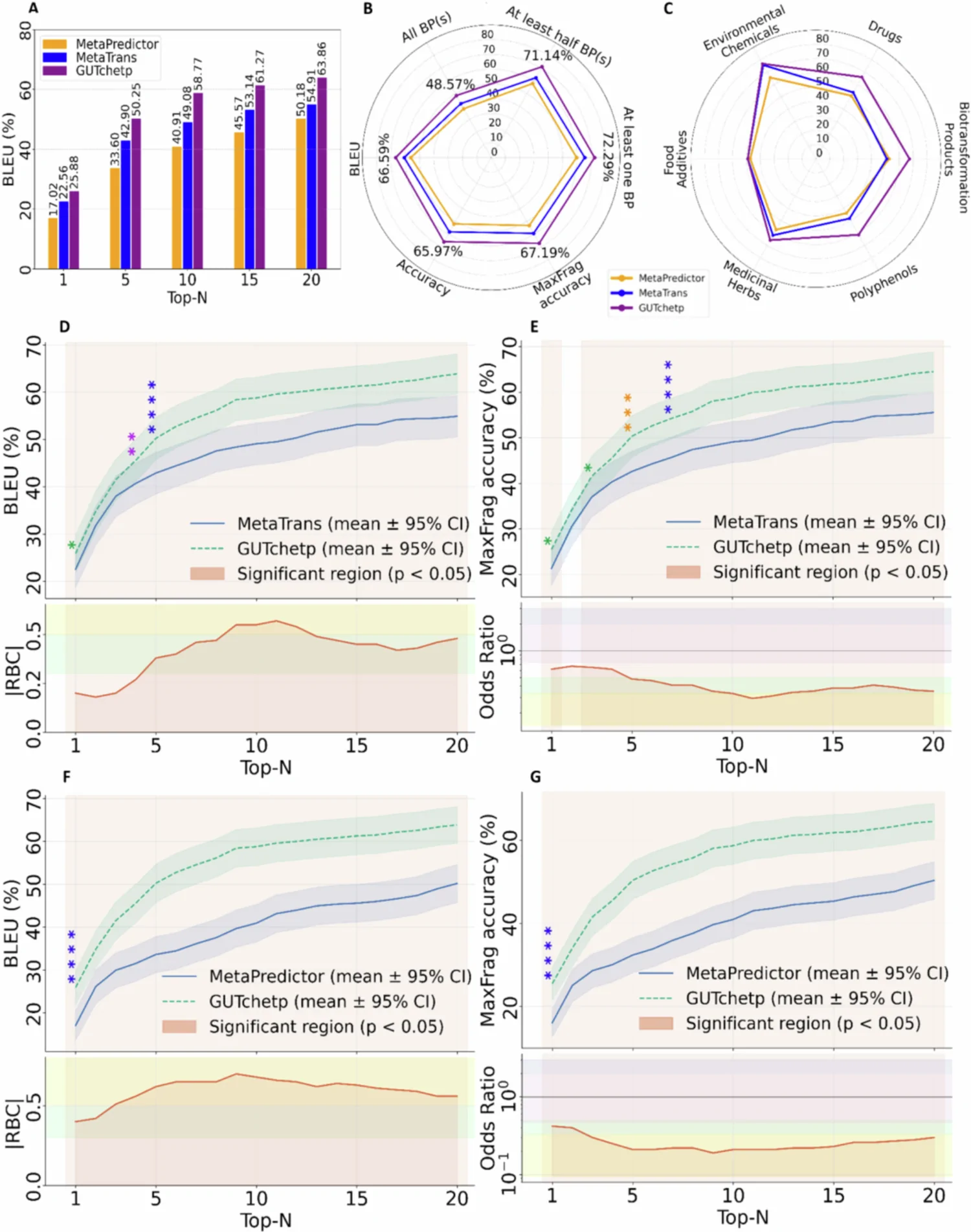
Benchmarking GUTchetp against other tools with pairwise statistical significance testing of BLEU scores and MaxFrag accuracy across Top-1 to Top-20 predictions. (A) BLEU scores for biotransformation product predictions. (B) Comparison of performance metrics at Top-20 predictions. (C) Performance across compound categories. (D, F) Pairwise comparison of BLEU scores between GUTchetp and MetaTrans (D) and MetaPredictor (F). The upper panels show one-tailed Wilcoxon signed-rank test results, and the lower panels show the absolute rank-biserial correlation (|RBC|) as the effect size. (E, G) Pairwise comparison of MaxFrag accuracy between GUTchetp and MetaTrans (E) and MetaPredictor (G). The upper panels show one-tailed McNemar's test results, and the lower panels show the odds ratio (OR; log scale) as the effect size; the solid gray line at OR = 1 indicates no effect. All values are presented as mean ± 95% CI. Details of the color scheme and panel-specific annotations are provided in Supplementary Table S12.

We assessed the predictive capability of GUTchetp for predicting gut biotransformation products with two tools. MetaTrans and MetaPredictor, which have exhibited superior prediction performance relative to three other tools, GLORYx, SyGMa, and BioTransformer. Figure 4A−C provide an extensive comparison of these three predictive models through two complementary evaluation frameworks: in Figure 4A, Supplementary Figure S28, and Figure 4C, metrics were calculated for each compound–biotransformation-product pair, whereas in Figure 4B and Supplementary Figure S29(A−D), metrics were determined for each compound.

GUTchetp consistently outperformed other tools across both evaluation frameworks, achieving the highest BLEU scores and MaxFrag accuracy at every Top-N level. Its BLEU score increased significantly from 25.88% at Top-1 to 63.86% at Top-20, surpassing both MetaTrans (blue) and MetaPredictor (orange) (Figure 4A). Similarly, MaxFrag accuracy improved from 25.47% at Top-1 to 64.51% at Top-20 (Supplementary Figure S28), further supporting the model’s superior performance in molecular fragment prediction.

Figure 4B and Supplementary Figure S29 (A−D) support this trend: GUTchetp performed better than all other Top-Ns on all metrics (BLEU, MaxFrag accuracy. At least one BP, At least half BPs, and All BPs). For instance, GUTchetp outperformed MetaTrans (56.00%) and MetaPredictor (38.29%) at Top-5, reaching up to 58.86% for “At least one BP”. This superiority became more pronounced at higher Top-15 and Top20 levels, where GUTchetp achieved 70.00%–72.29% for “At least one BP”, 67.14%–71.14% for “At least half BPs”, 47.43%–48.57% for “All BPs”, 65.66%–66.59% for BLEU, 62.63%–65.97% for Accuracy, and 64.67%–67.19% for MaxFrag accuracy; consistently outperforming the other approaches.

Moreover, the radar plot in Figure 4C demonstrates that GUTchetp consistently achieves top or near-top BLEU scores across all compound categories. Specifically, the model outperformed the other two tools by up to 12.37%, 14.56%, and 13.11% at Top-20 for drugs, biotransformation products, polyphenols, and medicinal herbs, respectively. Although the two categories, food additives and environmental chemicals, remained challenging for all approaches, GUTchetp consistently outperformed them. Supplementary Table S13 presents a comprehensive comparison of GUTchetp with the MetaTrans and MetaPredictor tools, highlighting differences in dataset scale, preprocessing pipelines, augmentation strategies, transfer learning approaches, ensemble learning frameworks, and post-processing procedures. These comparisons provide insights into how methodological choices contribute to differences in model performance and generalizability.

Across both evaluation metrics, GUTchetp consistently outperformed both benchmark tools, with statistically significant improvements supported by meaningful effect sizes. Compared with MetaTrans, GUTchetp achieved significantly higher BLEU scores across the entire Top-N predictions. The improvement also increased at higher Top-N ranges, from significant gains at Top-1 (95% CI = 21.96%–29.80%, W = 1628, *p* = 3.12 × 10^−2^, |RBC| = 0.20) to highly significant gains from Top-5 (95% CI = 45.79%–54.7%, W = 1991, *p* = 7.16 × 10^−5^, |RBC| = 0.38) onward, corresponding to a medium effect size. The large effect size begins at Top-9 (95% CI = 54.05%–62.79%, W = 1303.50, *p* = 8.65 × 10^−8^, |RBC| = 0.55), indicating statistically robust and practically meaningful performance improvements as shown in Figure 4D.

Similar results were observed relative to MetaPredictor, with significant improvements, in favor of GUTchetp, detected as early as Top-1 (95% CI = 21.96%–29.8%, W = 2323, *p* = 1.26 × 10^−5^), corresponding to a medium-to-large effect (|RBC| = 0.40), and increasing to a large effect by Top-3 (|RBC| = 0.51) (Figure 4F). MaxFrag accuracy likewise favored GUTchetp over both literature methods. Against MetaTrans, McNemar’s test revealed distinct regions of significance. GUTchetp showed a significant improvement at Top-1 (95% CI = 21.55%–29.39%, Statistic = 32, *p* = 3.75 × 10^−2^, OR = 0.62), followed by a nonsignificant result at Top-2. Significant gains resumed from Top-3 (95% CI = 37.12%–45.97%, Statistic = 40, *p* = 3.71 × 10^−2^, OR = 0.65) onward, reaching their strongest evidence at Top-7 (95% CI = 49.8%–58.76%, Statistic = 29, *p* = 4.61 × 10^−5^, OR = 0.41), corresponding to a medium-to-large effect on favor of GUTchetp (Figure 4E). Relative to MetaPredictor, McNemar's test yielded an even larger advantage for GUTchetp at Top-1 (95% CI = 21.55%–29.39%, Statistic = 32, *p* = 1.94 × 10^−2^, OR = 0.42), corresponding to a large effect size (Figure 4G). Supplementary Tables S14 and S15 provide detailed results of the statistically significant test comparing MetaTrans and MetaPredictor with GUTchetp for BLEU scores and MaxFrag accuracy, respectively.

### 3.3. Evaluation of gut microbial enzyme and species prediction

The similarity matrix was constructed based on expanded reactions from BRD (See Section 2.3.1), resulting in a matrix with dimensions of 37,537 × 37,537. Afterwards, experiments showed that the combination of Morgan (M), Pattern (P), and Topological Torsion (TT) fingerprints achieved the highest WAF1, which reached up to 88.80%, representing improvements of 2.70% and 1.47% over CLAIRE model and the Atom Pair (AP) fingerprint used by SIMMER, respectively. Supplementary Figure S30 illustrates the performance comparison between CLAIRE and 31 models trained using different combinations of five fingerprint descriptors (AP, M, RDkit, P, and TT). Rather than selecting only the single best-performing fingerprint combination, the three top-ranked combinations were subsequently subjected to five-fold cross-validation using RF1 and RF2 to predict enzyme classes at the first- and second-level EC hierarchy, respectively. Supplementary Table S16 reveals distinct optimal representation for different levels of EC classification. For the first-level EC prediction, the RF1 model with M+P+TT combination achieved the highest overall performance, reaching a WAF1 of 89.43% on the independent EP dataset, while maintaining the most consistent cross-validation performance (78.60 ± 0.09) %. Although the inclusion of AP fingerprint provided comparable cross-validation performance, it resulted in reduced generalization on the hidden dataset, indicating that this additional structural information did not improve first-level EC number classification. For the second-level EC prediction, the RF2 model with M+P fingerprint combination exhibited the strongest generalization capability, achieving the highest hidden-test WAF1score (81.02%), despite a marginally lower cross-validation score (72.32 ± 0.30) %. Notably, incorporating TT or AP fingerprints improved cross-validation estimates but did not translate into superior hidden-test performance, suggesting that these additional descriptors may introduce redundancy or increase model sensitivity to dataset-specific variations. Therefore, M+P fingerprint was selected as the optimal representation for second-level EC classification, whereas M+P+TT fingerprint provided the most effective feature combination for the first-level classification.

**Figure 5.**
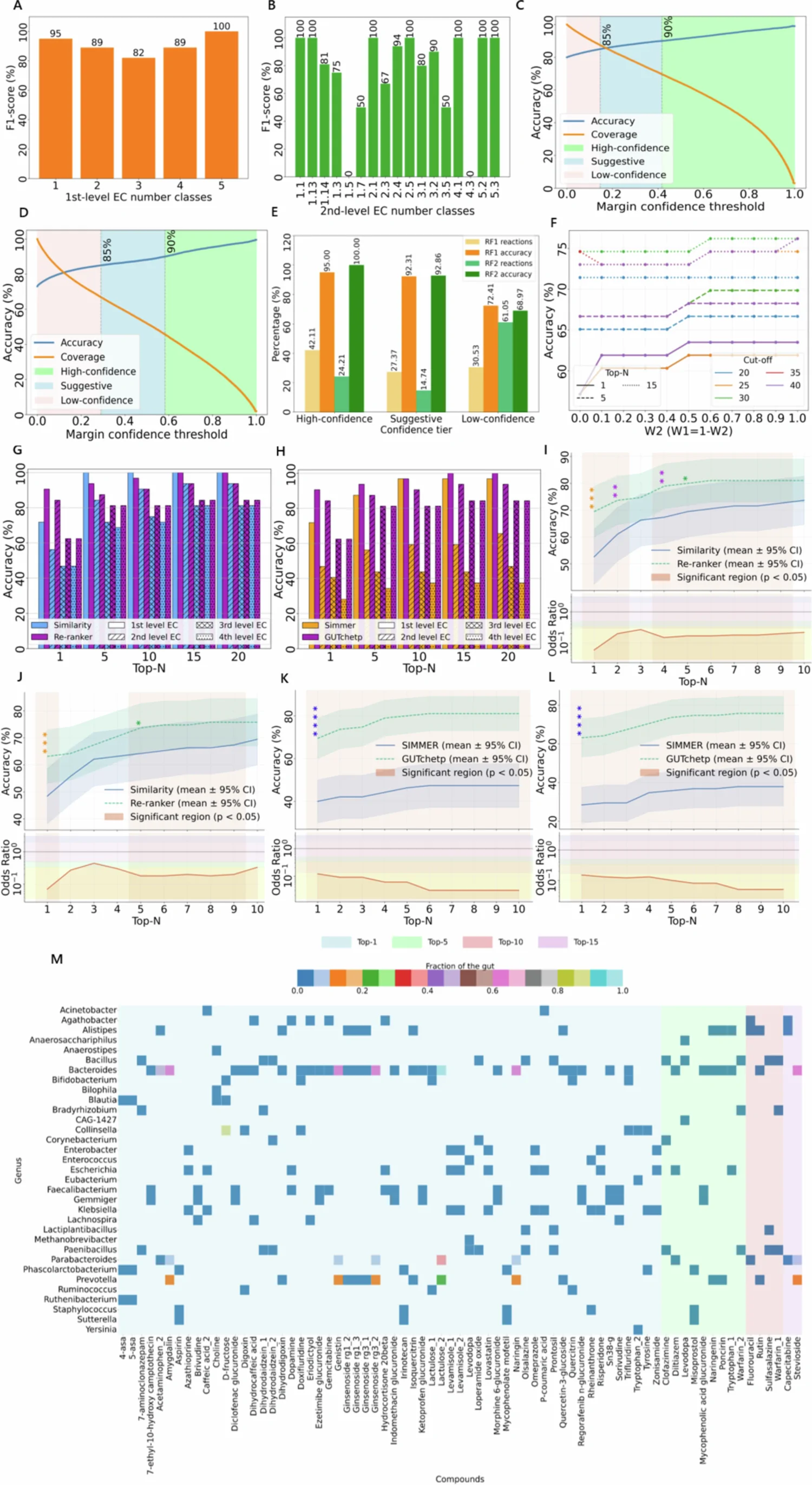
Predictive performance and statistical significance analysis of the second component of GUTchetp and gut microbial profiles. (A-B) Performance of the RF1 and RF2 classifiers at the first and second EC classification levels, respectively, using the EP dataset. (C-D) Coverage-accuracy curves for RF1 and RF2, respectively, evaluated on the OOF data. (E) Distribution of confidence tiers across reactions in on the hidden EP dataset and their corresponding accuracies (F) Evaluation of weight consistency (W1 and W2) for re-ranking reaction candidates on the hidden fold (32 reactions) of the EP dataset (G) Comparison of reaction similarity-re-ranking-based methods. (H) Comparative performance analysis of GUTchetp and SIMMER. (I-J) Statistical significance analysis comparing the reaction similarity method and GUTchetp across Top-1 to Top-10 predictions at the third and fourth EC classification levels, respectively. (K-L) Statistical significance analysis comparing SIMMER and GUTchetp across Top-1 to Top-10 predictions for the third and fourth EC classification levels, respectively. Details of the color scheme and annotations of the statistical analysis are provided in Supplementary Table S12. (M) Top three predicted gut microbes associated with their correctly predicted EC numbers. Enzyme enrichment was calculated as the ratio of enzyme proteins within the corresponding genera to the total number of enzyme proteins in the UHGG gut genome collection.

At the first-level EC number, RF1(M+P+TT) demonstrated strong performance across nearly all classes, with F1-scores ranging from 95% to 100%. The hydrolases class, on the other hand, performed the worst with F1-scores that reached up to 82% (Figure 5A). However, Figure 5B illustrates that certain subclasses posed challenges for the RF2 (M+P) model. Specifically, F1-scores for subclasses 1.7 and 3.5 were both 50%. In contrast, subclasses like 1.1, 1.13, 2.1, 2.5, 4.1, 5.2, and 5.3, attained the highest F1-score of 100%. Interestingly, subclasses 1.5 and 4.3 had zero F1-score and no detectable predictions. This variation in RF2’s model performance most likely reflects a severe class imbalance, as detailed in Supplementary Table S17, where subclasses 1.5 and 4.3 make up only 1.18% and 0.31% of the training dataset, respectively. For the third-level EC number (RF3), we also trained a random forest (M+P+TT) classifier; however, its WAF1 score was only 60.15%, indicating limited predictive reliability. As a result, RF3 was excluded from further analysis (Supplementary Figure S31).

For both models, twelve candidate accuracy pairs were selected randomly, and the target accuracy pair of 90% and 85% was then determined because it minimized the size of the ambiguous Suggestive confidence tier. As shown in the Supplementary Figure S32 (RF1) and Supplementary Figure S33 (RF2), this criterion resulted in the lowest coverage of the Suggestive tier, accounting for 17.75% and 21.16% of predictions, respectively. We next defined margin confidence thresholds for models by mapping prediction margin to empirical accuracy and coverage (Figure 5C and D). For RF1, a High-confidence threshold of 0.4174 and a Suggestive threshold of 0.1466 partition margin predictions into three confidence tiers: High-confidence (margin ≥ 0.4174), retaining 69.66% of predictions with a mean out-of-fold accuracy of 90.00%; Suggestive (0.1466 ≤ margin < 0.4174, comprising 17.75% of predictions with an accuracy of 65.40%; and Low-confidence (margin < 0.1466), accounting for the remaining 12.59% with an accuracy of 45.00%. For RF2, the corresponding thresholds were 0.5831 and 0.2907, yielding a High-confidence tier that retained 45.66% of predictions with a mean out-of-fold accuracy of 90%, a Suggestive tier that comprised 21.16% of predictions with an accuracy of 74.21%, and a Low-confidence tier that included the remaining 33.18% of predictions with an accuracy of 48.96%. Supplementary Table S18 summarizes the margin confidence thresholds and their associated accuracy and coverage for each model.

Applying these thresholds to the hidden dataset EP, RF1 classified 42.11% of reactions as High-confidence, 27.32% as Suggestive, and 30.53% as Low-confidence. The corresponding accuracies were 95.00%, 92.31%, and 72.41%, respectively. In contrast, RF2 was markedly skewed towards the Low-confidence tier, producing 24.21% High-confidence, 14.74% Suggestive, and 61.05% Low-confidence predictions (Figure 5E), with corresponding accuracies of 100.00%, 92.86%, and 68.97%, respectively.

A total of 95 reactions from the EP dataset were repeatedly queried against the similarity matrix to identify candidate enzymatic reactions. These candidates were then re-ranked using a cut-off of 30, together with weights of 0.3 and 0.7 for correct predictions at first- and second-level EC numbers, respectively. These parameters were determined using three-fold cross-validation on 67% (63/95) of the EP dataset and were considered optimal because they produced stable predictions across all Top-N values, as shown in Figure 5F. Moreover, it can be noted that Top-N accuracies were the lowest across all cutoffs when second-level EC number predictions were completely omitted (W1 = 1.0 and W2 = 0.0). Despite Top-N accuracies being higher when first-level EC number predictions were completely omitted (W1 = 0.0), we did not consider these settings as optimal because our goal was to minimize prediction failures for certain classes and subclasses and to benefit from the combined decisions of RF1 and RF2 classifiers.

Figure 5G shows that re-ranking the hidden reactions (32/95) substantially improved Top-1 prediction accuracy across all EC-number levels compared with ranking based solely on reaction similarity. Specifically, the Top-1 accuracies increased from 71.87%, 56.25%, 46.87%, and 46.87% to 90.62%, 84.37%, 62.50%, and 62.50% for the first through fourth EC-number levels, respectively. Similar improvements were observed for the third and fourth EC-number levels across all the remaining Top-N values, while the first and the second EC-number maintained unchanged at Top-15 and Top-20. Comparing GUTchetp with SIMMER, our results in Figure 5H confirm that GUTchetp predicted the gut enzymes correctly with a higher Top-20 accuracy of up to 84.38% (27 compounds out of 33), compared to 37.50% (12 compounds) for SIMMER. Notably, the enzyme pyrimidine-nucleoside phosphorylase (EC:2.4.2.2, reaction R01570) for brivudine gut biotransformation was correctly predicted, whereas SIMMER made no significant prediction.^27^ Moreover, GUTchetp consistently outperformed SIMMER, achieving higher Top-1 accuracy with differences of up to 18.75% (6 compounds) at the first-level and 37.50% (12 compounds) at the second-level, 21.88% (7 compounds) at the third-level, and 34.38% (11 compounds) at the fourth-level of EC numbers. Using the whole EP dataset, Supplementary Figures S34 (A) and S34 (B) further demonstrate that the observed improvements were not limited to the hidden dataset. GUTchetp consistently outperformed the reaction similarity method and SIMMER for Top-1 EC-number prediction, correctly predicting 63.16% of reactions (60 Total), compared with 48.42% (46 reactions) for the reaction similarity method and 28.42% (27 reactions) for SIMMER.

Statistical significance analysis also demonstrated the performance advantages of GUTchetp (Re-ranker) over reaction similarity as well as over SIMMER. Compared with the reaction similarity method, Re-ranker consistently achieved significant improvements, although the magnitude and consistency of these gains varied across EC classification levels. At the first EC level, significant improvement was observed only at Top-1 (95% CI = 85.89%–97.27%, Statistic = 2, *p* = 1.05 × 10^−5^, OR = 0.08), after which the difference became non-significant as both approaches rapidly converged in performance (Supplementary Figure 35 (A)). At the second level, the advantage was similarly limited to the earliest predictions, with statistical significance observed only from Top-1 (95% CI = 75.49%–90.82%, Statistic = 2, *p* = 1.62 × 10^−6^, OR = 0.07) to Top-4 (Statistic = 2, *p* = 2.25 × 10^−2^, OR = 0.18), corresponding to a large effect size favoring the Re-ranker (Supplementary Figure 35 (B)).

At the third level, the results exhibited only one discontinuous region of statistical insignificance at Top-3 (Figure 5I). In contrast, statistically significant improvements were observed across all remaining Top-N ranges, including Top-1 (95% CI = 60.04%–78.9%, Statistic = 1, *p* = 1.45 × 10^−4^, OR = 0.06), Top-2, and Top-4 through Top-9. Similarly, at the fourth level, significant improvements were observed at Top-1(95% C = 53.28%–73.04%, Statistic = 1, *p* = 5.19 × 10^−4^, OR = 0.07) and Top-5 through Top-9, while no significant differences were observed for Top-2 through Top-4 and Top-10 onward (Figure 5J).

Compared with SIMMER, GUTchetp demonstrated stronger and more consistent statistical evidence across EC classification levels. At the first level, statistically significant improvements were observed from Top-1 (95% CI = 85.89%–97.27%, Statistic = 6, *p* = 1.43 × 10^−5^, OR = 0.18) through Top-6 (Statistic = 5, *p* = 4.14 × 10^−2^, OR = 0.33), while higher accuracy at larger Top-N ranges did not reach statistical significance (Supplementary Figure 36(A)). In contrast, at the second, third, and fourth levels, statistically significant improvements were maintained across all evaluated Top-N values (Supplementary Figure 36(B), Figure 5 K and L). At Top-1, the corresponding effect size were OR = 0.08 (95% CI = 75.49%–90.82%, Statistic = 3, *p* = 1.05 × 10^−8^), OR = 0.12 (95% CI = 60.04%–78.9%, Statistic = 4, *p* = 1.94 × 10^−6^), and OR = 0.15 (95% CI = 53.28%–73.04%, Statistic = 6, *p* = 5.42 × 10^−7^) for the second, third, and fourth EC levels, respectively.

Across both comparisons, the corresponding odds ratios for all the statistically significant results indicated large effect sizes favoring GUTchetp. Supplementary Table S19 provides detailed results of the statistically significant test comparing the reaction similarity method and SIMMER with GUTchetp.

Finally, Figure 5M presents a heatmap of enzyme enrichment for the three most abundant gut microbial genera. The heatmap was generated by cross-referencing correctly predicted product-EC pairs with the HGRESC catalogue. Due to incomplete enzyme annotations in the catalogue, three product-EC pairs were excluded, affecting only 5% of the Top-1 predictions (3/60).

### 3.4. Evaluation of gut microbial biotransformation profiles

The overall accuracy of predicting human gut microbial biotransformation profiles (biotransformation products with their associated enzymes and species) for 95 compounds was 53.68% (51/95), 62.11% (59/95), 66.32% (63/95), and 68.48% (65/95) at Top-1, Top-5, Top-10, and Top-15, respectively (Supplementary Figure (S37)).

### 3.5. Case studies in gut microbial biotransformation profile prediction

#### 3.5.1. Single-step prediction cases

**Figure 6.**
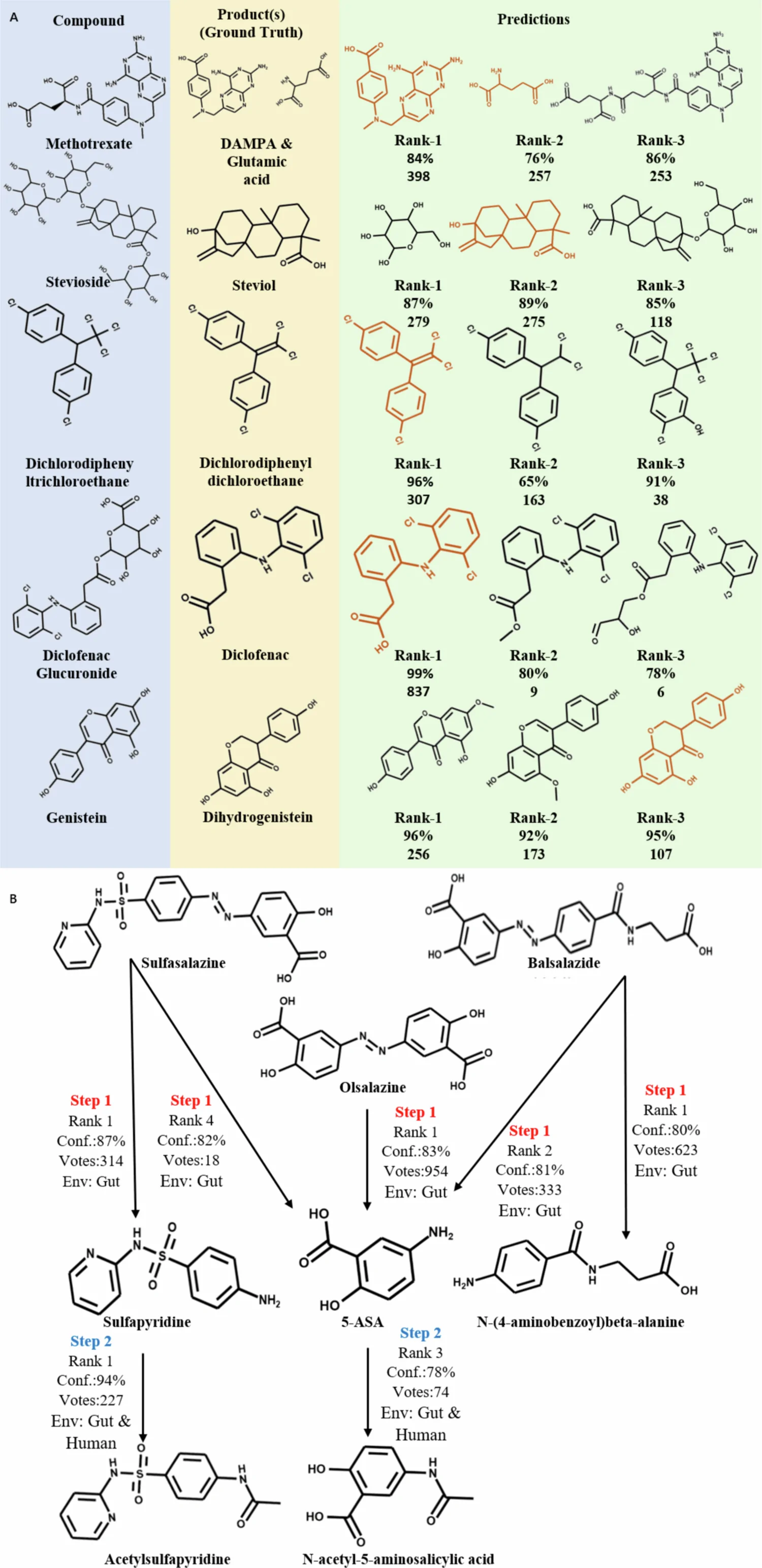
Analysis of single-step and multiple-step predictions. (A) Top-3 predictions by GUTchetp for five molecular categories. The blue background highlights the structure of the query compound, while the orange background corresponds to the ground-truth structure of the known biotransformation product. The green background illustrates the top-three predicted metabolites generated by GUTchetp. Correctly predicted structures are highlighted in dark orange for emphasis. (B) Multi-step predictions for three drugs (Sulfasalazine, Olsalazine, and Balsalazide).

To gain intuitive insights into the predictions of gut biotransformation products, we visually examined five selected molecules from our test dataset alongside the top three predictions generated by the Basic level of GUTchetp. Figure 6A illustrates the Top-3 predicted metabolic products of representative molecules that span five categories: methotrexate (a drug), stevioside (a food additive), dichlorodiphenyltrichloroethane (an environmental chemical), diclofenac glucuronide (an active metabolite), and genistein (a polyphenol).

The first example is methotrexate (MTX), which is used to treat many kinds of cancer or severe and resistant forms of autoimmune diseases and is metabolized by intestinal bacteria to the inactive metabolites 4-amino-4-deoxy-N-methylpteroic acid (DAMPA) and glutamate.^57^ These two metabolites were correctly identified by our approach, assigning them Rank-1 and Rank-2 with a score confidence of 84% (398 votes) and 76% (257 votes), respectively. As a result, GUTchetp could effectively mimic the enzymatic activity of bacterial carboxypeptidase glutamate 2 (CPDG2, EC:3.4.17.11), which cleaves MTX to produce these two known metabolites. The hierarchical classifiers RF1 and RF2 correctly predicted the first- and second-level EC numbers (3 and 3.4) with margin confidence scores of 0.64 and 0.73, respectively. Both predictions were assigned to the high-confidence tier. In total, 2708 unique bacterial protein sequences were predicted to be putatively capable of MTX hydrolysis, with *Phascolarctobacterium*, *Sutterella* and *Acidaminococcus* identified as the Top-3 most frequently occurring genera (Supplementary Figure S38).

The second case study is the transformation of stevioside to steviol. Stevioside is known as a low-calorie sweetener that has become a common substitute for sucrose in modern diets^58^. The correct product was ranked first with 92% of structural confidence and 263 votes, reflecting the ability of the ensemble model to correctly identify glycosidic bond cleavage reactions. This transformation is carried out by beta-glucosidase enzymes (EC:3.2.1.21) produced by intestinal bacteria, specifically species within the genera *Bacteroides.* ^59^ The RF1 and RF2 classifiers predicted this gut reaction to be non-enzymatic, with margin confidence scores of 0.20 and 0.50, respectively, placing both predictions in the Suggestive tier. This misclassification is attributable to the fact that the reactions generated from the expansion of MNXR116724 and MNXR118794^34^ reactions are associated with multiple enzyme annotations (non-enzymatic and EC:3.2.1.-). Consequently, the re-ranker promoted the correct enzyme from fourth place (based on similarity reactions) to the second place. Supplementary Figure S39 further illustrates that the top predicted genera are *Bacteroides*.

The third case study involves dichlorodiphenyltrichloroethane (DDT), which has been associated with an increased risk of breast, liver and testicular cancers and metabolic diseases.^60^ DDT is metabolized to dichlorodiphenyldichloroethane (DDD) through the removal of one chlorine atom. GUTchetp demonstrated the ability to capture this known microbial reductive dichlorination activity, assigning it Rank-1 with a structural confidence of 96% and 307 votes. The two classifiers, RF1 and RF2, correctly predicted the EC number at the first- and second-levels (EC 4 and EC 4.5) with margin confidence scores of 0.56 and 0.48, placing the predictions in the High-confidence and Suggestive tiers, respectively. The predicted taxonomic distribution is depicted in the Supplementary Figure S40, where we can note that this gut biotransformation is primarily associated with the genus *Escherichia*. Notably, early studies of mammalian gut microflora reported that *Proteus vulgaris* can dechlorinate DDT to DDD,^61^ which is also reflected in our predicted taxonomy.

Similarly, the first prediction of diclofenac glucuronide aligns with the ground truth (diclofenac), receiving 837 votes and a structural confidence of 99%. This result demonstrates the model’s strong performance in predicting glucuronidation-related transformations catalyzed by bacterial beta-glucuronidase (EC:3.2.1.31), which was ranked 25th among the candidate reactions. The hierarchical classifiers RF1 and RF2 correctly predicted the first- and second-level EC classes (3. and 3.2) with margin confidence scores of 0.52 and 0.50, placing the predictions in the high-confidence and suggestive tiers, respectively. In total, 2449 unique bacterial protein sequences were predicted to be capable of mediating glucuronide deconjugation. The predicted taxonomic profiling revealed that these sequences were predominantly associated with the phyla *Proteobacteria*, *Firmicutes* and *Bacteroidetes* (Supplementary Figure S41), which are consistent with previous reports in the literature.^62^

The final case study concerns the double-bond reduction reaction of the polyphenol genistein to dihydrogenistein,^63^ a reaction catalyzed by daidzein reductase (DZNR) in the human gut microbiota. DZNR is mechanistically consistent with EC:1.3.1.-, although no specific EC number has been assigned. The Rank-1 and Rank-2 product predictions are inaccurate, while the Rank-3 prediction matches the ground truth with 62 votes and 96% structural confidence. Based on reaction similarity, the top-ranked gut reactions were R03590, R13069, and R12279, all of which were associated with oxygen-dependent oxidative chemistry (EC:1.14.19.76) or lacked EC annotation. Consequently, the putative correct EC class was ranked 65th via reaction R07804; however, this reaction is unrelated to genistein biotransformation and belongs to aromatic hydrocarbon degradation.

Hierarchical re-ranking further misclassified the predicted transformation as non-enzymatic. Both mispredictions could be attributed to the minor structural change between substrate and product, which presents a challenge for the NMT algorithms, the lack of double-bond reduction reactions in the fine-tuning dataset, or the fact that many reactions are catalyzed by multiple enzymes.

#### 3.5.2. Multi-step cases

To validate GUTchetp's applicability in reconstructing metabolic pathways within the gut environment, we further expanded its single-step prediction to encompass multi-step gut metabolite predictions by successively predicting each individual enzymatic reaction. We validated our approach using three examples reported in the literature, including sulfasalazine, olsalazine, and balsalazide. As shown in Figure 6B, our method successfully predicts the complete metabolic pathways for these compounds. Sulfasalazine, olsalazine, and balsalazide are widely used in the treatment of inflammatory bowel disease (IBD) and are first metabolized to 5-aminosalicylic acid (5-ASA) and then to N-acetyl-5-aminosalicylic acid.^64^ Our model accurately predicted the first step microbial azo-reduction for the three drugs with a high structural confidence exceeding 81% and ranked them at the Top-4, Top-1, and Top-2 with 18, 954, and 333 votes, respectively. N-(4-aminobenzoyl) beta-alanine, the second metabolite of balsalazide, was also accurately predicted as the top choice with a structural confidence of 80% and 623 votes.

During the second step, N-acetyl-5-aminosalicylic acid was also correctly predicted with a structural confidence score of 73% and 74 votes, ranking third. Similarly, the model was able to capture the complete metabolic network of sulfasalazine, predicting sulfapyridine as the top-ranked metabolite (87% structural confidence, 314 votes), followed by acetylsulfapyridine (94% structural confidence, 227 votes, seventh rank).

The three reactions sulfasalazine → 5-ASA+sulfapyridine, olsalazine → 5-ASA, and balsalazide → 5-ASA+*N*-(4-aminobenzoyl) beta-alanine are cleaved by bacterial azo-reductase (EC:1.7.1.6) produced by anaerobic species such as *Bacteroides fragilis*, *Escherichia coli*, *Clostridium*, and *Eubacterium*. Although the generic enzyme EC:1.7.1.- was ranked highest based on the reaction similarity method, no records were found in HGRESC, which prevented us from identifying the corresponding species. Additionally, both RF1 and RF2 classifiers misclassified these reactions.

Gut bacteria further convert sulfapyridine to acetylsulfapyridine via bacterial N‑acetyltransferase (NAT) activity. This reaction was predicted correctly at Top-1 based on the reaction similarity method, suggesting EC:2.3.1.56 as the most likely match. While RF1 misclassified this reaction, RF2 correctly predicted the enzyme subclass 2.3 with a margin of confidence of 0.03, corresponding to the Low-confidence tier. However, HGRESC contains no records to annotate the corresponding species. Finally, the conversion of 5-ASA to N-acetyl-5-ASA was correctly annotated by the enzyme EC:2.3.1.56, where the hierarchical re-ranking promoted the predicted reaction from the second to the first position. Therefore, RF1 and RF2 correctly predicted the first- and second-level EC numbers (2 and 2.3) with margin confidence scores of 0.16 and 0.35, placing the predictions in the Low-confidence and Suggestive tiers, respectively. Notably, GUTchetp also correctly predicted that the last two transformations could occur in both human and gut environments.^65^ ^,^ ^66^

## 4. Discussion

Existing approaches provide valuable insights into metabolites and enzymatic reaction predictions. Nevertheless, they remain constrained by their ability to generalize effectively to unseen data. They often fail to account for the full diversity of gut microorganisms and metabolites, hindering their predictive accuracy across diverse chemical scenarios. In contrast, GUTchetp, with its comprehensive fine-tuning, validation, and test datasets (PP and EP), offers a scalable framework to predict gut microbial biotransformation profiles. In this discussion, we outline the strengths and limitations of our approach and explore its added value compared to MetaPredictor and MetaTrans.

### 4.1. Strengths

The first component of GUTchetp for predicting gut biotransformation products utilizes an ensemble of thirty-five base models, which incorporate both monolingual and multi-way multilingual neural machine translation models. These models were trained using a two-step of transfer learning process combined with a mixed fine-tuning strategy. The MRP models were trained simultaneously on four molecular representations of reactant-product pairs along with adaptive data augmentation. Extensive experiments on our benchmark dataset (PP) demonstrated promising performance, achieving a Top-20 BLEU score of 66.59% and a MaxFrag accuracy of 67.19%. For the Top-1 BLEU score, GUTchetp significantly outperformed MetaTrans (95% CI = 21.96%–29.80%, *p* = 3.12 × 10^−2^, |RBC| = 0.20) and MetaPredictor (95% CI = 21.96%–29.8%, *p* = 1.26 × 10^−5^, |RBC| = 0.40), corresponding to medium-to-large effect sizes. Similarly, for Top-1 MaxFrag accuracy, GUTchetp showed statistically significant gains compared with MetaTrans (95% CI = 21.55%–29.39%, *p* = 3.75 × 10^−2^, OR = 0.62) and MetaPredictor (95% CI = 21.55%–29.39%, *p* = 1.94 × 10^−5^, OR = 0.42), confirming the statistical superiority of GUTchetp in predict gut biotransformation products over competing methods. Thereby, the consistent improvements observed across metrics suggest that GUTchetp not only generates predictions that are globally more similar to the ground truth but also more accurately captures the key structural transformations underlying gut microbial metabolism.

The key strength of these statistical significance results lies in demonstrating GUTchetp’s ability to derive benefits from multiple configurations. Although improvements in BLEU scores and MaxFrag accuracy for several individual configurations did not reach statistical significance, their complementary contributions collectively enhanced the overall effectiveness of the tool. This suggests that GUTchetp is robust across configurations and that its performance is not dependent on a single optimization setting.

The BLEU metric identified more statistically significant and continuous performance regions in which MaxFrag accuracy failed to detect meaningful differences. This behavior was illustrated, for example, in Figure 4D versus Figure 4E, Supplementary Figure S16 (C) versus (D), and Supplementary Figures S25 (B), (E), (F), (G), and (H) versus the corresponding MaxFrag accuracy panels in Supplementary Figures S26 (B), (E), (F), (G), and (H). This difference arose because MaxFrag accuracy assigned an identical score to partially correct and completely incorrect predictions, as it only considered exact matching of the largest molecular fragment and did not account for partial structural similarity. In contrast, BLEU captured incremental improvements in structural similarity that were overlooked by MaxFrag accuracy. This property is particularly important for gut biotransformation products prediction, where multiple structurally related products can result from similar biotransformation reactions, and small differences in SMILES tokens could correspond to chemically plausible product variants rather than fundamentally incorrect predictions. As a result, BLEU revealed performance differences that remained undetected when using MaxFrag accuracy alone, therefore, BLEU was used as the primary evaluation metric for the remaining analyses.

**Figure 7.**
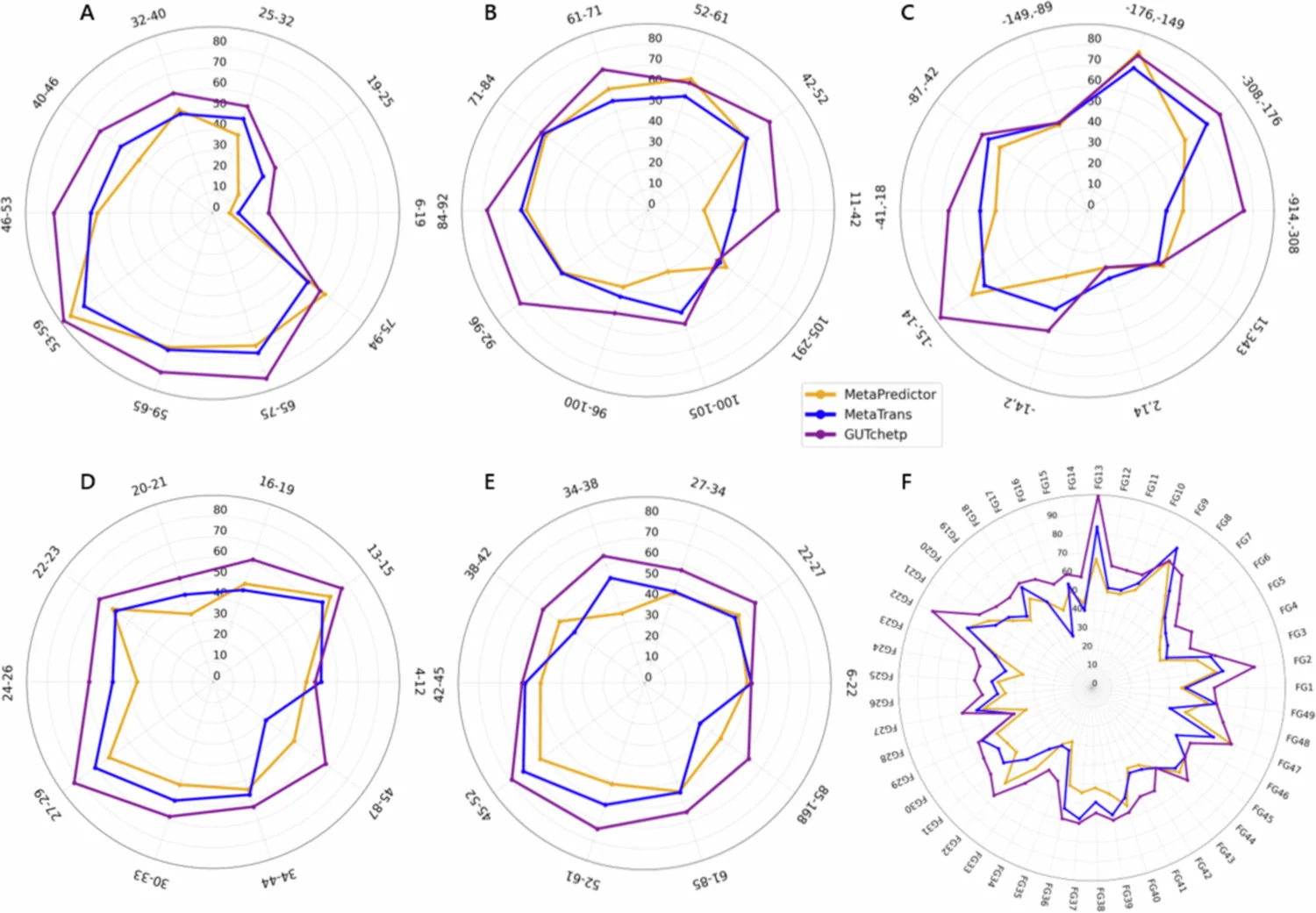
Strengths and limitations of GUTchetp for predicting gut biotransformation products in comparison with MetaTrans and MetaPredictor using BLEU scores. (A) Similarity between parent compounds and biotransformation products (%). (B) Transformation ratios (%). (C) Exact mass changes (Da). (D) Compound atom counts. (E) Compound SMILES lengths (number of characters). (F) Compound functional groups.

GUTchetp particularly showed superior generalization on reactions in which the biotransformation products are less similar to the parent compounds (Figure 7A and Supplementary Figure S42(A−D)), have low transformation ratios (Figure 7B and Supplementary Figure S43(A−D)), undergo large mass losses (Figure 7C and Supplementary Figure S44(A−D)), involve compounds with small numbers of atoms (Figure 7D and Supplementary Figure S45(A−D)), have varying SMILES length in characters (Figure 7E and Supplementary Figure S46(A-D)), and include chiral transformations as well as ring-opening and ring-forming reactions (Supplementary Figure S47).

Figure 7A confirms that GUTchetp achieved superior performance across nine similarity bins and particularly excelled in the similarity range between 53% and 59% (three bins). Additional strengths are displayed in Figure 7B, which shows that GUTchetp captured more diverse transformation ratio scenarios, especially those scenarios that span seven bins: 11%–52% (two bins), 61%–71%, and 84%–105% (four bins). Similar observations are also depicted in Figures 7C−E, which confirm the superior performance of our approach across bins ranging from −308 Da to 308 Da and from −14 Da to 42 Da for mass changes, from 13 to 87 (nine bins) for compound atomic size, and from 22 to 168 characters (nine bins) for compound SMILES lengths, respectively.

In addition to that, Figure 7F reveals that GUTchetp is able to predict a substantially broader chemical space, with notable Top-20 performance for compounds that contain functional groups such as aromatic carboxylic acids (FG2), alkyl halides (FG13), azo groups (FG22), hydroxylamine (FG32), and tertiary alicyclic amines (FG47). Notably, GUTchetp successfully predicted gut biotransformation products at Top-1 (Supplementary Figure S48 (A)), even for parent compounds with challenging functional groups. For example, MetaTrans was not able to predict products of the metabolite indicine, which contains a quaternary nitrogen group (FG45). Similarly, MetaPredictor failed to predict dichlorodiphenyldichloroethane at Top-1, the metabolite of the insecticide dichlorodiphenyltrichloroethane containing alkyl halides (FG13), and also was not able to identify levametabol I, the metabolite of the medication levametabol, characterized by amidine groups (FG16), at both Top-1 and Top-5, while still maintaining the lowest BLEU scores across all Top-N (Supplementary Figure S42 (A−D)).

The strength of GUTchetp is evident from the comparative analysis of 160 chemical and structural features. At the Top-20, it outperformed MetaPredictor in 148 features and MetaTrans in 149 features, while surpassing both tools simultaneously in 143 (89.38%) features across a wide range of gut biotransformations. GUTchetp performed equally to both tools for the only two features where compounds contain “quaternary nitrogen” or/and “piperidine ring” functional groups. Full details of this comparison are provided in Supplementary Table S20.

A further strength of GUTchetp in predicting gut biotransformation products lies in its adaptive prediction agent, which select automatically the number of base models based on the specific characteristics of the queried compound. The Adaptive level achieved superior and, in some cases, comparable, performance while requiring shorter prediction time compared to the Exhaustive level (which employs all 35 base models), particularly at Top-1 and mid-range predictions (Figure 3L−M). Notably, this efficiency reduced computational demands by up to 62.20% when predicting 479 compound-product pairs.

On the other hand, a key strength of GUTchetp for enzymes and species prediction lies in the construction of a large-scale reaction similarity matrix derived from expanded gut reactions, each of which has only one product and no cofactors.

A second key strength is the use of two random forest classifiers to predict the first (RF1) level of the EC hierarchy using the combination of three descriptor sets (M+P+TT), and the second level (RF2) using the combination of two (M+P) descriptor sets. The use of local substructure, topological, and pattern-based features along with a reaction-difference fingerprint for representing enzymatic reactions provided a chemically intuitive encoding of gut transformation patterns and enables generalization across structurally diverse substrates. Our approach led to strong WAF1 scores of 89.43% and 81.02% for predicting first- and second-level EC numbers, respectively, with perfect performance for several enzyme classes and subclasses.

Moreover, our proposed re-ranking rule (Equations (9) and (10)), which combines prediction consistency between first-level and second-level enzyme classifiers with chemical similarity, substantially improved Top-1 accuracy for complete EC number annotation, achieving a gain of 34.38% percentage points over SIMMER on the hidden dataset. This observed improvement over SIMMER was statistically significant (95% CI = 53.28%−73.04%, *p* = 5.42 × 10^−7^, OR = 0.15), demonstrating that integrating mechanistic classification with structural similarity can resolve ambiguities that could not be captured by either strategy alone.

A further strength of this study is the development of the HGRESC catalogue that links gut microbial enzymatic reactions with their corresponding microbial species. This catalogue enables large-scale exploration of enzyme-species relationships, facilitates functional interpretation of gut microbiome metabolism, and offers a practical foundation for hypothesis generation in microbiome-drug and microbiome-xenobiotic interaction studies.

Finally, the ability of GUTchetp to predict accurately 51 (53.68%) and 65 (68.42%) out of 95 known gut microbial biotransformation profiles at the Top-1 and Top-15, respectively, builds confidence to infer previously uncharacterized profiles. This new approach enables microbiome researchers to make informed, testable hypotheses and to prioritize targets before embarking on the lengthy laboratory experiments required to characterize bacterial enzymes that can alter human ingested compounds.

### 4.2. Limitations

Although GUTchetp achieves promising performance, there are several limitations in our proposed method that deserve further research in the future.

Firstly, a major limitation of GUTchetp for predicting biotransformation products is its lower Top-1 prediction accuracy, with substantially improved performance at higher Top-N values. This observation is evident in Figure 3L−M, Figure 4A−B, and Supplementary Figures S29 (A−D), where BLEU scores increased from 50.25% at Top-5 to 78.07% at Top-200, and from 35.01% at Top-1 to 66.59% at Top-20, when evaluated per metabolite and per compound, respectively. A similar trend was observed when GUTchetp was evaluated across molecular categories as well as structural and chemical features (Supplementary Figures (S42-S49)). This limitation is largely due to the fact that our dataset involves 4644 gut species, each with potentially distinct metabolic capabilities and interactions. Therefore, for a given compound, multiple metabolites may be chemically plausible and may be produced under different gut species conditions.

Secondly, although the hybrid ensemble model that combines monolingual and multilingual neural machine translation methods with adaptive augmentation improved the overall performance, GUTchetp exhibited low BLEU scores at Top-1 for specific reaction subsets. In particular, performance declined for reactions with the following characteristics: i) high substrate-product similarity that ranges between 75% and 94% (Supplementary Figure S42(A)), ii) a high transformation ratio, greater than 96% (Supplementary Figure S43(A)), iii) medium-sized substrates containing 24–26 atoms (Supplementary Figure S45(A)), and iv) ring formation (Supplementary Figure (S47)). In addition, GUTchetp failed to predict the biotransformation products of 30 compounds from the PP dataset at Top-1 when the mass gain ranged from 2 Da to 14 Da (Supplementary Figure S44 (A)).

Two solutions could address the two aforementioned limitations. The first is the use of back-translation-based data augmentation, in which reaction products are translated by a pre-existing retrosynthesis tool into reaction reactants (reverse translation direction). The obtained synthetic source reactants, along with their respective target products, are then used to construct a synthetic parallel reaction corpus. Additionally, different beam sizes should be tested to optimize the diversity and quality of the generated gut biotransformation products. The second solution is the integration of the existing tools MetaTrans and MetaPredictor with GUTchetp, in order to leverage their respective strengths and improve generalization performance.

Moreover, the absence of statistical significance across individual configurations suggests several directions for future research. In particular, future studies should employ larger and more diverse test datasets, and evaluate the robustness of the observed differences across chemical notation pairs. Additionally, complementing BLEU with other translation evaluation metrics, such as COMET or ROUGE,^52^ could also provide a more comprehensive assessment of chemical translation quality. These metrics would help determine whether the observed numerical improvements are consistent across different aspects of translation quality, even when differences in BLEU alone do not reach statistical significance.

Although GUTchetp showed strong strengths in predicting gut enzymes and microbes, it still faces significant limitations. Firstly, predictive performance decreased significantly at finer levels of the EC number hierarchy. The RF3 classifier was excluded due to a low WAF1 score of only 60.15%. The main cause of this performance drop is class imbalance, which also led the RF2 classifier to produce zero F1-scores for rare subclasses, particularly 1.5 and 4.3. The skewed class distribution limits GUTchetp’s ability to generalize to underrepresented enzymatic reactions. Consequently, a high proportion of predictions were assigned low confidence, making class discrimination more challenging and leading to lower prediction margins.

The second limitation concerns the reaction-difference fingerprint and the expansion of gut reactions with multiple products into separate reactions. Although these two representations capture the overall chemical transformation between the reactants and products, they omit critical reaction context, such as cofactors, that influences enzyme specificity. The expansion of the maltose-phosphorylase (R01555; EC:2.4.1.8) reaction and maltose glucohydrolase (R00028; EC:3.2.1.20) reaction provides a representative example. Both reactions generate the same simplified reaction, Maltose < = > 2 D-Glucose, which is associated with two enzymes. Consequently, because the EC classifiers were trained under a single-label classification scheme, such reactions introduced label ambiguity that both classifiers, RF1 and RF2, cannot resolve, and this contributes to misclassification. This example justifies the misclassification of the stevioside → steviol and genistein → dihydrogenistein reactions, as discussed in Section 3.4.1. Overall, this label ambiguity affected the classification of 4.21% of the EP dataset (4 reactions).

Two potential solutions to the above three limitations are: first, incorporating data augmentation or transfer learning strategies, as well as using class-weighted loss functions to mitigate class imbalance and expanding the number of training enzymatic reactions for underrepresented EC classes and subclasses. Second, implementing a multi-label classification framework can improve the identification of reactions catalyzed by multiple distinct enzymes.

Thirdly, the re-ranking weights and cut-off parameters were empirically determined based on a short list of reactions from the EP dataset, which may introduce dataset-specific bias. Therefore, their generalization to other biochemical or microbial reaction spaces remains to be tested.

Three final limitations of GUTchetp merit consideration. Firstly, the inability of GUTchetp to discriminate between biotransformed and non-biotransformed compounds may lead to false- positive predictions of gut biotransformation products. Secondly, GUTchetp was trained and tested for predicting immediate products in gut microbial biotransformation profiles, and its predictive performance for extended multi-step biotransformation pathways remains untested. Thirdly, HGRESC contains certain reaction and enzyme data that are not associated with any annotated microbial species. Therefore, future work will address these limitations through the i) incorporation of a binary classifier trained to filter non-biotransformed compounds prior to prediction, ii) prediction of longer pathways by leveraging curated datasets with richer and experimentally validated gut metabolic pathways, and adopting graph-based learning approaches to better capture the topological structure of multi-step metabolic networks, and iii) integration of new microbial and functional annotation data from the recently published Human Reference Gut Microbiome (HRGM2)^67^ catalogue to address this data gap and improve completeness and biological interpretability of predicted pathways.

These limitations leave room for future improvements. Integrating machine learning algorithms with Large Language Models (LLMs) emerges as a particularly promising avenue in this regard. LLMs trained on large corpora of gut microbial biotransformation literature and reaction datasets can provide rich contextual representations of enzymatic reactions, capturing cofactor dependencies and other chemical nuances that current fingerprint-based approaches cannot detect. This approach could thereby reduce false-positive predictions of gut biotransformation products by reasoning over substrate-enzyme relationships and known reaction outcomes. Moreover, LLMs could enhance multi-step pathway prediction by inferring complete biotransformation routes from incomplete or partially annotated data and filtering implausible reactions that arise from data sparsity and model hallucination.

## Supplementary Material

Supplementary Material Clean_revised_supplementary_file.docx

## Data Availability

The datasets used in this study (BRD, MetaNetX, the 10 partitions, PP, and EP), together with the scripts for running the two pipelines for fine-tuning the 10 folds and predicting the PP dataset using SRP and MRP, respectively, are publicly available at: https://git.ufz.de/masun/GUTchetp/. A demo version of the GUTchetp model for gut biotransformation product prediction is also publicly available. Two models, M4 and M31, are available to be used with the demo. The remaining trained models and additional materials supporting the findings of this study are available from the corresponding author upon request. A reproducibility manifest summarizing the code version, random seed(s), data partition identifiers, and model availability is provided in the Supplementary Information (Supplementary Table S21).

## Web server

We have developed a user-friendly web server that is freely accessible on the AI-CHEMBIOME website (https://web.app.ufz.de/ai-chembiome/). This platform allows users, through “GUTchetp” menu, to input the candidate chemical compound and then predict a list of gut biotransformation products sorted according to the most-frequent prediction or gut reaction similarity. By selecting any predicted biotransformation product, a list of possible gut reactions is depicted with the possibility to display their associated enzymes and species. Additionally, the user can select which specify metabolic context (Gut Microbes and Human, Human, or All) to specify for predictions. Through “GUT GENOMES” menu, HGRESC is fully accessible and searchable. All site options support data download. The website was constructed on the basis of the packages of Streamlit, Python 3, Pandas, and Numpy.
