# Supplementary material for: GUTchetp: integrated prediction of gut microbial biotransformation profiles using ensemble monolingual and multilingual neural machine translation and enzyme class consistency–reaction similarity
Source: Gut Microbes. 2026 Sep 12;18(1):2726639. doi: 10.1080/19490976.2026.2726639 (PMC13577295; doi:10.1080/19490976.2026.2726639)
Supplement: Supplementary file 1 [file KGMI_A_2726639_SM2483.docx]

**Supplementary Information**

**GUTchetp: Integrated Prediction of Gut Microbial Biotransformation Profiles using Ensemble Monolingual and Multilingual Neural Machine Translation and Enzyme Class Consistency–Reaction Similarity**

Masun Nabhan Homsi^a*^, Martin von Bergen^a,b,c^

^a^Department of Molecular Toxicology, Helmholtz Centre for Environmental Research (UFZ), Leipzig, Germany

^b^Institute of Biochemistry, Leipzig University, Leipzig, Germany

^c^German Centre for Integrative Biodiversity Research (iDiv) Halle-Jena-Leipzig, Puschstraße, 4, 04103 Leipzig, Germany

**List of tables**

**Supplementary Table S1.** Characteristics of the metabolic reaction databases considered in this study.

**Supplementary Table S2.** Reproducibility of benchmark setup for preprocessing and post-processing steps.

**Supplementary Table S3**. Data format for multi-representation neural machine translation.

**Supplementary Table S4.** Pseudocode of the best checkpoint selection procedure.

**Supplementary Table S5.** Pseudocode for the model selection agent.

**Supplementary Table S6**. Reactions with similarity >=95% and ≤98%.

**Supplementary Table S7.** Overview of dataset statistics, including raw and cleaned reaction counts, data splits, and usage in the GUTchetp component.

**Supplementary Table S8.** List of functional group codes used in Figure 2(N).

**Supplementary Table S9.** Transformer architecture and parameters used for both pretraining, fin-tuning and mixed-tuning, and translating.

**Supplementary Table S10.** Model configurations used to build the base models for GUTchetp

**Supplementary Table S11**. Winning base model configurations across data partitions (bold MRP models), showing the candidate ordering.

**Supplementary Table S12.** Interpretation of the color shading, effect size categories, and statistical significance annotations used throughout this study in all figures displaying statistical test results for BLEU scores and MaxFrag accuracy.

**Supplementary Table S13.** Comparison of GUTchetp with other tools (MetaTrans and MetaPredictor)

**Supplementary Table S14.** Statistical significance (Sig.) test results comparing MetaTrans and MetaPredictor versus GUTchetp for BLEU scores using a one-tailed Wilcoxon signed-rank test (W) across Top-1 to Top-20 predictions.

**Supplementary Table S15.** Statistical significance (Sig.) test results comparing MetaTrans and MetaPredictor versus GUTchetp for MaxFrag accuracy using a one-tailed McNemar’s test across Top-1 to Top-20 predictions.

**Supplementary Table S16.** Evaluation of fingerprints combinations for first-, second-, and third-level EC number prediction using random forest classifiers.

**Supplementary Table S17.** Distribution of enzymatic reactions across 69 distinct EC level-2 categories

**Supplementary Table S18.** Model-specific margin thresholds defining High-confidence, Suggestive, and Low-confidence predictions tiers for RF1 and RF2.

**Supplementary Table S19.** Results of the one-tailed McNemar’s test for statistical significance (Sig.) comparing GUTchetp with the Reaction Similarity method and the SIMMER tool across Top-1 to Top-20 prediction.

**Supplementary Table S20.** Comparison of Top-20 BLEU (%) scores for 160 chemical and structural features between GUTchetp and other tools (MetaPredictor and MetaTrans).

**Supplementary Table S21.** Reproducibility manifest for GUTchetp.

**List of figures**

**Supplementary Figure S1.** Data integration workflow used to construct the BRD and MetaNetX datasets.

**Supplementary Figure S2.** Data cleaning workflow.

**Supplementary Figure S3.** Pairwise shared reaction counts across datasets after data standardization, integration and cleaning.

**Supplementary Figure S4.** Data exploratory.

**Supplementary Figure S5.** Variations in molecular properties between HGBR and test dataset (PP).

**Supplementary Figure S6**. Distribution of enzymatic reactions by first-level and second-level EC numbers in the EP dataset.

**Supplementary Figure S7.** EC number coverage in the HGRESC.

**Supplementary Figure S8.** Performance of the first GUTchetp component based on the MaxFrag accuracy metric.

**Supplementary Figure S9**. Absolute differences in BLEU scores between one-step and two-step fine-tuning.

**Supplementary Figure S10**. BLEU scores for 120 base models.

**Supplementary Figure S11.** MaxFrag accuracy for 120 base models.

**Supplementary Figure S12**. Encoder self-attention distribution for atoms generated during molecular translation between the reactant (Lactulose - top) tokens and predicted product (Fructose - bottom) tokens in the fourth attention layer, averaged over eight heads.

**Supplementary Figure S13.** Statistical significance test results comparing the HGBR baseline ensemble model and A5 ensemble model across Top-1 to Top-100 predictions.

**Supplementary Figure S14.** Statistical significance test results comparing the ensemble A5 vs. Adapt5 and A10 vs. Adapt10 translation models across Top-1 to Top-100 predictions.

**Supplementary Figure S15.** Statistical significance test results comparing the ensemble Non-MFT and MFT models across Top-1 to Top-100 predictions.

**Supplementary Figure S16.** Statistical significance test results comparing the ensemble Non-MRP_MFT and MRP_MFT models across Top-1 to Top-100 predictions.

**Supplementary Figure S17**. Frequency of best-performing model configurations.

**Supplementary Figure S18**. Distribution of the 35 best base models per partition (Fold).

**Supplementary Figure S19**. Occurrence of configurations leveraging SRP (SMILES).

**Supplementary Figure S20**. Occurrence of configurations leveraging MRP.

**Supplementary Figure S21**. Performance of the 35 winning base models.

**Supplementary Figure S22**. Distribution of the number of base models added to the Basic level for 479 gut biotransformation compounds.

**Supplementary Figure S23**. Computational time required by each prediction level to predict 497 gut biotransformation products (without augmentation).

**Supplementary Figure S24.** MaxFrag accuracy for hybrid ensemble performance across four prediction levels.

**Supplementary Figure S25.** Statistical significance test results comparing the BLEU scores of the 12 ensemble configurations and GUTchetp (Exhaustive level) across Top-1 to Top-100 predictions.

**Supplementary Figure S26.** Statistical significance test results comparing the MaxFrag accuracy of the 12 ensemble configurations and GUTchetp (Exhaustive level) across Top-1 to Top-100 predictions.

**Supplementary Figure S27.** A10MFT–GUTchetp overlap and differences prediction by challenging-case criteria at Top-20 and Top-200.

**Supplementary Figure S28.** Benchmarking GUTchetp against MetaTrans and MetaPredictor using MaxFrag accuracy for biotransformation product predictions.

**Supplementary Figure S29**. Comparison of metric scores between GUTchetp and other tools on the test dataset (PP).

**Supplementary Figure S30.** Comparative performance analysis of 31 models trained using different combinations of five fingerprint functions compared with the CLAIRE model.

**Supplementary Figure S31**. F1-scores across third-level of EC number subclasses in the EP dataset.

**Supplementary Figure S32.** Coverage of each confidence tier across candidate target-accuracy pairs for the RF1 model.

**Supplementary Figure S33.** Coverage of each confidence tier across candidate target-accuracy pairs for the RF2 model.

**Supplementary Figure S34.** Performance comparison of GUTchetp with reaction similarity method (A), and SIMMER (B) on the EP dataset (95 reactions).

**Supplementary Figure S35.** Statistical significance test results comparing the Reaction Similarity method and GUTchetp across Top-1 to Top-10 predictions.

**Supplementary Figure S36.** Statistical significance test results comparing SIMMER tool versus GUTchetp across Top-1 to Top-10 predictions.

**Supplementary Figure S37**. Overall Top-N accuracy for predicting gut microbial biotransformation profiles using the 95 compounds.

**Supplementary Figure S38**. Predicted bacteria genera involved in the biotransformation of Methotrexate to DAMPA.

**Supplementary Figure S39**. Predicted bacteria genera involved in the biotransformation of stevioside to steviol.

**Supplementary Figure S40**. Predicted bacteria genera involved in the biotransformation of DDT to DDD.

**Supplementary Figure S41**. Predicted bacteria genera involved in the biotransformation of diclofenac glucuronide to diclofenac.

**Supplementary Figure S42**. BLEU scores across compound-biotransformation products similarity bins.

**Supplementary Figure S43**. BLEU scores across transformation ratio bins.

**Supplementary Figure S44**. BLEU scores across mass changes bins.

**Supplementary Figure S45**. BLEU scores across compound atom-count bins.

**Supplementary Figure S46**. BLEU scores across compound SMILES-length bins.

**Supplementary Figure S47**. BLEU scores across chiral, ring-opening and ring-forming transformations.

**Supplementary Figure S48**. BLEU scores across 49 functional groups**.**

**Supplementary Figure S1. Data integration workflow used to construct the BRD and MetaNetX datasets.** Data cleaning workflow in Supplementary Figure S2.

**Supplementary Table S1. Characteristics of the metabolic reaction databases considered in this study.**

| **Dataset** | **Version** | **Type of dataset** | **Number of reactions** | **Last Access** |
| --- | --- | --- | --- | --- |
| USPTO | USPTO-Full | Organic | 1.1 M | June 2024 |
| SIMMER | - | Gut | 8,914 | June 2024 |
| MetaTrans | - | Gut and Human | 11,670 | June 2024 |
| MicrobeFDT | - | Gut and Human | 2,062 enzymes mapped with 2,062 reactions from KEGG dataset | June 2024 |
| UHGG | 2.0 | Gut | 3,686 | January 2025 |
| MetaNetX | 4.4 | Metabolite | 74,614 | June 2024 |
| Metacyc | 28.3 | Metabolite | 14,737 | June 2024 |
| KEGG | 101 | Metabolite | 11,666 | June 2024 |

**Supplementary Figure S2. Data cleaning workflow.**

**Supplementary Table S2. Reproducibility of benchmark setup for preprocessing and post-processing steps.**

| **Setting** | **Procedure** | **Preprocessing**  **Yes/No, Step** | **Postprocessing** |
| --- | --- | --- | --- |
| SMILES canonicalization | Compound and predicted metabolites were canonicalized using RDKit | Yes, data cleaning | Yes |
| Invalid SMILES filter | Removed if RDKit parsing failed | Yes, data cleaning | Yes |
| SMILES standardization | Compounds were standardized using RDKit as follows:   - Select the parent molecule - Remove formal charges - enumerate all the tautomers | Yes, data cleaning | Yes |
| Heavy atoms filter | Compounds or predicted metabolites with more than 100 heavy atoms were removed | Yes, data cleaning | Yes |
| Compound-metabolite similarity filter | Compound-metabolite pairs with a similarity below 5% were removed | Yes, data cleaning | No |
| Transformation ratio filter | Metabolites comprising less than 10% of the parent compound’s atom count were removed | Yes, data cleaning | No |
| SMILES tokenization | Compound SMILES strings were tokenized at the atoms level | Yes, data tokenization | No |
| Atom-type filter | Atoms not present in the parent compound and not among the common organic elements (C, O, H, S, P, N) were removed | No | Yes |
| SMILES randomization | Compounds were randomized using RDKit | Yes, data augmentation | No |
| General atom (*) | Remove general atom from both parent compound and metabolite | Yes, data augmentation for MRP | No |
| Detokenization | Convert the tokenized prediction back to a molecular representation by removing spaces between atoms | No | Yes |
| Reranking | Re-rank predicted SMILES candidates | No | Yes |

**Supplementary Table S3. Data format for multi-representation neural machine translation.** Tags (tokens) were added to the beginning of each source sequence (compound/substrates) to indicate the target chemical representation (product) to be predicted by the transformers. Each reaction in the fine-tuning datasets was augmented 9 times.

| Tag | Representation source | → | Representation target | Acronym |
| --- | --- | --- | --- | --- |
| <SM> | SMILES | → | SMILES | SM2SM |
| <SM> | InChI | → | SMILES | CH2SM |
| <SM> | SELFIES | → | SMILES | SF2SM |
| <CH> | InChI | → | InChI | CH2CH |
| <CH> | SMILES | → | InChI | SM2CH |
| <CH> | SELFIES | → | InChI | SF2CH |
| <SF> | SELFIES | → | SELFIES | SF2SF |
| <SF> | SMILES | → | SELFIES | SM2SF |
| <SF> | InChI | → | SELFIES | CH2SF |

**Supplementary Table S4. Pseudocode of the best checkpoint selection procedure.**

| **Input:**  **Output:**  **Procedure:** | - A set of checkpoints:$C=\left\{ c_{i} \right\}_{i=1}^{N}$ , N is the number of checkpoint models generated during fine-tuning process - The PP testing dataset: $D=\left\{ d_{j} \right\}_{j=1}^{M}$ , M is the number of compounds - For each checkpoint c_i_, a set of correctly predicted compounds $P_{i}\subseteq D$ - Optimal subset of checkpoints $C^{*}\subseteq C$ - Final selected compound set $FSC\subseteq D$ - Best accuracy $\alpha^{*}\in\left[ 0, 1 \right]$      1. for each checkpoints *i* =1 to *N*:    - compute $P_{i}\subseteq D,$correctly predicted compounds for checkpoint C_i_   end   2. Sort checkpoints {C_i_} in descending order according to the number of correctly predicted compounds \|P_i_\| 3. Initialize: FSC←∅, C_selected_←∅, α^*^ ←∅ 4. for each $c_{i}\in C$ :    - $\Delta_{i}=P_{i} - FSC$    - if $\Delta_{i}\neq$∅:      - $FSC \leftarrow FSC \cup P_{i}$      - $C_{selected}\leftarrow C_{selected} \cup\left\{ c_{i} \right\}$      - Compute accuracy $\alpha\leftarrow\frac{\left\vert FSC \right\vert}{\left\vert D \right\vert}$      - if α > α^*^ then:        - α^*^ ← α, C^*^← C_selected_      - end    - end   end   5. Return C^*^, α^*^, FSC |
| --- | --- |

**Supplementary Table S5. Pseudocode for the model selection agent.**

| **Part I: Model training** | | |
| --- | --- | --- |
|  | **Input**:   - A training set $\mathcal{T=}\{\left( m_{i},a_{i} \right){\}}_{i=1}^{N}$ - $m_{i}:$a molecule represented as a SMILES string - $a_{i}\mathcal{\in A}$ : the partition labels - $\phi\mathcal{:M}\to\{0,1{\}}^{d}$: the Morgan fingerprint function - $d=1024$ : fingerprint vectors length - $T\in N$ : number of trees - $L\in N:$ leaf size threshold for tree nodes   **Output**:   - A forest of trees $\mathcal{F=}\{\mathcal{T}_{1},\ldots,\mathcal{T}_{t}\}$ - Association of fingerprint indices with partition identifier $\text{C2A}:\{0,\ldots,N-1\}\to2^{\mathcal{A}}$ - Mapping between partition names and internal IDs:   - PartitionName_ID: $\mathcal{A}\mathbb{\to N}$   - PartitionID_Name: $\mathbb{N\to}\mathcal{A}$   **Procedure**: | |
| 1. | for each $i=1 to N$: | |
|  | - Calculate the fingerprint vector $v_{i}=\phi\left( m_{i} \right)\in\{{0,1\}}^{d}$for the molecule $m_{i}$ | |
|  | - Assign a unique label ID to $a_{i}$ | |
|  | - Index the pair $\left( v_{i},\mathrm{ID}\left( a_{i} \right) \right)$ as $i$ | |
|  | - Add partition $ID\left( a_{i} \right)$ to $C2A\left[ i \right]$ | |
|  | end | |
| 2. | Let $\mathcal{D}={\{v}_{1},\ldots,v_{N}\}$ be the set of fingerprint vectors | |
| 3. | For each $t=1 to T$, build a tree $\mathcal{T}_{t}$ over $\mathcal{D}$ using the following recursive procedure: | |
|  |  | **Tree construction steps:** |
|  |  | Given a subset of $D^{'}\subseteq D$: |
|  |  | a. If $\mid D^{'}\mid\leq L$, return a leaf node containing $D^{'}$ |
|  |  | b. Otherwise: |
|  |  | - Select randomly two distinct vectors $v_{a},v_{b}\in D^{'}$ |
|  |  | - Compute $u=\frac{v_{a}-v_{b}}{\vert v_{a}-v_{b}\vert}$ |
|  |  | - For each $v\in\mathcal{D}^{'}$ calculate $p_{v}=\left\langle v,u \right\rangle$ |
|  |  | - Determine the median $\mu$ of $p_{v}\mid v\in D'\}$ |
|  |  | - Partition $\mathcal{D}^{'}$ into:   - $D_{\mathrm{left}}=\{v\in\mathcal{D}^{'}\mid p_{v}\leq\mu\}$   - $D_{right}=\{v\in\mathcal{D}^{'}\mid p_{v}>\mu\}$ - Build left and right subtrees recursively from $D_{\mathrm{left}}$and $D_{right}$, respectively |
|  | end | - Return an internal node, store $u,\mu$ and link to its children.   end |

| **Part II: Query and model selection** | |
| --- | --- |
|  | **Input**:   - A query molecule $m_{q}\mathcal{\in M}$ - The trained tree forest $\mathcal{F}$ - The compound partition map C2A - The partition ID-to-name mapping PartitionID_Name - A similarity function $\text{Sim}:\mathbb{R}^{d}\times\mathbb{R}^{d}\mathbb{\to R}$ - Thresholds:   - $k$: number of nearest neighbors to retrieve   - $K$: number of models to return   **Output**:   - A ranked list of $K$ partitions $\left( a_{i},s_{i} \right)_{i=1}^{K}$ where s_i_ is a similarity-based vote score   **Procedure**: |
| 1. | Calculate the fingerprint vector of the query $\mathbf{v}_{q}=\phi\left( m_{q} \right)\in\mathbb{R}^{d}$ |
| 2. | Initialize a candidate set $\mathcal{C\subseteq}\{0,\ldots,N-1\}$ to store potential neighbors |
| 3. | For each tree $\mathcal{T}_{t}\mathcal{\in F}$: |
|  | - Traverse the tree from the root: |
|  | - At each internal node with direction vector $u$ and threshold $\mu$, calculate $p_{q}=\left\langle v_{q},u \right\rangle$ and proceed to the left or right child depending on whether $p_{q}\leq\mu$ - When reaching a leaf node, collect its stored vector indices and add them to $\mathcal{C}$   end |
| 4. | For each index $i\mathcal{\in C}$, calculate the similarity score: $s_{i}=\text{Sim}\left( \mathbf{v}_{\mathbf{q}},\mathbf{v}_{\mathbf{i}} \right)$ end |
| 5. | Initialize a partition score map $S\mathcal{:A}\mathbb{\to R}$ |
| 6. | For each $i\mathcal{\in C}$ and for each partition ID $a\in\text{C2A}\left[ i \right]$, update $S\left[ a \right]\leftarrow S\left[ a \right]+s_{i}$ end |
| 7. | Sort the partitions $a\mathcal{\in A}$ by decreasing score $S\left[ a \right]$ |
| 8. | Return the top $K$ partitions as $\{\left( \text{PartitionID\_Name}\left( a_{i} \right),S\left[ a_{i} \right] \right){\}}_{i=1}^{K}$ |
| 9. | Select those models that were trained by the selected partitions and add them to the Base model. |

**Supplementary Figure S3. Pairwise shared reaction counts across datasets after data standardization, integration and cleaning.** Diagonal entries represent the total number of reactions contributed by each dataset, while off-diagonal entries indicate the number of reactions shared between pairs of datasets.

**Supplementary Figure S4. Data exploratory:** (A) Proportion of reactions assigned EC numbers multi-label per level. (B) Proportion of reactions assigned EC numbers multi-label per level, and dataset. (C) PCA of the most similar reactions (>=95%) between fine-tuning (BRD, MetaNetX, (Fx)) and test datasets (Tx). Supplementary Table S6 details theses reactions. (D) Reactions similarity between test dataset (PP) and MetaNetX.

**Supplementary Table S6**. **Reactions with similarity >=95% and ≤98%.** Colors to distinguish reaction compounds.

| **Similar Reactions** | **First reaction** | **Second reaction** | **Third reaction** | **Comments** |
| --- | --- | --- | --- | --- |
| Case 1:  T4 and T5 | Ginsenoside Rb1>>Ginsenoside compound K | Ginsenoside Rc>>Ginsenoside compound K | - | Share the same product, but the reactants are different (red) |
| Case 2:  F4 and T3 | (3aR,7aS)-1-[2-[(1-ethoxy-1-oxo-4-phenylbutan-2-yl)amino]propanoyl]-2,3,3a,4,5,6,7,7a-octahydroindole-2-carboxylic acid () **>>** (3aR,7aS)-1-[2-[(1-carboxy-3-phenylpropyl)amino]propanoyl]-2,3,3a,4,5,6,7,7a-octahydroindole-2-carboxylic acid | Ramipril**>>**Ramiprilat |  | Completely different reactions, but highly similar |
| Case 3:  F2 and F3 | MNXR118802 (F2)  D-glucose + (20S)-protopanaxadiol <=>  H2O+ (20S)-ginsenoside Rh2 | MNXR171020 (F3) (R12383)  UDP + (20S)-ginsenoside Rh2 + H(+)  <=>  UDP-alpha-D-glucose + (20S)-protopanaxadiol  Enzymes  2.4.1.-  2.4.1.364 | - | Different co-factors (green), MNXR118802  is not an enzymatic reaction |
| Case 4:  F5, F6 and F7 | MNXR118793(F5)  ginsenoside Mc + D-glucose <=>  H2O+(20S)-ginsenoside Rc  Enzymes:  3.2.1.-  3.2.1.191 | MNXR118804= MNXR196817 (F6)  D-glucose +(20S)-ginsenoside C-K  <=>  H2O + (20S)-ginsenoside Rd  Enzymes: 3.2.1.-  3.2.1.191 | MNXR118805(F6)  D-glucose +gypenoside LXXV  <=>  H2O+(20S)-ginsenoside Rb1  Enzymes: 3.2.1.191 | Share the same product, but different reactants (red) |

**Supplementary Figure S5. Variations in molecular properties between HGBR and test dataset (PP).** Comparison of the distribution of six key physicochemical and structural descriptors between HGBR (red) and PP (blue) datasets (a-f).

**Supplementary Figure S6. Distribution of enzymatic reactions by first-level (A) and second-level (B) EC numbers in the EP dataset.**

**Supplementary Table S7.** **Overview of dataset statistics, including raw and cleaned reaction counts, data splits, and usage in the GUTchetp component.**

| **Dataset** | **Raw size**  **reactions** | **After cleaning**  **reactions** | **Split (Fine-tuning/training, validation, Test)** | **Used in GUTchetp component** |
| --- | --- | --- | --- | --- |
| BRD | 26,332 | 25,393 | 90% fine-tuning and 10% validation | Gut biotransformation products |
| MetaNetX | 74,614 | 25,125 | 90% for fine-tuning and 10% validation | Gut biotransformation products |
| PP | 479 | 479 | Test | Gut biotransformation products |
| CLAIRE | 166918 and 18,816 | - | 16,6918 training and 18,816 Test | Enzyme prediction |
| HGBR | 6,730 | 6,730 | Training two random forests | Enzyme prediction |
| EP | 95 | 95 | Test | Enzyme prediction |

**Supplementary Table S8. List of functional group codes used in Figure 2(N).**

| **Functional group name** | **FG(n)** | **Functional group name** | **FG(n)** |
| --- | --- | --- | --- |
| Bicyclic Compounds | FG1 | Carbonyl Oxygen Excluding | FG26 |
| Aromatic Carboxylic Acids | FG2 | Carboxylic Acids | FG27 |
| Pyrrole Nitrogens with Hydrogen | FG3 | Cyclic Esters (Lactones) | FG28 |
| Imines | FG4 | Esters | FG29 |
| Nitrogen Functional Groups Attached to Aromatics | FG5 | Ether Oxygens Including Phenoxy | FG30 |
| Primary Amines | FG6 | Halogens | FG31 |
| Secondary Amines | FG7 | Hydroxylamine Groups | FG32 |
| Tertiary Amines | FG8 | Imidazole Rings | FG33 |
| XCCNR Groups | FG9 | Ketones | FG34 |
| Aliphatic Carboxylic Acids | FG10 | Ketones Excluding Diaryl Alpha-Beta Unsaturated Dienones and Heteroatom on C-alpha | FG35 |
| Aliphatic Hydroxyl Groups | FG11 | Methoxy Groups (OCH3) | FG36 |
| Aliphatic Hydroxyl Groups Excluding Tertiary OH | FG12 | Nitro Benzene Ring Substituents | FG37 |
| Alkyl Halides | FG13 | Nitro Groups | FG38 |
| Allylic Oxidation Sites Excluding Steroid Dienone | FG14 | Non-Ortho Nitro Benzene Ring Substituents | FG39 |
| Amides | FG15 | Para Hydroxylation Sites | FG40 |
| Amidine Groups | FG16 | Phenolic OH Excluding Ortho Intramolecular Hydrogen Bond Substituents | FG41 |
| Anilines | FG17 | Phenols | FG42 |
| Aromatic Amines | FG18 | Piperidine Rings | FG43 |
| Aromatic Hydroxyl Groups | FG19 | Pyridine Rings | FG44 |
| Aromatic Nitrogens | FG20 | Quaternary Nitrogen | FG45 |
| Aryl Methyl Sites for Hydroxylation | FG21 | Sulfonamides | FG46 |
| Azo Groups | FG22 | Tertiary Alicyclic Amines with No Heteroatoms (Not Quinine-like Bridged N) | FG47 |
| Benzene Rings | FG23 | Thioethers | FG48 |
| Benzodiazepines with No Additional Fused Rings | FG24 | Unbranched Alkanes of at Least 4 Members Excluding Halogenated Alkanes | FG49 |
| Carbonyl Oxygen | FG25 |  |  |

**Supplementary Figure S7. EC number coverage in the** **HGRESC**. (A) Overall coverage and missing rates of unique EC numbers relative to the HGBR dataset. (B-H) Coverage and missing rates across the seven first-level EC classes (EC:1 – EC: 7).

**Supplementary Table S9. Transformer architecture and parameters used for both pretraining, fin-tuning and mixed-tuning, and translating.**

| **Parameter name** | **Value** |
| --- | --- |
| **Pretraining, fine-tuning and mixed-tuning** | |
| Number of parameters | 20047792 |
| Encoder and decoder layers | 6 |
| Attention heads | 8 |
| Embedding size for SRP | 512 |
| Embedding size for MRP | 483 |
| Dimension of feedforward layer | 2048 |
| Cross-entropy loss with label smoothing rate | 0.0 |
| Training batch size | 6144 tokens |
| Validation batch size | 2048 tokens |
| Optimizer | Adam |
| Adam β1 | 0.9 |
| Adam β2 | 0.998 |
| Warm-up steps | 8000 |
| Initial learning rate | 2.0 |
| Dropout rate | 0.1 |
| Sharing embedding between reactants and products | Yes |
| **Predicting (Translating)** | |
| n-Best, n-Beam | 20/200 |
| Maximum length of characters (SRP) | 150 |
| Maximum length of characters (MRP) | 650 |
| Minimum length of characters (MRP & SRP) | 5 |
| Batch size (MRP) | 1024 |
| Batch size (SRP) | 8000 |
| Batch type | Tokens |
| Seed | 0 |
| Replace unknown (replace_unk) | True |

**Supplementary Figure S8. Performance of the first GUTchetp component based on the MaxFrag accuracy metric.** (A) Impact of data size. (B) Impact of adaptive augmentation. (C) Impact of MFT. (D) Impact of multi-representation molecular translation. (E) Performance of ensemble models across 12 different configurations.

**Supplementary Figure S9. Absolute differences in BLEU scores between one-step and two-step fine-tuning.**

**Supplementary Table S10. Model configurations used to build the base models for GUTchetp.**

| **Configuration**  **#** | **Model**  **Acronym** | **Model Name** | **Configuration Details** |
| --- | --- | --- | --- |
| C1 | A5 | TL1-USPTO-BRD-SRP-Aug5 | - One-step transfer learning. - Fine-tuning USPTO model on BRD dataset - Single Representation: SMILE - Augmentation: 5 times |
| C2 | A5MFT | TL2-MFT-USPTO- METANETX -BRD-SRP-Aug5 | - Two-steps of transfer learning. - Fine-tuning USPTO model on METANETX dataset - Mixed-finetuning on BRD, METANETX and USPTO datasets - Single Representation: SMILE - Augmentation: 5 times |
| C3 | A10 | TL1-USPTO-BRD-SRP-Aug10 | - One-step transfer learning. - Fine-tuning USPTO model on BRD dataset - Single Representation: SMILE - Augmentation: 10 times |
| C4 | A10MFT | TL2-MFT-USPTO- METANETX-BRD -SRP-Aug10 | - Two-steps of transfer learning. - Fine-tuning USPTO model on METANETX dataset - Mixed-finetuning on BRD, METANETX and USPTO datasets - Single Representation: SMILE - Augmentation: 10 Times |
| C5 | Adapt5 | TL1-USPTO-BRD-SRP-Aug5-10 | - One-step transfer learning. - Fine-tuning USPTO model on BRD dataset - Single Representation: SMILE - Adaptive Augmentation: Reactions with less than 25% similarity between the reactant and product were augmented 10 times; otherwise, they were augmented 5 times. |
| C6 | Adapt5MFT | TL2-MF-USPTO- METANETX-HGBR-SR-Aug5-10 | - Two-steps of transfer learning. - Fine-tuning USPTO model on METANETX - Mixed-finetuning on BRD, METANETX and USPTO datasets - Single Representation: SMILE - Adaptive Augmentation: Reactions with less than 25% similarity between the reactant and product were augmented 10 times; otherwise, they were augmented 5 times. |
| C7 | Adapt10 | TL1-USPTO-BRD-SRP-Aug10-20 | - One-step transfer learning. - Fine-tuning USPTO model on BRD dataset - Single Representation: SMILE - Adaptive Augmentation: Reactions with less than 25% similarity between the reactant and product were augmented 20 times; otherwise, they were augmented 10 times. |
| C8 | Adapt10MFT | TL2-MFT-USPTO-METANETX-BRD-SRP-Aug10-20 | - Two-steps of transfer learning. - Fine-tuning USPTO model on METANETX dataset - Mixed-finetuning on BRD and METANETX and USPTO datasets - Single Representation: SMILE - Adaptive Augmentation: Reactions with less than 25% similarity between the reactant and product were augmented 20 times; otherwise, they were augmented 10 times. |
| C9 | Adapt5MRP | TL1-USPTO-BRD-MRP-Aug5-10 | - One-step transfer learning. - Fine-tuning USPTO on BRD dataset - Multiple Representation: SMILE, InChI and SELFIES - Adaptive Augmentation: Reactions with less than 25% similarity between the reactant and product were augmented 10 times; otherwise, they were augmented 5 times. |
| C10 | Adapt5MRP_MFT | TL2-MFT-USPTO-BRD-METNETX-MRP-Aug5-10 | - Two-steps of transfer learning. - Fine-tuning USPTO model on METANETX dataset - Mixed-finetuning on BRD, METANETX and USPTO datasets - Multiple Representation: SMILE, InChI and SELFIES - Adaptive Augmentation: Reactions with less than 25% similarity between the reactant and product were augmented 10 times; otherwise, they were augmented 5 times. |
| C11 | Adapt10MRP | TL1-USPTO-BRD-MRP-Aug10-20 | - One-step transfer learning - Fine-tuning USPTO model on BRD dataset - Multiple Representation: SMILE, InChI and SELFIES - Adaptive Augmentation: Reactions with less than 25% similarity between the reactant and product were augmented 20 times; otherwise, they were augmented 10 times. |
| C12 | Adapt10MRP_MFT | TL2-MFT-USPTO-BRD-METNETX-MRP-Aug10-20 | - Two-steps of transfer learning - Fine-tuning USPTO model on METANETX dataset - Mixed-finetuning on BRD, METANETX and USPTO datasets - Multiple Representation: SMILE, InChI and SELFIES - Adaptive Augmentation: Reactions with less than 25% similarity between the reactant and product were augmented 20 times; otherwise, they were augmented 10 times. |

**Supplementary Table S11. Winning base model configurations across data partitions (bold MRP models), showing the candidate ordering.**

| Model | Configuration | Fold  (Partition) | Model | Configuration | Fold  (Partition) |
| --- | --- | --- | --- | --- | --- |
| M1 | C8 | 8 | M19 | C5 | 3 |
| M2 | C2 | 5 | M20 | C1 | 10 |
| M3 | C8 | 1 | M21 | **C10** | 6 |
| M4 | **C11** | 2 | M22 | **C9** | 6 |
| M5 | C8 | 3 | M23 | **C12** | 8 |
| M6 | C8 | 10 | M24 | **C12** | 4 |
| M7 | **C9** | 2 | M25 | C2 | 10 |
| M8 | C6 | 2 | M26 | **C12** | 2 |
| M9 | **C12** | 3 | M27 | C7 | 9 |
| M10 | C8 | 5 | M28 | C2 | 9 |
| M11 | C5 | 4 | M29 | C5 | 9 |
| M12 | C6 | 10 | M30 | C4 | 9 |
| M13 | C4 | 4 | M31 | C4 | 7 |
| M14 | C6 | 3 | M32 | C4 | 6 |
| M15 | C5 | 2 | M33 | C7 | 2 |
| M16 | C4 | 2 | M34 | **C9** | 4 |
| M17 | C2 | 1 | M35 | C2 | 4 |
| M18 | C4 | 3 |  |  |  |

**Supplementary Figure S10. BLEU scores for 120 base models.**

**Supplementary Figure S11. MaxFrag accuracy for 120 base models.**

**Supplementary Figure S12. Encoder** **self-attention distribution for atoms generated during molecular translation between the reactant (Lactulose - top) tokens and predicted product (Fructose - bottom) tokens in the fourth attention layer, averaged over eight heads.** (A) CH2SF, (B) SM2CH, (C) CH2CH and (D) SM2SM**.**

**Supplementary Figure S13. Statistical significance test results comparing the HGBR baseline ensemble model and A5 ensemble model across Top-1 to Top-100 predictions.** (A) BLEU scores (mean ±95% CI) for the HGBR (Model-A) and A5 (Model-B) ensemble models. Details of the color scheme and panel-specific annotations are provided in Supplementary Table S12.

**Supplementary Table S12. Interpretation of the color shading, effect size categories, and statistical significance annotations used throughout this study in all figures displaying statistical test results for BLEU scores and MaxFrag accuracy.**

| **Panel** | **Visual element** | **Meaning** | **Criterion / Threshold** |
| --- | --- | --- | --- |
| **Top panel**  **(BLEU)** | Orange shading | B significantly outperforms A | One-tailed Wilcoxon  signed-rank test, *p* < 0.05 |
| **Lower panel**  **(BLEU)** | Light green shading | Medium effect size | \|RBC\| ≥ 0.3 |
|  | Light yellow shading | Large effect size | \|RBC\| ≥ 0.5 |
|  | White / unshaded | Negligible effect size | \|RBC\| < 0.3 |
| **Top panel**  **(MaxFrag accuracy/**  **Accuracy)** | Orange shading | B significantly outperforms A | One-tailed McNemar's test,  *p* < 0.05 |
| **Lower panel (MaxFrag accuracy/**  **Accuracy)** | Solid gray line | No effect | OR = 1 (log scale) |
|  | Yellow shading | Large effect favoring B | OR < 0.33 |
|  | Light green shading | Medium-to-large effect favoring B | 0.33 ≤ OR < 0.5 |
|  | White shading | Small or negligible effect | 0.5 ≤ OR ≤ 2.0 |
|  | Light blue shading | Medium-to-large effect favoring A | 2.0 < OR ≤ 3.0 |
|  | Plum shading | Large effect favoring A | OR > 3.0 |
| **All panels** | * (green) | Statistically significant | *p* < 0.05 |
|  | ** (violet) | Highly significant | *p* < 0.01 |
|  | *** (orange) | Very highly significant | *p* < 0.001 |
|  | **** (blue) | Extremely significant | *p* < 0.0001 |

**Supplementary Figure S14. Statistical significance test results comparing the ensemble A5 vs. Adapt5 and A10 vs. Adapt10 translation models across Top-1 to Top-100 predictions.** All values are presented as mean ±95% CI. (A-B) BLEU scores and MaxFrag accuracy for the A5 (Model-A) and Adapt5 (Model-B) models, respectively. (C-D) BLEU scores and MaxFrag accuracy for the A10 (Model-A) and Adapt10 (Model-B) models, respectively. As the results from Top-100 to Top-200 were identical, they were omitted. Details of the color scheme and panel-specific annotations are provided in Supplementary Table S12.

**Supplementary Figure S15. Statistical significance test results comparing the ensemble Non-MFT and MFT models across Top-1 to Top-100 predictions**. All values are presented as mean ±95% CI. (A, C, E, and G) BLEU scores and (B, D, F, and H) MaxFrag accuracy for the A5 versus A5MFT, A10 versus A10MFT, Adapt5 versus Adapt5MFT, and Adapt10 versus Adapt10MFT (A vs. B), respectively. As the results from Top-100 to Top-200 were identical, they were omitted. Details of the color scheme and panel-specific annotations are provided in Supplementary Table S12.

**Supplementary Figure S16. Statistical significance test results comparing the ensemble Non-MRP_MFT and MRP_MFT models across Top-1 to Top-100 predictions**. All values are presented as mean ±95% CI. (A and C) BLEU scores and (B and D) MaxFrag accuracy for the Adap5MRP versus Adap5MRP_MFT and Adap10MRP versus Adap10MRP_MFT (A vs. B), respectively. As the results from Top-100 to Top-200 were identical, they were omitted. Details of the color scheme and panel-specific annotations are provided in Supplementary Table S12.

**Supplementary Figure S17. Frequency of best-performing model configurations.**

**Supplementary Figure S18. Distribution of the 35 best base models per partition (Fold).**

**Supplementary Figure S19. Occurrence of configurations leveraging SRP (SMILES).**

**Supplementary Figure S20. Occurrence of configurations leveraging MRP.**

**Supplementary Figure S21. Performance of the 35 winning base models**. The navy line shows the increase in accumulative Top-20 accuracy as additional base models are incorporated.

**Supplementary Figure S22. Distribution of the number of base models added to the Basic level for 479 gut biotransformation compounds.**

**Supplementary Figure S23. Compu4tational time required by each prediction level to predict 497 gut biotransformation products (without augmentation).**

**Supplementary Figure S24. MaxFrag accuracy for hybrid ensemble performance across four prediction levels.**

**Supplementary Figure S25. Statistical significance test results comparing the BLEU scores of the 12 ensemble configurations and GUTchetp (Exhaustive level) across Top-1 to Top-100 predictions.** As the results from Top-100 to Top-200 were identical, they were omitted. Panels (A-L) correspond to comparisons of GUTchetp against A5, A5MFT, A10, A10MFT, Adapt5, Adapt5MFT, Adapt10, Adapt10MFT, Adapt5MRP, Adapt5MRP_MFT, Adapt10MRP, and Adapt10MRP_MFT, respectively. Details of the color scheme and panel-specific annotations are provided in Supplementary Table S12.

**Supplementary Figure S26. Statistical significance test results comparing the MaxFrag accuracy of the 12 ensemble configurations and GUTchetp (Exhaustive level) across Top-1 to Top-100 predictions.** As the results from Top-100 to Top-200 were identical, they were omitted. Panels (A-L) correspond to comparisons of GUTchetp against A5, A5MFT, A10, A10MFT, Adapt5, Adapt5MFT, Adapt10, Adapt10MFT, Adapt5MRP, Adapt5MRP_MFT, Adapt10MRP, and Adapt10MRP_MFT, respectively. Details of the color scheme and panel-specific annotations are provided in Supplementary Table S12.

**Supplementary Figure S27. A10MFT–GUTchetp overlap and differences prediction by challenging-case criteria at Top-20 and Top-200.**

**Supplementary Figure S28. Benchmarking GUTchetp against MetaTrans and MetaPredictor using MaxFrag accuracy for biotransformation product predictions.**

**Supplementary Figure S29. Comparison of metric scores** **between GUTchetp and other tools on our test dataset (PP), at (A) Top-1, (B) Top-5, (C) Top-10, and (D) Top-15.**

**Supplementary Table S13. Comparison of GUTchetp with other tools (MetaTrans and MetaPredictor).**

|  | **MetaTrans** | **MetaPredictor** | **GUTchetp** |
| --- | --- | --- | --- |
| **Datasets** | | | |
| Dataset Transfer Learning | USPTO (1.1M organic reactions) | USPTO (1.1M organic reactions) | USPTO (1.1M organic reactions) +  MetaNetX (25,125 metabolic reactions) |
| Training dataset | 10427 reactions | 12692 for SoM (Site of Metabolite) and 14782 for prompt-based metabolite predictor | 25,393 BRD + 25,125 MetaNetX |
| Validation dataset | 252 drugs | 241 drugs | 10% of fine-tuning datasets (BRD and MetaNetX) |
| Testing dataset | 84 drugs with 217 metabolites | 135 drugs with 283 metabolites | 348 different chemical compounds covering 6 categories with 479 metabolites |
| **Data preprocessing** | | | |
| Chemical representation | SMILES | SMILES | SMILES single representation and Multi-representation, including SMILES, InChI and SELFIES. |
| General atoms with a star (*) symbol | Replaced with common atoms in organic chemistry (C, O, S, H, N) | - | Maintained for SRP and removed for MRP |
| SMILES canonicalization | RDKit canonical SMILES | RDKit canonical SMILES | RDKit canonical SMILES |
| Duplicate reaction filter | Yes | Yes | Yes |
| Reactant and product SMILES standardization | - | - | RDKit standardization SMILES |
| Invalid SMILES handling | Yes | Yes | Yes |
| Heavy atoms filter | Reactions with extreme imbalance of heavy atoms between compounds and metabolites. | - | Reactions that involve molecules with more than 100 heavy atoms |
| Atom number filter | Reactants or metabolites with one atom | - | Reactants or metabolites with less than five atoms |
| Reactant-metabolite similarity filter | - | - | Metabolite comprises less than 10% of the reactant size. |
| Transformation ratio filter | - | - | The transformation ratio less than 10% |
| Similarity based on the size of the maximum common substructure (MCS) | - | The atom number of MCS is less than 60% of the atoms of the parent molecule. | - |
| **Data Augmentation** | | | |
| Data augmentation | - Randomize SMILES - Check SMILES validity | - Randomize SMILES - Check SMILES validity | - Duplicate reactions that either contained multiple products or considered bidirectional   For SRP:   - Randomize SMILES - Check SMILES validity   For MRP:   - Randomize SMILES - Check SMILES validity - Convert to SELFIES and InChI - A special token (tag) was added at the beginning of each compound sequence to indicate the desired metabolite chemical representation for the predictive models |
| Adaptive augmentation | - | - | Augment reactions *n × 2* times when low structural similarity was observed between reactants and their corresponding products |
| **Transfer learning** | | | |
| Transfer learning | One step | One step | Two steps |
| Mixed-finetuning | - | - | Two steps |
| **Ensemble learning** | | | |
| Ensemble Learning | Six models trained on different parameters | Two steps of ensemble learning:   1) four models trained on different parameters to identify SoM 2) six models also trained on different parameters to predict prompt-based metabolite | Thirty-fife models trained on the same transformer parameters, but with different data partitions and configurations. |
| **Data postprocessing** | | | |
| Output representation | SMILES | SMILES | SMILES  SELFIES  InChI |
| SMILES canonicalization | RDKit canonical SMILES | RDKit canonical SMILES | RDKit canonical SMILES |
| Invalid predicted molecules handling | Yes | Yes | Yes |
| Heavy atoms filter | - | - | Predicted molecules with more than 100 heavy atoms |
| Transformation ratio filter | The transformation ratio less than 25% | The transformation ratio less than 25% | The transformation ratio less than 10% |
| Atom-type filter | Candidates containing atoms absent from the parent molecule and outside the common organic elements (C, H, O, N, P, S) were removed. | | |

**Supplementary Table S14. Statistical significance (Sig.) test results comparing MetaTrans and MetaPredictor versus GUTchetp for BLEU scores using a one-tailed Wilcoxon signed-rank test (W) across Top-1 to Top-20 predictions.** Sig.: NS, not significant p ≥ 0.05; * p < 0.05; ** p < 0.01; *** p < 0.001; **** p < 0.0001.

| **Models**  **(A vs. B)** | **Top-N** | **Model (MetaTrans/**  **MetaPredictor)**  **(A)** | | **GUTchetp**  **(B)** | | **W** | **p (B>A)** | **Sig.** | **RBC** |
| --- | --- | --- | --- | --- | --- | --- | --- | --- | --- |
|  |  | **Mean±SD**  **(%)** | **95% CI**  **Low-high**  **(%)** | **Mean±SD**  **(%)** | **95% CI**  **Low-high**  **(%)** |  |  |  |  |
| MetaTrans vs. GUTchetp | 1 | 22.56±41.3 | 18.85-26.27 | 25.88±43.69 | 21.96-29.80 | 1628 | 3.12×10^-2^ | * | -0.2 |
| MetaTrans vs. GUTchetp | 2 | 31.8±45.96 | 27.68-35.93 | 34.81±47.33 | 30.56-39.06 | 1788 | 4.92×10^-2^ | * | -0.18 |
| MetaTrans vs. GUTchetp | 3 | 37.99±47.79 | 33.7-42.28 | 41.49±48.98 | 37.09-45.89 | 2471 | 2.13×10^-2^ | * | -0.2 |
| MetaTrans vs. GUTchetp | 4 | 40.71±48.39 | 36.36-45.05 | 45.6±49.47 | 41.16-50.04 | 2323 | 4.04×10^-3^ | ** | -0.27 |
| MetaTrans vs. GUTchetp | 5 | 42.9±48.71 | 38.52-47.27 | 50.25±49.62 | 45.79-54.70 | 1991 | 7.16×10^-5^ | **** | -0.38 |
| MetaTrans vs. GUTchetp | 6 | 44.43±48.82 | 40.05-48.81 | 52.75±49.44 | 48.31-57.19 | 2147 | 2.62×10^-5^ | **** | -0.4 |
| MetaTrans vs. GUTchetp | 7 | 45.89±48.96 | 41.49-50.29 | 54.54±49.25 | 50.11-58.96 | 1683 | 4.17×10^-6^ | **** | -0.46 |
| MetaTrans vs. GUTchetp | 8 | 47.56±49.07 | 43.15-51.97 | 56.12±48.99 | 51.72-60.52 | 1493 | 3.64×10^-6^ | **** | -0.47 |
| MetaTrans vs. GUTchetp | 9 | 48.4±49.11 | 43.99-52.8 | 58.42±48.65 | 54.05-62.79 | 1303.5 | 8.65×10^-8^ | **** | -0.55 |
| MetaTrans vs. GUTchetp | 10 | 49.08±49.08 | 44.68-53.49 | 58.77±48.54 | 54.41-63.13 | 1258 | 1.37×10^-7^ | **** | -0.55 |
| MetaTrans vs. GUTchetp | 11 | 49.5±49.09 | 45.09-53.91 | 59.61±48.38 | 55.26-63.95 | 1191.5 | 4.06×10^-8^ | **** | -0.57 |
| MetaTrans vs. GUTchetp | 12 | 50.34±49.09 | 45.93-54.74 | 59.99±48.28 | 55.66-64.33 | 1315 | 2.13×10^-7^ | **** | -0.54 |
| MetaTrans vs. GUTchetp | 13 | 51.55±49.04 | 47.15-55.96 | 60.49±48.12 | 56.17-64.81 | 1503 | 1.71×10^-6^ | **** | -0.49 |
| MetaTrans vs. GUTchetp | 14 | 52.31±48.93 | 47.91-56.7 | 60.85±47.99 | 56.54-65.16 | 1582 | 3.76×10^-6^ | **** | -0.47 |
| MetaTrans vs. GUTchetp | 15 | 53.14±48.88 | 48.75-57.53 | 61.27±47.9 | 56.97-65.57 | 1684 | 9.62×10^-6^ | **** | -0.45 |
| MetaTrans vs. GUTchetp | 16 | 53.14±48.88 | 48.75-57.53 | 61.53±47.8 | 57.23-65.82 | 1725 | 6.18×10^-6^ | **** | -0.45 |
| MetaTrans vs. GUTchetp | 17 | 54.19±48.8 | 49.8-58.57 | 62.15±47.64 | 57.87-66.43 | 1893 | 2.06×10^-5^ | **** | -0.42 |
| MetaTrans vs. GUTchetp | 18 | 54.4±48.79 | 50.02-58.78 | 62.57±47.53 | 58.30-66.84 | 1870 | 1.53×10^-5^ | **** | -0.43 |
| MetaTrans vs. GUTchetp | 19 | 54.5±48.72 | 50.12-58.87 | 63.32±47.27 | 59.08-67.57 | 1835.5 | 3.55×10^-6^ | **** | -0.46 |
| MetaTrans vs. GUTchetp | 20 | 54.91±48.68 | 50.54-59.28 | 63.86±47.05 | 59.63-68.08 | 1735 | 1.62×10^-6^ | **** | -0.48 |
| MetaPredictor vs. GUTchetp | 1 | 17.02±37.18 | 13.68-20.36 | 25.88±43.69 | 21.96-29.80 | 1953 | 1.26×10^-5^ | **** | -0.4 |
| MetaPredictor vs. GUTchetp | 2 | 26.1±43.41 | 22.2-29.99 | 34.81±47.33 | 30.56-39.06 | 1759 | 1.30×10^-5^ | **** | -0.42 |
| MetaPredictor vs. GUTchetp | 3 | 29.91±45.2 | 25.85-33.97 | 41.49±48.98 | 37.09-45.89 | 1947 | 4.42×10^-8^ | **** | -0.51 |
| MetaPredictor vs. GUTchetp | 4 | 31.51±45.84 | 27.4-35.63 | 45.6±49.47 | 41.16-50.04 | 1955.5 | 4.11×10^-10^ | **** | -0.56 |
| MetaPredictor vs. GUTchetp | 5 | 33.6±46.62 | 29.42-37.79 | 50.25±49.62 | 45.79-54.70 | 1924.5 | 1.08×10^-12^ | **** | -0.62 |
| MetaPredictor vs. GUTchetp | 6 | 34.44±46.91 | 30.23-38.65 | 52.75±49.44 | 48.31-57.19 | 1972 | 2.02×10^-14^ | **** | -0.65 |
| MetaPredictor vs. GUTchetp | 7 | 36.11±47.43 | 31.85-40.37 | 54.54±49.25 | 50.11-58.96 | 2056 | 1.48×10^-14^ | **** | -0.65 |
| MetaPredictor vs. GUTchetp | 8 | 37.52±47.79 | 33.23-41.81 | 56.12±48.99 | 51.72-60.52 | 2111 | 1.42×10^-14^ | **** | -0.65 |
| MetaPredictor vs. GUTchetp | 9 | 39.66±48.26 | 35.32-43.99 | 58.42±48.65 | 54.05-62.79 | 1611 | 7.74×10^-16^ | **** | -0.7 |
| MetaPredictor vs. GUTchetp | 10 | 40.91±48.51 | 36.55-45.26 | 58.77±48.54 | 54.41-63.13 | 1713 | 8.76×10^-15^ | **** | -0.68 |
| MetaPredictor vs. GUTchetp | 11 | 43.14±48.83 | 38.76-47.53 | 59.61±48.38 | 55.26-63.95 | 1705 | 1.78×10-^13^ | **** | -0.66 |
| MetaPredictor vs. GUTchetp | 12 | 43.94±48.86 | 39.56-48.33 | 59.99±48.28 | 55.66-64.33 | 1686 | 4.37×10-^13^ | **** | -0.65 |
| MetaPredictor vs. GUTchetp | 13 | 44.94±48.87 | 40.55-49.33 | 60.49±48.12 | 56.17-64.81 | 1912 | 2.83×10^-12^ | **** | -0.62 |
| MetaPredictor vs. GUTchetp | 14 | 45.36±48.91 | 40.97-49.75 | 60.85±47.99 | 56.54-65.16 | 1749.5 | 2.23×10^-12^ | **** | -0.64 |
| MetaPredictor vs. GUTchetp | 15 | 45.57±48.93 | 41.17-49.96 | 61.27±47.9 | 56.97-65.57 | 1863 | 2.21×10^-12^ | **** | -0.63 |
| MetaPredictor vs. GUTchetp | 16 | 45.98±48.97 | 41.59-50.38 | 61.53±47.8 | 57.23-65.82 | 1986.5 | 4.81×10^-12^ | **** | -0.61 |
| MetaPredictor vs. GUTchetp | 17 | 46.61±49.01 | 42.21-51.01 | 62.15±47.64 | 57.87-66.43 | 2095 | 7.22×10^-12^ | **** | -0.6 |
| MetaPredictor vs. GUTchetp | 18 | 47.37±49.01 | 42.97-51.77 | 62.57±47.53 | 58.3-66.840 | 2219.5 | 2.57×10^-11^ | **** | -0.59 |
| MetaPredictor vs. GUTchetp | 19 | 48.92±49.02 | 44.52-53.33 | 63.32±47.27 | 59.08-67.57 | 2319 | 1.96×10^-10^ | **** | -0.56 |
| MetaPredictor vs. GUTchetp | 20 | 50.18±49.03 | 45.78-54.58 | 63.86±47.05 | 59.63-68.08 | 2290.5 | 4.89×10^-10^ | **** | -0.56 |

**Supplementary Table S15. Statistical significance (Sig.) test results comparing MetaTrans and MetaPredictor versus GUTchetp for MaxFrag accuracy using a one-tailed McNemar’s test across Top-1 to Top-20 predictions.** Sig.: NS, not significant *p* ≥ 0.05; * *p* < 0.05; ** *p* < 0.01; *** *p* < 0.001; and **** *p* < 0.0001.

| **Models**  **(A vs. B)** | **Top-**  **N** | **Model (MetaTrans/**  **MetaPredictor)**  **(A)** | | **GUTchetp**  **(B)** | | **Test**  **statistic** | *p*  **(B>A)** | **Sig.** | **OR** |
| --- | --- | --- | --- | --- | --- | --- | --- | --- | --- |
|  |  | **Mean±SD**  **(%)** | **95% CI**  **Low-high**  **(%)** | **Mean±SD**  **(%)** | **95% CI**  **Low-high (%)** |  |  |  |  |
| MetaTrans vs. GUTchetp | 1 | 21.29±40.98 | 17.62-24.97 | 25.47±43.61 | 21.55-29.39 | 32 | 3.75×10^-2^ | * | 0.62 |
| MetaTrans vs. GUTchetp | 2 | 30.48±46.08 | 26.34-34.62 | 34.03±47.43 | 29.77-38.29 | 35 | 8.57×10^-2^ | **NS** | 0.67 |
| MetaTrans vs. GUTchetp | 3 | 36.95±48.32 | 32.61-41.29 | 41.54±49.33 | 37.12-45.97 | 40 | 3.71×10^-2^ | * | 0.65 |
| MetaTrans vs. GUTchetp | 4 | 40.29±49.1 | 35.88-44.70 | 45.51±49.85 | 41.04-49.99 | 40 | 1.87×10^-2^ | * | 0.62 |
| MetaTrans vs. GUTchetp | 5 | 42.59±49.5 | 38.14-47.03 | 50.31±50.05 | 45.82-54.81 | 34 | 3.91×10^-4^ | *** | 0.48 |
| MetaTrans vs. GUTchetp | 6 | 44.26±49.72 | 39.79-48.72 | 52.61±49.98 | 48.12-57.10 | 34 | 1.49×10^-4^ | *** | 0.46 |
| MetaTrans vs. GUTchetp | 7 | 45.72±49.87 | 41.24-50.20 | 54.28±49.87 | 49.80-58.76 | 29 | 4.61×10^-5^ | **** | 0.41 |
| MetaTrans vs. GUTchetp | 8 | 47.39±49.98 | 42.90-51.88 | 55.74±49.72 | 51.28-60.21 | 28 | 5.46×10^-5^ | **** | 0.41 |
| MetaTrans vs. GUTchetp | 9 | 48.23±50.02 | 43.73-52.72 | 58.04±49.4 | 53.60-62.47 | 25 | 1.92×10^-6^ | **** | 0.35 |
| MetaTrans vs. GUTchetp | 10 | 49.06±50.04 | 44.57-53.55 | 58.66±49.3 | 54.24-63.09 | 23 | 1.67×10^-6^ | **** | 0.33 |
| MetaTrans vs. GUTchetp | 11 | 49.48±50.05 | 44.98-53.97 | 59.92±49.06 | 55.51-64.32 | 20 | 1.14×10^-7^ | **** | 0.29 |
| MetaTrans vs. GUTchetp | 12 | 50.52±50.05 | 46.03-55.02 | 60.33±48.97 | 55.94-64.73 | 21 | 5.99×10^-7^ | **** | 0.31 |
| MetaTrans vs. GUTchetp | 13 | 51.77±50.02 | 47.28-56.27 | 61.17±48.79 | 56.79-65.55 | 23 | 2.52×10^-6^ | **** | 0.34 |
| MetaTrans vs. GUTchetp | 14 | 52.4±49.99 | 47.91-56.89 | 61.38±48.74 | 57.00-65.75 | 23 | 5.69×10^-6^ | **** | 0.35 |
| MetaTrans vs. GUTchetp | 15 | 53.44±49.93 | 48.96-57.93 | 61.8±48.64 | 57.43-66.16 | 24 | 2.37×10^-5^ | **** | 0.38 |
| MetaTrans vs. GUTchetp | 16 | 53.65±49.92 | 49.17-58.14 | 62.0±48.59 | 57.64-66.37 | 24 | 2.37×10^-5^ | **** | 0.38 |
| MetaTrans vs. GUTchetp | 17 | 54.7±49.83 | 50.22-59.17 | 62.63±48.43 | 58.28-66.98 | 26 | 7.66×10^-5^ | **** | 0.41 |
| MetaTrans vs. GUTchetp | 18 | 54.91±49.81 | 50.43-59.38 | 63.26±48.26 | 58.92-67.59 | 26 | 3.67×10^-5^ | **** | 0.39 |
| MetaTrans vs. GUTchetp | 19 | 55.11±49.79 | 50.64-59.58 | 64.09±48.02 | 59.78-68.40 | 24 | 7.35×10^-6^ | **** | 0.36 |
| MetaTrans vs. GUTchetp | 20 | 55.53±49.74 | 51.07-60.00 | 64.51±47.9 | 60.21-68.81 | 23 | 5.69×10^-6^ | **** | 0.35 |
| MetaPredictor vs. GUTchetp | 1 | 16.08±36.77 | 12.77-19.38 | 25.47±43.61 | 21.55-29.39 | 32 | 1.94×10^-5^ | **** | 0.42 |
| MetaPredictor vs. GUTchetp | 2 | 25.05±43.38 | 21.16-28.95 | 34.03±47.43 | 29.77-38.29 | 29 | 2.24×10^-5^ | **** | 0.4 |
| MetaPredictor vs. GUTchetp | 3 | 28.6±45.24 | 24.54-32.66 | 41.54±49.33 | 37.12-45.97 | 26 | 4.59×10^-9^ | **** | 0.30 |
| MetaPredictor vs. GUTchetp | 4 | 30.06±45.9 | 25.94-34.18 | 45.51±49.85 | 41.04-49.99 | 25 | 1.30×10^-11^ | **** | 0.25 |
| MetaPredictor vs. GUTchetp | 5 | 32.36±46.83 | 28.15-36.56 | 50.31±50.05 | 45.82-54.81 | 23 | 1.39×10^-14^ | **** | 0.21 |
| MetaPredictor vs. GUTchetp | 6 | 33.82±47.36 | 29.57-38.07 | 52.61±49.98 | 48.12-57.10 | 24 | 3.17×10^-15^ | **** | 0.21 |
| MetaPredictor vs. GUTchetp | 7 | 35.91±48.02 | 31.60-40.22 | 54.28±49.87 | 49.80-58.76 | 25 | 1.46×10^-14^ | **** | 0.22 |
| MetaPredictor vs. GUTchetp | 8 | 37.58±48.48 | 33.23-41.93 | 55.74±49.72 | 51.28-60.21 | 25 | 2.40×10^-14^ | **** | 0.22 |
| MetaPredictor vs. GUTchetp | 9 | 39.67±48.97 | 35.27-44.06 | 58.04±49.4 | 53.60-62.47 | 21 | 1.59×10^-15^ | **** | 0.19 |
| MetaPredictor vs. GUTchetp | 10 | 40.92±49.22 | 36.50-45.34 | 58.66±49.3 | 54.24-63.09 | 22 | 1.33×10^-14^ | **** | 0.21 |
| MetaPredictor vs. GUTchetp | 11 | 43.01±49.56 | 38.56-47.46 | 59.92±49.06 | 55.51-64.32 | 21 | 5.82×10^-14^ | **** | 0.21 |
| MetaPredictor vs. GUTchetp | 12 | 43.63±49.64 | 39.18-48.09 | 60.33±48.97 | 55.94-64.73 | 21 | 9.68×10^-14^ | **** | 0.21 |
| MetaPredictor vs. GUTchetp | 13 | 44.47±49.74 | 40.00-48.93 | 61.17±48.79 | 56.79-65.55 | 23 | 2.79×10^-13^ | **** | 0.22 |
| MetaPredictor vs. GUTchetp | 14 | 44.89±49.79 | 40.42-49.36 | 61.38±48.74 | 57.00-65.75 | 22 | 2.74×10^-13^ | **** | 0.22 |
| MetaPredictor vs. GUTchetp | 15 | 45.3±49.83 | 40.83-49.78 | 61.8±48.64 | 57.43-66.16 | 24 | 7.46×10^-13^ | **** | 0.23 |
| MetaPredictor vs. GUTchetp | 16 | 46.35±49.92 | 41.86-50.83 | 62.0±48.59 | 57.64-66.37 | 26 | 1.24×10^-11^ | **** | 0.26 |
| MetaPredictor vs. GUTchetp | 17 | 46.97±49.96 | 42.49-51.46 | 62.63±48.43 | 58.28-66.98 | 27 | 1.88×10^-11^ | **** | 0.26 |
| MetaPredictor vs. GUTchetp | 18 | 47.6±49.99 | 43.11-52.09 | 63.26±48.26 | 58.92-67.59 | 28 | 2.80×10^-11^ | **** | 0.27 |
| MetaPredictor vs. GUTchetp | 19 | 49.06±50.04 | 44.57-53.55 | 64.09±48.02 | 59.78-68.40 | 28 | 1.10×10^-10^ | **** | 0.28 |
| MetaPredictor vs. GUTchetp | 20 | 50.31±50.05 | 45.82-54.81 | 64.51±47.9 | 60.21-68.81 | 29 | 9.24×10^-10^ | **** | 0.30 |

**Supplementary Figure S30. Comparative performance analysis of 31 models trained using different combinations of five fingerprint functions compared with the CLAIRE model.** P: Pattern, M: Morgan, TT: Topological Torsion, RDKIT: RDKITFP, and AP: Atom Pair.

**Supplementary Table S16. Evaluation of fingerprints combinations for first-, second-, and third-level EC number prediction using random forest classifiers.**

| **#** | **Model** | **EC-Level** | **Fingerprint combination** | **Cross-validation**  **(Mean WAF1±SD) %** | **WAF1**  **Hidden dataset (EP)**  **(%)** |
| --- | --- | --- | --- | --- | --- |
| 1 | RF1 | 1 | M+P+TT | 78.60 ± 0.09 | 89.43 |
| 2 | RF2 | 2 | M+P+TT | 72.46 ± 0.26 | 78.55 |
| 3 | RF1 | 1 | AP+M+P+TT | 78.54 ± 0.30 | 86.99 |
| 4 | RF2 | 2 | AP+M+P+TT | 72.97 ± 0.26 | 77.53 |
| 5 | RF1 | 1 | M+P | 78.45 ± 0.20 | 88.33 |
| 6 | RF2 | 2 | M+P | 72.32 ± 0.30 | 81.02 |
| 7 | RF3 | 3 | M+P+TT | 67.93 ± 0.16 | 60.15 |

**Supplementary Table S17. Distribution of enzymatic reactions across 69 distinct EC level-2 categories.**

| **#** | **Label** | **%** | **#** | **Label** | **%** |
| --- | --- | --- | --- | --- | --- |
| 1 | 1.14 | 14.19 | 36 | 1.1 | 0.26 |
| 2 | 2.1 | 8.75 | 37 | 2.2 | 0.25 |
| 3 | N. N | 8.46 | 38 | 3.7 | 0.23 |
| 4 | 1.1 | 8.31 | 39 | 1.21 | 0.21 |
| 5 | 2.3 | 7.15 | 40 | 1.11 | 0.17 |
| 6 | 2.4 | 6.70 | 41 | 6.4 | 0.16 |
| 7 | 1.2 | 5.89 | 42 | 3.8 | 0.15 |
| 8 | 3.1 | 5.25 | 43 | 4.6 | 0.14 |
| 9 | 2.7 | 4.99 | 44 | 6.5 | 0.14 |
| 10 | 1.3 | 3.83 | 45 | 3.3 | 0.13 |
| 12 | 6.3 | 2.44 | 46 | 1.97 | 0.12 |
| 12 | 2.5 | 2.25 | 47 | 7.2 | 0.11 |
| 13 | 4.2 | 1.69 | 48 | 5.1 | 0.10 |
| 14 | 2.6 | 1.58 | 49 | 1.16 | 0.09 |
| 15 | 6.2 | 1.42 | 50 | 7.6 | 0.08 |
| 16 | 3.2 | 1.42 | 51 | 4.99 | 0.08 |
| 17 | 4.1 | 1.36 | 52 | 1.23 | 0.07 |
| 18 | 3.5 | 1.24 | 53 | 1.12 | 0.06 |
| 19 | **1.5** | **1.18** | 54 | 7.4 | 0.06 |
| 20 | 2.8 | 1.17 | 55 | 3.13 | 0.05 |
| 21 | 3.4 | 0.93 | 56 | 5.6 | 0.05 |
| 22 | 1.13 | 0.80 | 57 | 4.5 | 0.05 |
| 23 | 6.1 | 0.62 | 58 | 4.7 | 0.04 |
| 24 | 1.17 | 0.62 | 59 | 7.3 | 0.04 |
| 25 | 1.4 | 0.60 | 60 | 1.19 | 0.04 |
| 26 | 5.3 | 0.57 | 61 | 4.8 | 0.04 |
| 27 | 1.8 | 0.57 | 62 | 3.9 | 0.04 |
| 28 | 3.6 | 0.55 | 63 | 6.6 | 0.03 |
| 29 | 1.7 | 0.44 | 64 | 7.5 | 0.03 |
| 30 | 5.4 | 0.42 | 65 | 2.9 | 0.02 |
| 31 | 5.5 | 0.35 | 66 | 5.2 | 0.02 |
| 32 | 4.4 | 0.33 | 67 | 1.9 | 0.01 |
| 33 | **4.3** | **0.31** | 68 | 3.1 | 0.01 |
| 34 | 1.6 | 0.28 | 69 | 3.11 | 0.01 |
| 35 | 7.1 | 0.28 |  | | |

**Supplementary Figure S31. F1-scores across third-level of EC number subclasses in the EP dataset.** The presence of many subclasses with zero F1-scores reflects the poor performance of RF3.

**Supplementary Figure S32.** **Coverage of each confidence tier across candidate target-accuracy pairs for the RF1 model.** The yellow shaded region highlights the selected target-accuracy pair.

#

**Supplementary Figure S33.** **Coverage of each confidence tier across candidate target-accuracy pairs for the RF2 model.** The yellow shaded region highlights the selected target-accuracy pair.

**Supplementary Table S18. Model-specific margin thresholds defining High-confidence, Suggestive, and Low-confidence predictions tiers for RF1 and RF2.** Thresholds were selected on the out-of-fold (OOF) validation set to meet target accuracies (90% and 85%).

| **Model** | **Threshold of Margin** | **Reactions**  **Qty.** | **Accuracy**  **(%)** | **Coverage**  **(%)** | **Confidence Tier** |
| --- | --- | --- | --- | --- | --- |
| RF1  (M+P+TT) | Margin >= 0.4174 | 54776 | 90.00 | 69.66 | High-confidence |
|  | 0.1466 <= Margin < 0.4174 | 13960 | 65.40 | 17.75 | Suggestive |
|  | Margin < 0.1466 | 9902 | 44.95 | 12.59 | Low-confidence |
| RF2  (M+P) | margin >= 0.5831 | 36202 | 90.00 | 45.66 | High-confidence |
|  | 0.2907 <= margin < 0.5831 | 16778 | 74.21 | 21.16 | Suggestive |
|  | margin < 0.2907 | 26307 | 48.96 | 33.18 | Low-confidence |

**A**

**B**

**Supplementary Figure S34. Performance comparison of GUTchetp with reaction similarity method (A), and SIMMER (B) on the EP dataset (95 reactions).**

**Supplementary Figure S35. Statistical significance test results comparing the Reaction Similarity method and GUTchetp across Top-1 to Top-10 predictions. As the statistical significance results for Top-10 to Top-20 were identical, they were omitted.** (A) Comparison at the first EC classification level. (B) Comparison at the second EC classification level. Details of the color scheme and panel-specific annotations are provided in Supplementary Table S12.

**Supplementary Figure S36. Statistical significance test results comparing SIMMER tool versus GUTchetp across Top-1 to Top-10 predictions. As the statistical significance results for Top-10 to Top-20 were identical, they were omitted.** (A) Comparison at the first EC classification level. (B) Comparison at the second EC classification level. Details of the color scheme and panel-specific annotations are provided in Supplementary Table S12.

**Supplementary Table S19. Results of the one-tailed McNemar’s test for statistical significance (Sig.) comparing GUTchetp with the Reaction Similarity method and the SIMMER tool across Top-1 to Top-20 predictions.** Sig.: NS, not significant *p* ≥ 0.05; * *p* < 0.05; ** *p* < 0.01; *** *p* < 0.001; **** *p* < 0.0001.

| **Models**  **(A vs. B)** | **Top-**  **N** | **Model (Similarity method/**  **SIMMER)**  **(A)** | | **GUTchetp**  **(B)** | | **Test**  **statistic** | **P**  **(B>A)** | **Sig.** | **OR** |
| --- | --- | --- | --- | --- | --- | --- | --- | --- | --- |
|  |  | **Mean±SD**  **(%)** | **95% CI**  **Low-high**  **(%)** | **Mean±SD**  **(%)** | **95% CI**  **Low-high (%)** |  |  |  |  |
| Similarity vs. GUTchetp | 1 | 48.42±50.24 | 38.19-58.66 | 63.16±48.49 | 53.28-73.04 | 1 | 5.19 × 10⁻⁴ | *** | 0.07 |
| Similarity vs. GUTchetp | 2 | 55.79±49.93 | 45.62-65.96 | 64.21±48.19 | 54.39-74.03 | 3 | 5.74 × 10⁻² | NS | 0.27 |
| Similarity vs. GUTchetp | 3 | 62.11±48.77 | 52.17-72.04 | 67.37±47.14 | 57.77-76.97 | 4 | 2.67 × 10⁻¹ | NS | 0.44 |
| Similarity vs. GUTchetp | 4 | 63.16±48.49 | 53.28-73.04 | 70.53±45.83 | 61.19-79.86 | 3 | 9.23 × 10⁻² | NS | 0.3 |
| Similarity vs. GUTchetp | 5 | 64.21±48.19 | 54.39-74.03 | 73.68±44.27 | 64.67-82.7 | 2 | 2.25 × 10⁻² | * | 0.18 |
| Similarity vs. GUTchetp | 6 | 65.26±47.87 | 55.51-75.01 | 74.74±43.68 | 65.84-83.64 | 2 | 2.25 × 10⁻² | * | 0.18 |
| Similarity vs. GUTchetp | 7 | 66.32±47.51 | 56.64-75.99 | 74.74±43.68 | 65.84-83.64 | 2 | 3.86 × 10⁻² | * | 0.2 |
| Similarity vs. GUTchetp | 8 | 66.32±47.51 | 56.64-75.99 | 75.79±43.06 | 67.02-84.56 | 2 | 2.25 × 10⁻² | * | 0.18 |
| Similarity vs. GUTchetp | 9 | 67.37±47.14 | 57.77-76.97 | 75.79±43.06 | 67.02-84.56 | 2 | 3.86 × 10⁻² | * | 0.2 |
| Similarity vs. GUTchetp | 10 | 69.47±46.3 | 60.04-78.9 | 75.79±43.06 | 67.02-84.56 | 3 | 1.46 × 10⁻¹ | NS | 0.33 |
| Similarity vs. GUTchetp | 11 | 70.53±45.83 | 61.19-79.86 | 75.79±43.06 | 67.02-84.56 | 3 | 2.27 × 10⁻¹ | NS | 0.38 |
| Similarity vs. GUTchetp | 12 | 72.63±44.82 | 63.5-81.76 | 76.84±42.41 | 68.2-85.48 | 2 | 2.89 × 10⁻¹ | NS | 0.33 |
| Similarity vs. GUTchetp | 13 | 72.63±44.82 | 63.5-81.76 | 76.84±42.41 | 68.2-85.48 | 2 | 2.89 × 10⁻¹ | NS | 0.33 |
| Similarity vs. GUTchetp | 14 | 73.68±44.27 | 64.67-82.7 | 76.84±42.41 | 68.2-85.48 | 2 | 4.53 × 10⁻¹ | NS | 0.4 |
| Similarity vs. GUTchetp | 15 | 73.68±44.27 | 64.67-82.7 | 78.95±40.98 | 70.6-87.3 | 0 | 6.25 × 10⁻² | NS | 0 |
| Similarity vs. GUTchetp | 16 | 73.68±44.27 | 64.67-82.7 | 78.95±40.98 | 70.6-87.3 | 0 | 6.25 × 10⁻² | NS | 0 |
| Similarity vs. GUTchetp | 17 | 73.68±44.27 | 64.67-82.7 | 78.95±40.98 | 70.6-87.3 | 0 | 6.25 × 10⁻² | NS | 0 |
| Similarity vs. GUTchetp | 18 | 73.68±44.27 | 64.67-82.7 | 78.95±40.98 | 70.6-87.3 | 0 | 6.25 × 10⁻² | NS | 0 |
| Similarity vs. GUTchetp | 19 | 73.68±44.27 | 64.67-82.7 | 78.95±40.98 | 70.6-87.3 | 0 | 6.25 × 10⁻² | NS | 0 |
| Similarity vs. GUTchetp | 20 | 74.74±43.68 | 65.84-83.64 | 78.95±40.98 | 70.6-87.3 | 0 | 1.25 × 10⁻¹ | NS | 0 |
| SIMMER vs. GUTchetp | 1 | 28.42±45.34 | 19.18-37.66 | 63.16±48.49 | 53.28-73.04 | 6 | 5.42 × 10⁻⁷ | **** | 0.15 |
| SIMMER vs. GUTchetp | 2 | 29.47±45.83 | 20.14-38.81 | 64.21±48.19 | 54.39-74.03 | 5 | 2.50 × 10⁻⁷ | **** | 0.13 |
| SIMMER vs. GUTchetp | 3 | 29.47±45.83 | 20.14-38.81 | 67.37±47.14 | 57.77-76.97 | 5 | 4.41 × 10⁻⁸ | **** | 0.12 |
| SIMMER vs. GUTchetp | 4 | 34.74±47.87 | 24.99-44.49 | 70.53±45.83 | 61.19-79.86 | 5 | 1.41 × 10⁻⁷ | **** | 0.13 |
| SIMMER vs. GUTchetp | 5 | 35.79±48.19 | 25.97-45.61 | 73.68±44.27 | 64.67-82.7 | 4 | 1.71 × 10⁻⁸ | **** | 0.1 |
| SIMMER vs. GUTchetp | 6 | 36.84±48.49 | 26.96-46.72 | 74.74±43.68 | 65.84-83.64 | 3 | 5.63 × 10⁻⁹ | **** | 0.08 |
| SIMMER vs. GUTchetp | 7 | 36.84±48.49 | 26.96-46.72 | 74.74±43.68 | 65.84-83.64 | 3 | 5.63 × 10⁻⁹ | **** | 0.08 |
| SIMMER vs. GUTchetp | 8 | 37.89±48.77 | 27.96-47.83 | 75.79±43.06 | 67.02-84.56 | 2 | 1.49 × 10⁻⁹ | **** | 0.05 |
| SIMMER vs. GUTchetp | 9 | 37.89±48.77 | 27.96-47.83 | 75.79±43.06 | 67.02-84.56 | 2 | 1.49 × 10⁻⁹ | **** | 0.05 |
| SIMMER vs. GUTchetp | 10 | 37.89±48.77 | 27.96-47.83 | 75.79±43.06 | 67.02-84.56 | 2 | 1.49 × 10⁻⁹ | **** | 0.05 |
| SIMMER vs. GUTchetp | 11 | 37.89±48.77 | 27.96-47.83 | 75.79±43.06 | 67.02-84.56 | 2 | 1.49 × 10⁻⁹ | **** | 0.05 |
| SIMMER vs. GUTchetp | 12 | 40.0±49.25 | 29.97-50.03 | 76.84±42.41 | 68.2-85.48 | 1 | 5.53 × 10⁻¹⁰ | **** | 0.03 |
| SIMMER vs. GUTchetp | 13 | 41.05±49.45 | 30.98-51.13 | 76.84±42.41 | 68.2-85.48 | 1 | 1.08 × 10⁻⁹ | **** | 0.03 |
| SIMMER vs. GUTchetp | 14 | 41.05±49.45 | 30.98-51.13 | 76.84±42.41 | 68.2-85.48 | 1 | 1.08 × 10⁻⁹ | **** | 0.03 |
| SIMMER vs. GUTchetp | 15 | 41.05±49.45 | 30.98-51.13 | 78.95±40.98 | 70.6-87.3 | 0 | 2.91 × 10⁻¹¹ | **** | 0 |
| SIMMER vs. GUTchetp | 16 | 41.05±49.45 | 30.98-51.13 | 78.95±40.98 | 70.6-87.3 | 0 | 2.91 × 10⁻¹¹ | **** | 0 |
| SIMMER vs. GUTchetp | 17 | 41.05±49.45 | 30.98-51.13 | 78.95±40.98 | 70.6-87.3 | 0 | 2.91 × 10⁻¹¹ | **** | 0 |
| SIMMER vs. GUTchetp | 18 | 41.05±49.45 | 30.98-51.13 | 78.95±40.98 | 70.6-87.3 | 0 | 2.91 × 10⁻¹¹ | **** | 0 |
| SIMMER vs. GUTchetp | 19 | 41.05±49.45 | 30.98-51.13 | 78.95±40.98 | 70.6-87.3 | 0 | 2.91 × 10⁻¹¹ | **** | 0 |
| SIMMER vs. GUTchetp | 20 | 41.05±49.45 | 30.98-51.13 | 78.95±40.98 | 70.6-87.3 | 0 | 2.91 × 10⁻¹¹ | **** | 0 |

**Supplementary Figure S37. Overall Top-N accuracy for predicting gut microbial biotransformation profiles (both biotransformation products and their relevant enzyme in the gut microbial) using the EP dataset (95 compounds).**

**Supplementary Figure S38. Predicted bacteria genera involved in the biotransformation of Methotrexate to DAMPA.** The percentages reflect the enzyme EC: 3.4.17.11.- relative abundance in the HGRESC.

**Supplementary Figure S39. Predicted bacteria genera involved in the biotransformation of stevioside to steviol.** The percentages reflect the enzyme EC: 3.2.1.- relative abundance in the HGRESC.

**Supplementary Figure S40. Predicted bacteria genera involved in the biotransformation of DDT to DDD.** The percentages reflect the enzyme EC: 4.5.1.1 relative abundance in the HGRESC.

**Supplementary Figure S41. Predicted bacteria genera involved in the biotransformation of diclofenac glucuronide to diclofenac.** The percentages reflect the enzyme EC: 3.2.1.31 relative abundance in the HGRESC.

**Supplementary Figure S42. BLEU scores across compound-biotransformation products similarity bins expressed as percentage (%) using the PP test dataset, evaluated at (A) Top-1, (B) Top-5, (C) Top-10 and (D) Top-15.**

**Supplementary Figure S43. BLEU scores across transformation ratio bins expressed as percentage (%) using the PP test dataset, evaluated at (A) Top-1, (B) Top-5, (C) Top-10 and (D) Top-15.**

**Supplementary Figure S44. BLEU scores across mass changes bins expressed as Daltons (Da) using the PP test dataset, evaluated at (A) Top-1, (B) Top-5, (C) Top-10 and (D) Top-15.**

**Supplementary Figure S45. BLEU scores across compound atom-count bins using the PP test dataset, evaluated at (A) Top-1, (B) Top-5, (C) Top-10 and (D) Top-15.**

**Supplementary Figure S46. BLEU scores across compound SMILES-length bins (number of characters) using the PP test dataset, evaluated at (A) Top-1, (B) Top-5, (C) Top-10 and (D) Top-15.**

**Supplementary Figure S47. BLEU scores across chiral, ring-opening and ring-forming transformations expressed as percentage (%) using the PP test dataset, evaluated at (A) Top-1, (B) Top-5, (C) Top-10, (D) Top-15 and (E) Top-20.**

**Supplementary Figure S48. BLEU scores across 49 functional groups using the PP test dataset, evaluated at (A) Top-1, (B) Top-5, (C) Top-10 and (D) Top-15.**

**Supplementary Table S20. Comparison of Top-20 BLEU (%) scores for 160 chemical and structural features between GUTchetp and other tools (MetaPredictor and MetaTrans).**

- Bold “YES” marks the 143 features where GUTchetp outperforms both tools.
- Brown “Equal” indicates that GUTchetp performs equally to both tools (two features).
- Blue “NO” highlights the four features where GUTchetp performs worst.
- Red “NO” marks the 11 features where another tool outperforms GUTchetp.

| **#** | **Feature** | **GUTchetp** | **MetaPredictor** | **MetaTrans** | **Outperforms MetaPredictor** | **Outperforms MetaTrans** | **Outperforms Both** |
| --- | --- | --- | --- | --- | --- | --- | --- |
| 1 | Environmental Chemicals | 76.799 | 65.622 | 75.732 | YES | YES | **YES** |
| 2 | Polyphenols | 61.405 | 43.933 | 48.293 | YES | YES | **YES** |
| 3 | Metabolites | 67.269 | 52.711 | 50.995 | YES | YES | **YES** |
| 4 | Drugs | 65.990 | 50.965 | 53.626 | YES | YES | **YES** |
| 5 | Food Additives | 49.027 | 47.213 | 48.955 | YES | YES | **YES** |
| 6 | Medicinal Herbs | 65.760 | 57.352 | 61.822 | YES | YES | **YES** |
| 7 | Similarity [6.67 -19.55[ % | 27.083 | 8.333 | 12.500 | YES | YES | **YES** |
| 8 | Similarity [19.55 -25.52[ % | 37.222 | 15.455 | 30.038 | YES | YES | **YES** |
| 9 | Similarity [25.52 – 32.95[ % | 54.167 | 39.583 | 47.917 | YES | YES | **YES** |
| 10 | Similarity [32.95 – 40.81[ % | 60.885 | 52.457 | 50.373 | YES | YES | **YES** |
| 11 | Similarity [40.81 – 46.67[ % | 67.072 | 43.750 | 54.631 | YES | YES | **YES** |
| 12 | Similarity [46.67 – 53.85[ % | 76.391 | 55.434 | 58.600 | YES | YES | **YES** |
| 13 | Similarity [53.85 – 59.60[ % | 88.561 | 84.540 | 76.502 | YES | YES | **YES** |
| 14 | Similarity [59.60 – 65.56[ % | 80.784 | 68.185 | 69.440 | YES | YES | **YES** |
| 15 | Similarity [65.56 – 75.00[ % | 83.992 | 67.333 | 71.087 | YES | YES | **YES** |
| 16 | Similarity [75.00- 94.23] % | 64.054 | 66.810 | 56.718 | NO | YES | **NO** |
| 17 | Transformation ratios [11.76 - 42.86 [ % | 62.500 | 27.083 | 41.667 | YES | YES | **YES** |
| 18 | Transformation ratios [42.86 – 52.88[ % | 72.490 | 58.811 | 58.963 | YES | YES | **YES** |
| 19 | Transformation ratios [52.88 – 61.39[ % | 64.583 | 66.667 | 57.853 | NO | YES | **NO** |
| 20 | Transformation ratios [61.39 – 71.43[ % | 71.402 | 61.567 | 55.451 | YES | YES | **YES** |
| 21 | Transformation ratios [71.43 – 84.62[ % | 63.790 | 61.196 | 62.628 | YES | YES | **YES** |
| 22 | Transformation ratios [84.62 – 92.31[ % | 77.891 | 58.846 | 61.325 | YES | YES | **YES** |
| 23 | Transformation ratios [92.31 – 96.09[ % | 76.375 | 51.047 | 51.545 | YES | YES | **YES** |
| 24 | Transformation ratios [96.09 – 100[ % | 52.179 | 38.940 | 43.750 | YES | YES | **YES** |
| 25 | Transformation ratios [100 – 105[ % | 57.624 | 31.205 | 51.840 | YES | YES | **YES** |
| 26 | Transformation ratios [105 – 291.67] % | 41.407 | 46.589 | 42.841 | NO | NO | **NO** |
| 27 | Exact mass changes [-914.05 - -308.11[ Da | 75.441 | 46.164 | 38.061 | YES | YES | **YES** |
| 28 | Exact mass changes [-308.11 - -176.03[ Da | 79.006 | 58.210 | 71.253 | YES | YES | **YES** |
| 29 | Exact mass changes [-176.03 - -149.01[ Da | 78.795 | 80.435 | 72.415 | NO | YES | **NO** |
| 30 | Exact mass changes [-149.01 - -89.03[ Da | 44.634 | 43.620 | 44.634 | YES | Equal | **NO** |
| 31 | Exact mass changes [-89.03 - -42.01] Da | 62.500 | 52.083 | 58.677 | YES | YES | **YES** |
| 32 | Exact mass changes [-41.97 - -18.01] Da | 66.805 | 44.244 | 51.695 | YES | YES | **YES** |
| 33 | Exact mass changes [-15.99 - -14.02[ Da | 87.383 | 68.552 | 61.240 | YES | YES | **YES** |
| 34 | Exact mass changes [-14.02 – 2.02] Da | 60.818 | 33.162 | 49.903 | YES | YES | **YES** |
| 35 | Exact mass changes [2.02 – 14.96] Da | 28.762 | 28.762 | 34.235 | Equal | NO | **NO** |
| 36 | Exact mass changes [15.99 – 343.16] Da | 43.490 | 45.234 | 42.164 | NO | YES | **NO** |
| 37 | Number of Atoms [4 – 12] | 49.298 | 45.022 | 52.165 | YES | NO | **NO** |
| 38 | Number of Atoms [13 – 15] | 76.744 | 70.020 | 65.356 | YES | YES | **YES** |
| 39 | Number of Atoms [16 – 19] | 62.001 | 49.726 | 46.591 | YES | YES | **YES** |
| 40 | Number of Atoms [20 – 21] | 52.644 | 34.443 | 44.262 | YES | YES | **YES** |
| 41 | Number of Atoms [22 – 23] | 67.940 | 59.871 | 58.141 | YES | YES | **YES** |
| 42 | Number of Atoms [24 – 26] | 59.898 | 36.627 | 48.492 | YES | YES | **YES** |
| 43 | Number of Atoms [27 – 29] | 82.927 | 62.116 | 70.467 | YES | YES | **YES** |
| 44 | Number of Atoms [30 – 33] | 68.303 | 52.131 | 60.154 | YES | YES | **YES** |
| 45 | Number of Atoms [34 – 44] | 63.282 | 54.585 | 57.201 | YES | YES | **YES** |
| 46 | Number of Atoms [45 – 87] | 67.212 | 48.390 | 31.505 | YES | YES | **YES** |
| 47 | Compound SMILES length [6 – 22[ | 51.543 | 49.571 | 51.655 | YES | NO | **NO** |
| 48 | Compound SMILES length [22 – 27[ | 66.000 | 56.003 | 53.908 | YES | YES | **YES** |
| 49 | Compound SMILES length [27 – 34[ | 57.357 | 45.833 | 46.539 | YES | YES | **YES** |
| 50 | Compound SMILES length [34 – 38[ | 64.583 | 35.494 | 53.489 | YES | YES | **YES** |
| 51 | Compound SMILES length [38 – 42[ | 60.636 | 50.733 | 41.988 | YES | YES | **YES** |
| 52 | Compound SMILES length [42 – 45[ | 59.137 | 50.273 | 57.706 | YES | YES | **YES** |
| 53 | Compound SMILES length [45 - 52[ | 79.197 | 62.340 | 72.346 | YES | YES | **YES** |
| 54 | Compound SMILES length [52 – 61[ | 73.916 | 51.199 | 61.725 | YES | YES | **YES** |
| 55 | Compound SMILES length [61 – 86[ | 65.375 | 54.958 | 55.379 | YES | YES | **YES** |
| 56 | Compound SMILES length [86 – 168[ | 62.107 | 45.366 | 33.051 | YES | YES | **YES** |
| 57 | without ring opening | 63.902 | 49.707 | 54.714 | YES | YES | **YES** |
| 58 | ring opening | 64.122 | 50.815 | 50.123 | YES | YES | **YES** |
| 59 | without ring formation | 65.757 | 51.199 | 53.946 | YES | YES | **YES** |
| 60 | Ring formation | 29.068 | 29.917 | 29.432 | NO | NO | **NO** |
| 61 | without chirality | 64.350 | 50.695 | 52.626 | YES | YES | **YES** |
| 62 | Chirality | 58.053 | 41.497 | 55.150 | YES | YES | **YES** |
| 63 | Bicyclic (Absent) | 64.994 | 54.949 | 57.988 | YES | YES | **YES** |
| 64 | Bicyclic (Present) | 63.202 | 46.388 | 48.624 | YES | YES | **YES** |
| 65 | Aromatic carboxylic acid (Absent) | 62.812 | 49.354 | 51.885 | YES | YES | **YES** |
| 66 | Aromatic carboxylic acid (Present) | 84.615 | 64.522 | 68.155 | YES | YES | **YES** |
| 67 | H pyrrole nitrogen (Absent) | 63.920 | 49.941 | 52.352 | YES | YES | **YES** |
| 68 | H pyrrole nitrogen (Present) | 65.925 | 56.211 | 63.435 | YES | YES | **YES** |
| 69 | Imines (Absent) | 64.419 | 50.731 | 53.283 | YES | YES | **YES** |
| 70 | Imines (Present) | 54.746 | 38.095 | 41.554 | YES | YES | **YES** |
| 71 | N functional groups attached to aromatics (Absent) | 64.218 | 50.64 | 53.132 | YES | YES | **YES** |
| 72 | N functional groups attached to aromatics (Present) | 59.135 | 40.087 | 44.849 | YES | YES | **YES** |
| 73 | Primary amines (Absent) | 64.972 | 50.744 | 53.276 | YES | YES | **YES** |
| 74 | Primary amines (Present) | 53.828 | 44.280 | 47.485 | YES | YES | **YES** |
| 75 | Secondary amines (Absent) | 64.257 | 50.056 | 52.820 | YES | YES | **YES** |
| 76 | Secondary amines (Present) | 62.491 | 50.870 | 52.470 | YES | YES | **YES** |
| 77 | Tertiary amines (Present) | 74.513 | 61.041 | 64.208 | YES | YES | **YES** |
| 78 | Tertiary amines (Absent) | 59.011 | 45.029 | 47.348 | YES | YES | **YES** |
| 79 | XCCNR groups (Absent) | 63.635 | 49.431 | 51.880 | YES | YES | **YES** |
| 80 | XCCNR groups (Present) | 76.923 | 76.923 | 84.615 | Equal | NO | **NO** |
| 81 | Aliphatic carboxylic acids (Absent) | 63.996 | 48.907 | 51.253 | YES | YES | **YES** |
| 82 | Aliphatic carboxylic acids (Present) | 63.993 | 55.520 | 59.143 | YES | YES | **YES** |
| 83 | Aliphatic hydroxyl groups (Present) | 63.399 | 50.676 | 52.749 | YES | YES | **YES** |
| 84 | Aliphatic hydroxyl groups (Absent) | 64.384 | 49.852 | 52.781 | YES | YES | **YES** |
| 85 | Aliphatic hydroxyl groups excluding tert OH (Present) | 63.961 | 50.274 | 52.415 | YES | YES | **YES** |
| 86 | Aliphatic hydroxyl groups excluding tert OH (Absent) | 64.017 | 50.117 | 52.987 | YES | YES | **YES** |
| 87 | Alkyl halides (Absent) | 63.539 | 49.968 | 52.381 | YES | YES | **YES** |
| 88 | Alkyl halides (Present) | 100 | 66.667 | 83.333 | YES | YES | **YES** |
| 89 | Allylic oxidation sites excluding steroid dienone (Absent) | 64.902 | 51.060 | 54.579 | YES | YES | **YES** |
| 90 | Allylic oxidation sites excluding steroid dienone (Present) | 57.780 | 44.128 | 40.364 | YES | YES | **YES** |
| 91 | Amides (Absent) | 64.461 | 49.991 | 52.483 | YES | YES | **YES** |
| 92 | Amides (Present) | 60.168 | 51.701 | 55.114 | YES | YES | **YES** |
| 93 | Amidine groups (Absent) | 64.097 | 50.286 | 53.127 | YES | YES | **YES** |
| 94 | Amidine groups (Present) | 57.143 | 42.857 | 28.571 | YES | YES | **YES** |
| 95 | Anilines (Absent) | 64.090 | 50.197 | 53.154 | YES | YES | **YES** |
| 96 | Anilines (Present) | 63.366 | 50.042 | 50.222 | YES | YES | **YES** |
| 97 | Aromatic amines (Absent) | 63.920 | 49.941 | 52.352 | YES | YES | **YES** |
| 98 | Aromatic amines (Present) | 65.925 | 56.211 | 63.435 | YES | YES | **YES** |
| 99 | Aromatic hydroxyl groups (Absent) | 64.974 | 53.627 | 56.109 | YES | YES | **YES** |
| 100 | Aromatic hydroxyl groups (Present) | 63.198 | 47.368 | 50.048 | YES | YES | **YES** |
| 101 | Aromatic nitrogen (Absent) | 63.854 | 49.626 | 52.191 | YES | YES | **YES** |
| 102 | Aromatic nitrogen (Present) | 64.898 | 53.684 | 56.448 | YES | YES | **YES** |
| 103 | Aryl methyl sites for hydroxylation (Absent) | 63.489 | 48.946 | 52.160 | YES | YES | **YES** |
| 104 | Aryl methyl sites for hydroxylation (Present) | 69.552 | 63.690 | 59.441 | YES | YES | **YES** |
| 105 | Azo groups (Absent) | 61.833 | 48.502 | 51.297 | YES | YES | **YES** |
| 106 | Azo groups (Present) | 91.429 | 71.429 | 71.429 | YES | YES | **YES** |
| 107 | Benzene rings (Absent) | 62.534 | 49.15 | 53.007 | YES | YES | **YES** |
| 108 | Benzene rings (Present) | 64.386 | 50.451 | 52.705 | YES | YES | **YES** |
| 109 | Benzodiazepines with no additional fused rings (Absent) | 64.111 | 50.502 | 52.940 | YES | YES | **YES** |
| 110 | Benzodiazepines with no additional fused rings (Present) | 59.061 | 36.364 | 45.455 | YES | YES | **YES** |
| 111 | Carbonyl O (Absent) | 67.458 | 51.882 | 53.720 | YES | YES | **YES** |
| 112 | Carbonyl O (Present) | 60.796 | 48.602 | 51.890 | YES | YES | **YES** |
| 113 | Carbonyl O excluding COOH (Absent) | 67.970 | 53.134 | 54.915 | YES | YES | **YES** |
| 114 | Carbonyl O excluding COOH (Present) | 56.770 | 44.803 | 48.867 | YES | YES | **YES** |
| 115 | Carboxylic acids (Absent) | 62.647 | 48.081 | 50.309 | YES | YES | **YES** |
| 116 | Carboxylic acids (Present) | 68.310 | 56.889 | 60.642 | YES | YES | **YES** |
| 117 | Cyclic esters lactones (Absent) | 65.079 | 50.900 | 53.286 | YES | YES | **YES** |
| 118 | Cyclic esters lactones (Present) | 42.507 | 35.844 | 42.507 | YES | Equal | **NO** |
| 119 | Esters (Absent) | 63.823 | 49.411 | 50.994 | YES | YES | **YES** |
| 120 | Esters (Present) | 64.967 | 54.506 | 62.798 | YES | YES | **YES** |
| 121 | Ether oxygens including phenoxy (Absent) | 61.002 | 47.862 | 47.492 | YES | YES | **YES** |
| 122 | Ether oxygens including phenoxy (Present) | 66.760 | 52.316 | 57.642 | YES | YES | **YES** |
| 123 | Halogens (Absent) | 63.499 | 50.055 | 52.031 | YES | YES | **YES** |
| 124 | Halogens (Present) | 68.779 | 51.351 | 59.884 | YES | YES | **YES** |
| 125 | Hydroxylamine groups (Absent) | 63.852 | 49.968 | 52.804 | YES | YES | **YES** |
| 126 | Hydroxylamine groups (Present) | 75.294 | 66.667 | 50.000 | YES | YES | **YES** |
| 127 | Imidazole rings (Absent) | 64.112 | 50.157 | 53.192 | YES | YES | **YES** |
| 128 | Imidazole rings (Present) | 60.130 | 50.842 | 38.702 | YES | YES | **YES** |
| 129 | Ketones (Absent) | 66.501 | 52.900 | 55.748 | YES | YES | **YES** |
| 130 | Ketones (Present) | 48.034 | 32.834 | 33.791 | YES | YES | **YES** |
| 131 | Ketones excluding diaryl a b unsat dienones heteroatom on Calpha (Absent) | 65.640 | 52.832 | 55.141 | YES | YES | **YES** |
| 132 | Ketones excluding diaryl a b unsat dienones heteroatom on Calpha (Present) | 51.313 | 29.712 | 34.480 | YES | YES | **YES** |
| 133 | Methoxy groups OCH3 (Absent) | 62.758 | 49.827 | 50.206 | YES | YES | **YES** |
| 134 | Methoxy groups OCH3 (Present) | 69.417 | 51.712 | 63.998 | YES | YES | **YES** |
| 135 | Nitro benzene ring substituents (Absent) | 63.685 | 49.967 | 52.026 | YES | YES | **YES** |
| 136 | Nitro benzene ring substituents (Present) | 70.440 | 54.545 | 68.182 | YES | YES | **YES** |
| 137 | Nitro groups (Absent) | 63.947 | 50.077 | 52.381 | YES | YES | **YES** |
| 138 | Nitro groups (Present) | 64.803 | 51.852 | 59.259 | YES | YES | **YES** |
| 139 | Non ortho nitro benzene ring substituents (Absent) | 63.783 | 49.967 | 52.226 | YES | YES | **YES** |
| 140 | Non ortho nitro benzene ring substituents (Present) | 69.426 | 55.556 | 66.667 | YES | YES | **YES** |
| 141 | Para hydroxylation sites (Absent) | 63.765 | 49.217 | 52.292 | YES | YES | **YES** |
| 142 | Para hydroxylation sites (Present) | 67.216 | 63.593 | 59.417 | YES | YES | **YES** |
| 143 | Phenolic OH excluding ortho intramolecular Hbond substituents (Absent) | 66.678 | 55.304 | 57.777 | YES | YES | **YES** |
| 144 | Phenolic OH excluding ortho intramolecular Hbond substituents (Present) | 61.537 | 45.480 | 48.181 | YES | YES | **YES** |
| 145 | Phenols (Absent) | 65.925 | 54.139 | 56.701 | YES | YES | **YES** |
| 146 | Phenols (Present) | 62.343 | 46.783 | 49.400 | YES | YES | **YES** |
| 147 | Piperdine rings (Absent) | 64.204 | 50.122 | 52.763 | YES | YES | **YES** |
| 148 | Piperdine rings (Present) | 53.072 | 53.072 | 53.072 | Equal | Equal | **Equal** |
| 149 | Pyridine rings (Absent) | 63.754 | 49.533 | 52.409 | YES | YES | **YES** |
| 150 | Pyridine rings (Present) | 68.785 | 62.940 | 59.898 | YES | YES | **YES** |
| 151 | Quaternary nitrogen (Absent) | 64.037 | 50.073 | 52.692 | YES | YES | **YES** |
| 152 | Quaternary nitrogen (Present) | 60.000 | 60.000 | 60.000 | Equal | Equal | **Equal** |
| 153 | Sulfonamides (Absent) | 64.141 | 49.967 | 52.84 | YES | YES | **YES** |
| 154 | Sulfonamides (Present) | 58.333 | 58.333 | 50.000 | Equal | YES | **NO** |
| 155 | Tert alicyclic amines no heteroatoms not quinine like bridged N (Absent) | 63.702 | 49.589 | 52.449 | YES | YES | **YES** |
| 156 | Tert alicyclic amines no heteroatoms not quinine like bridged N (Present) | 77.765 | 77.765 | 67.765 | Equal | YES | **NO** |
| 157 | Thioether (Absent) | 63.867 | 50.181 | 53.005 | YES | YES | **YES** |
| 158 | Thioether (Present) | 70.000 | 50.000 | 41.651 | YES | YES | **YES** |
| 159 | Unbranched alkanes of at least 4 members excludes halogenated alkanes (Absent) | 63.975 | 49.968 | 52.508 | YES | YES | **YES** |
| 160 | Unbranched alkanes of at least 4 members excludes halogenated alkanes (Present) | 64.966 | 60.000 | 64.966 | YES | Equal | **NO** |

**Supplementary Table S21. Reproducibility manifest for GUTchetp.** Summary of the metadata and computational resources required to reproduce the experiments reported in this study.

| **Category** | **Item** | **Value** | **Location / Availability** |
| --- | --- | --- | --- |
| **Repository** | Web server | ai-chembiome | <https://web.app.ufz.de/ai-chembiome/> |
|  | Source code repository | GUTchetp | <https://git.ufz.de/masun/GUTchetp> |
|  | Repository version | Release v1.0.0 | Repository |
|  | Git commit | cbf7d98c | Repository |
| **Computational environment** | Operating system | Rocky Linux 9.8 (Blue Onyx)  Kernel: Linux 5.14.0-687.26.1.el9_8.x86_64 |  |
|  | Python | 3.11 | environment.yml |
|  | Deep learning libraries | torch (2.4.1)  OpenNMT-py (2.2.0) |  |
|  | Cheminformatics libraries | Pubchempy (1.0.4)  Rdkit (2024.9.5)  Selfies (2.2.0) |  |
|  | Machine learning libraries | scikit-learn (1.6.1) |  |
|  | Scientific data manipulation and visualization | Numpy (1.26.4)  Pandas (1.5.3)  Scipy (1.15.1)  scikit-learn (1.6.1)  matplotlib (3.10.0) |  |
|  | GPU | nvidia-a100 or tesla-v100 |  |
|  | CUDA | 11.7.0 |  |
|  | SLURM | 25.11.7 |  |
| **GUTchetp: First Component for Gut Biotransformation Prediction** | | | |
| **Repro-ducibility** | Random seed | 3435 | Fixed for all reported experiments |
|  | Partition IDs | fold1–fold10 | Datasets/folds/ |
|  | Train/validationsplits | Defined by the partition files (fold1–fold10) | Datasets/folds/ |
| **Datasets** | Public datasets | BRD, MetaNetX, PP, EP | Datasets/ |
|  | MetaNetX fine-tuning data | SRP and MRP | Datasets/MetaNetX/ |
|  | USPTO fine-tuning data | SRP and MRP | Datasets/USPTO/ |
| **Models** | Public checkpoints | M4.pt, M31.pt, MetaNetX_Singlerepresentation.pt, MetaNetX_Multirepresentation.pt, USPTO_Singlerepresentation.pt, USPTO_Multirepresentation.pt | [Google Drive](https://drive.google.com/drive/folders/13sW_rj7YoBH73MSb3zNqGribPsm3Vuyh?usp=sharing) (repository release v1.0.0) |
|  | Additional GUTchetp checkpoints | Remaining trained models | Available from the corresponding author upon reasonable request |
| **Scripts** | Demo notebook | Demo.ipynb | predict_gut_biotransformation_products/ |
|  | Fine-tuning pipelines | SRP and MRP pipelines:  finetuning_translating_SRP_all.sh  finetuning_translating_MRP_all.sh | predict_gut_biotransformation_products/ |
|  | Configuration templates | finetuning_SRP.yaml.template, finetuning_MRP.yaml.template | predict_gut_biotransformation_products/ |
| **GUTchetp: Second Component for Gut Reactions and Species Prediction** | | | |
| Datasets and trained models are available from the corresponding author upon request. | | | |
